# Autologous adoptive immune-cell therapy elicited a durable response with enhanced immune reaction signatures in patients with recurrent glioblastoma: An open label, phase I/IIa trial

**DOI:** 10.1371/journal.pone.0247293

**Published:** 2021-03-10

**Authors:** Jaejoon Lim, YoungJoon Park, Ju Won Ahn, JeongMin Sim, Su Jung Kang, Sojung Hwang, Jin Chun, Hyejeong Choi, Sang Heum Kim, Duk-Hee Chun, Kyoung Su Sung, KyuBum Kwack, Kyunggi Cho

**Affiliations:** 1 Department of Neurosurgery, Bundang CHA Medical Center, CHA University College of Medicine, Seongnam, Republic of Korea; 2 Department of Biomedical Science, College of Life Science, CHA University, Seongnam, Republic of Korea; 3 Global Research Supporting Center, Bundang CHA Medical Center, CHA University College of Medicine, Seongnam, Republic of Korea; 4 Department of Radiology, Bundang CHA Medical Center, CHA University College of Medicine, Seongnam, Republic of Korea; 5 Department of Anesthesiology and Pain Medicine, Bundang CHA Medical Center, CHA University College of Medicine, Seongnam, Republic of Korea; 6 Department of Neurosurgery, Dong-A University Hospital, Dong-A University College of Medicine, Busan, Republic of Korea; Cardiff University, UNITED KINGDOM

## Abstract

Glioblastoma multiforme (GBM) is an aggressive malignancy classified by the World Health Organization as a grade IV glioma. Despite the availability of aggressive standard therapies, most patients experience recurrence, for which there are currently no effective treatments. We aimed to conduct a phase I/IIa clinical trial to investigate the safety and efficacy of adoptive, *ex-vivo-*expanded, and activated natural killer cells and T lymphocytes from peripheral blood mononuclear cells of patients with recurrent GBM. This study was a single-arm, open-label, investigator-initiated trial on 14 patients recruited between 2013 and 2017. The immune cells were administered via intravenous injection 24 times at 2-week intervals after surgical resection or biopsy. The safety and clinical efficacy of this therapy was examined by assessing adverse events and comparing 2-year overall survival (OS). Transcriptomic analysis of tumor tissues was performed using NanoString to identify the mechanism of therapeutic efficacy. No grade 4 or 5 severe adverse events were observed. The most common treatment-related adverse events were grade 1 or 2 in severity. The most severe adverse event was grade 3 fever. Median OS was 22.5 months, and the median progression-free survival was 10 months. Five patients were alive for over 2 years and showed durable response with enhanced immune reaction transcriptomic signatures without clinical decline until the last follow-up after completion of the therapy. In conclusion, autologous adoptive immune-cell therapy was safe and showed durable response in patients with enhanced immune reaction signatures. This therapy may be effective for recurrent GBM patients with high immune response in their tumor microenvironments. **Trial registration:** The Korea Clinical Research Information Service database: KCT0003815, Registered 18 April 2019, retrospectively registered.

## Introduction

Glioblastoma multiforme (GBM) is an aggressive malignancy classified by the World Health Organization as a grade IV glioma, with an average overall survival (OS) of < 20 months after diagnosis as primary GBM [1]. Despite the availability of aggressive standard therapies, such as radiation and temozolomide after maximal surgical resection, most patients experience recurrence, for which there are currently no effective treatments, leading to poor OS (< 12 months) [2]. Several clinical trials have investigated aggressive, targeted immunotherapies based on drugs or antibodies, such as immune checkpoint inhibitors. However, drug- and antibody-based therapies have not demonstrated any significant benefits in terms of OS compared to standard therapies in recurrent GBM patients, which is commonly less than 8–12 months [3–6]. The GBM microenvironment is characterized by immunosuppression owing to intrinsic components, such as tumor-associated macrophages, reactive gliosis, and a cold-tumor phenotype [7]. Therefore, there is a need to re-evaluate immunotherapeutic strategies for GBM. Recently, vaccine-based therapies based on genetically modified oncolytic viruses and vaccine-based immunotherapies, including recombinant adenovirus [8], polio-rhinovirus chimera [9], and several dendritic cell vaccines [10,11], were shown to elicit durable responses of more than three years of survival in some patients with recurrent GBM or glioma. In addition, Ishikawa *et al*. reported stable disease or lack of severe toxicity in 9 out of 16 GBM patients after therapy with immune cells expanded *ex vivo* from peripheral blood mononuclear cells (PBMCs) [12].

In this study, we aimed to conduct a single-arm, open-label phase I/IIa clinical trial by examining the safety and efficacy of the administration of adoptive immune cells including natural killer (NK) cells and T lymphocytes, expanded from human PBMCs under *ex vivo* conditions, to patients with recurrent GBM. In addition, we aimed to identify the mechanism underlying the clinical efficacy of the adoptive immune-cell therapy.

## Materials and methods

### Study design

This was a prospective, open-label, single-arm, investigator-initiated phase I/IIa clinical trial designed to assess the safety, clinical efficacy, and action mechanism of adoptive, activated immune cells expanded from PBMCs of patients with recurrent GBM. The study included 14 patients who received adoptive immune cell therapy from February 2013 to August 2018. All patients were followed up until October 2019. The study was approved by the Institutional Review Board of Bundang CHA Medical Center (2012-12-172) in December 2012, and the complete date range for patient recruitment and follow-up was approved on July 23, 2018. Data from patients who not included in the trial but attending the same hospital from January 2010 to December 2017 were used as historical controls. Detailed information is provided in S6 Table in S1 File.

Informed consent in written form was received from all participants prior to their inclusion in the study. We were not aware of the international clinical trial registration process during our first clinical trial. After reviewing international guidelines, this study was retrospectively registered (The Korea Clinical Research Information Service database: KCT0003815). We confirm that all ongoing and related trials related to this intervention are registered.

### Inclusion and exclusion criteria for participant recruitment

Patients were recruited by providing relevant information to potential trial participants who visited Bundang CHA medical center and by advertising the study on the hospital website. Patient recruitment and research progress were conducted in the Bundang CHA medical center.

Potentially eligible patients for the trial were those in whom standard therapy had failed after a recurrence of GBM based on MRI scan. Detailed inclusion and exclusion criteria are listed in S1 Table in S1 File. Demographic details of participants were described in S2 and S3 Tables in S1 File.

### Endpoints

The primary endpoint of this study was the evaluation of safety and treatment-related adverse events of adoptive immune cell therapy in patients with recurrent GBM. The secondary endpoint was to evaluate the potential efficacy and mechanism of action of the adoptive immune cell therapy.

### *Ex vivo* expansion and activation of immune cells and method of administration

*Ex vivo* expansion was performed for two weeks according to Good Manufacturing Practice guidelines as follows: a final volume of 100 mL of expanded immune cells (approximately 2–6 × 10^9^ cells) was administered by intravenous injection to patients with recurrent GBM (detailed protocols and the results of *ex vivo* expansion and *in vitro* evaluation of expanded immune cells are provided in S1–S5 Figs in S1 File). The injection of immune cells was administered once every two weeks for a maximum of 24 doses (detailed information on expansion of immune cells before administration to patients is provided in S4 Table in S1 File). All patients with confirmed tumor progression during adoptive immune cell therapy had the treatment stopped immediately.

### Assessment of safety and efficacy

The adverse events, vital signs, and results of physical examination were evaluated. Safety was assessed according to the National Cancer Institute Common Terminology Criteria for Adverse Events (version 4.0). We performed the immunotherapy radiographic response assessment using the immunotherapy Response Assessment in Neuro-Oncology (iRANO) criteria [13]. In addition, we assessed clinical response and stratified patients into good responders and poor responders based on their 2-year survival time. Patients with a durable response and who survived for over 2 years were classified as good responders.

### Imaging analysis

Tumors were evaluated at screening and every three months thereafter using a brain magnetic resonance imaging (MRI) scanner. If pseudoprogression and radiation necrosis were difficult to distinguish from progression based on the MRI, the patient was re-evaluated within six months. Images were assessed according to the iRANO criteria. Imaging techniques, assessment details, and data are described in the Supplementary Methods in S1 File. We used post-operation MR scans (<72 h after surgical resection) as the baseline MR scan.

### NanoString assay

RNA was extracted from the tumor tissues of the patients with recurrent GBM prior to adoptive immune cell therapy and analyzed using the NanoString nCounter Analysis System: the nCounter PanCancer IO 360^TM^ Panel (NanoString Technologies, Inc., WA, USA). The GeNorm algorithm [14] in nCounter Advanced Analysis (version 2.0.115) was used to normalize raw gene expression values (NanoString Technologies®, Inc. nSolver™ AdavancedAnalysisSoftware.) for 750 genes; the data were log_2-_ transformed. Detailed information about the NanoString analyses are provided in the Supplemental Appendix in S1 File. NanoString data have been uploaded to GEO dataset in NCBI (GSE142693).

### Statistical analysis

Adverse events were reported as those with the highest graded score and frequency. OS was estimated from the time of GBM recurrence until death. Two-year OS was used to determine efficacy in patients with recurrent GBM who were treated with adoptive immune cell therapy. All statistical analyses were performed with R studio (version: 1.1.456), and a p value of < 0.05 indicated statistical significance. Cox-regression, t-test and paired t-test (two-sided) were performed for *in-vitro* experiments or transcriptomic analyses with NanoString data. Wilcoxon signed rank test was conducted for analyzing with immunohistochemical staining data. Agglomerative clustering with Ward method was performed for analyzing with GBM dataset in TCGA.

The aims of this single-arm study were to evaluate safety and adverse events and to reveal minimal efficacy and mode of action for additional phase 2 clinical trials; therefore, the sample size was not measured through statistical power. In addition, multiple comparisons were not performed owing to a small number of patients.

## Results

### General information about participants

**Fig 1:**
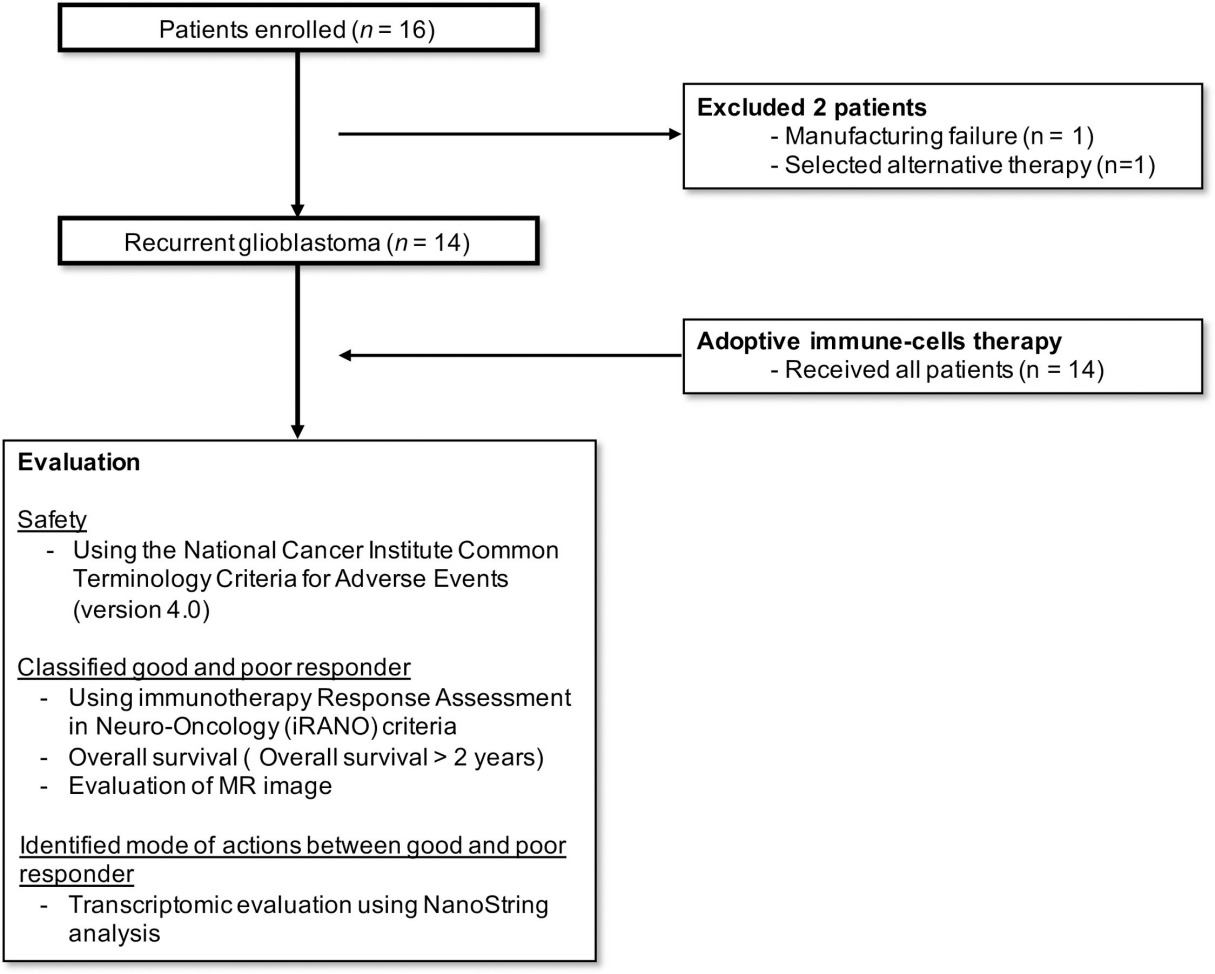
Participant enrollment flow diagram. MR; magnetic resonance.

A total 16 patients were enrolled in this clinical trial. Among 16 patients, 2 patients did not receive adoptive immune cell therapy because of manufacturing failure and election of alternative therapy (Fig 1). As a result, 14 patients, who fulfilled the inclusion and exclusion criteria (S1 Table in S1 File), received adoptive immune cell therapy (S4 Table in S1 File).

The average age-at-diagnosis in the recurrent GBM, adoptive-immune-cell-treated group was 53 years (standard deviation: 9.5, age range: 27–69). Among the 14 patients, 42.9% and 57.1% were male and female, respectively. The isocitrate dehydrogenase *IDH1* mutation, R132H, was detected in one patient. Adjuvant chemotherapy and radiotherapy were used as a salvage treatment; detailed baseline information and clinical characteristics of the 14 patients are provided in S2 Table in S1 File.

### Safety of adoptive immune cell therapy

There were no grade 4 or 5 severe adverse events associated with the adoptive immune cell therapy. In patients treated with adoptive immune cells, the most common treatment-related adverse events were grade 1 or 2 in severity (Table 1 and S5 Table in S1 File). The most severe therapy-related adverse event was grade 3 fever (Table 1 and S5 Table in S1 File). The most frequent adverse events were fever, anorexia, hot flushes, chills, injection site reaction, and autoimmune disorder (Table 1). Adverse events not considered related to adoptive immune cell therapy by the investigators were those caused by other chemotherapeutic agents and tumor progression. Among these, grade 4 and 5 adverse events (2.7%) included leukocytopenia, thrombocytopenia, brain swelling, and hydrocephalus (S5 Table in S1 File). Leukocytopenia and thrombocytopenia events were caused by chemotherapeutic agents. Brain swelling and hydrocephalus were caused by tumor progression. Regardless of relatedness to the investigational drug, all adverse events that occurred during this study are described in S5 Table in S1 File.

**Table 1:** Adoptive immune-cell therapy-related adverse events.

Adverse event	Patient	Event time	Grade*
**General disorders and administration site conditions**			
Fever	A1	9	3
	A7	1	1
Injection site reaction	A1	1	2
Chills	A7	1	1
**Blood and lymphatic system disorders**			
Anemia	A3	3	2
**Metabolism and nutrition disorders**			
Anorexia	A3	1	2
**Vascular disorders**			
Hot flushes	A6	1	1
**Immune system disorders**			
Autoimmune disorder	A13	1	2

*The highest-grade event in each adoptive immune cells-treated patient.

### Clinical efficacy

**Fig 2:**
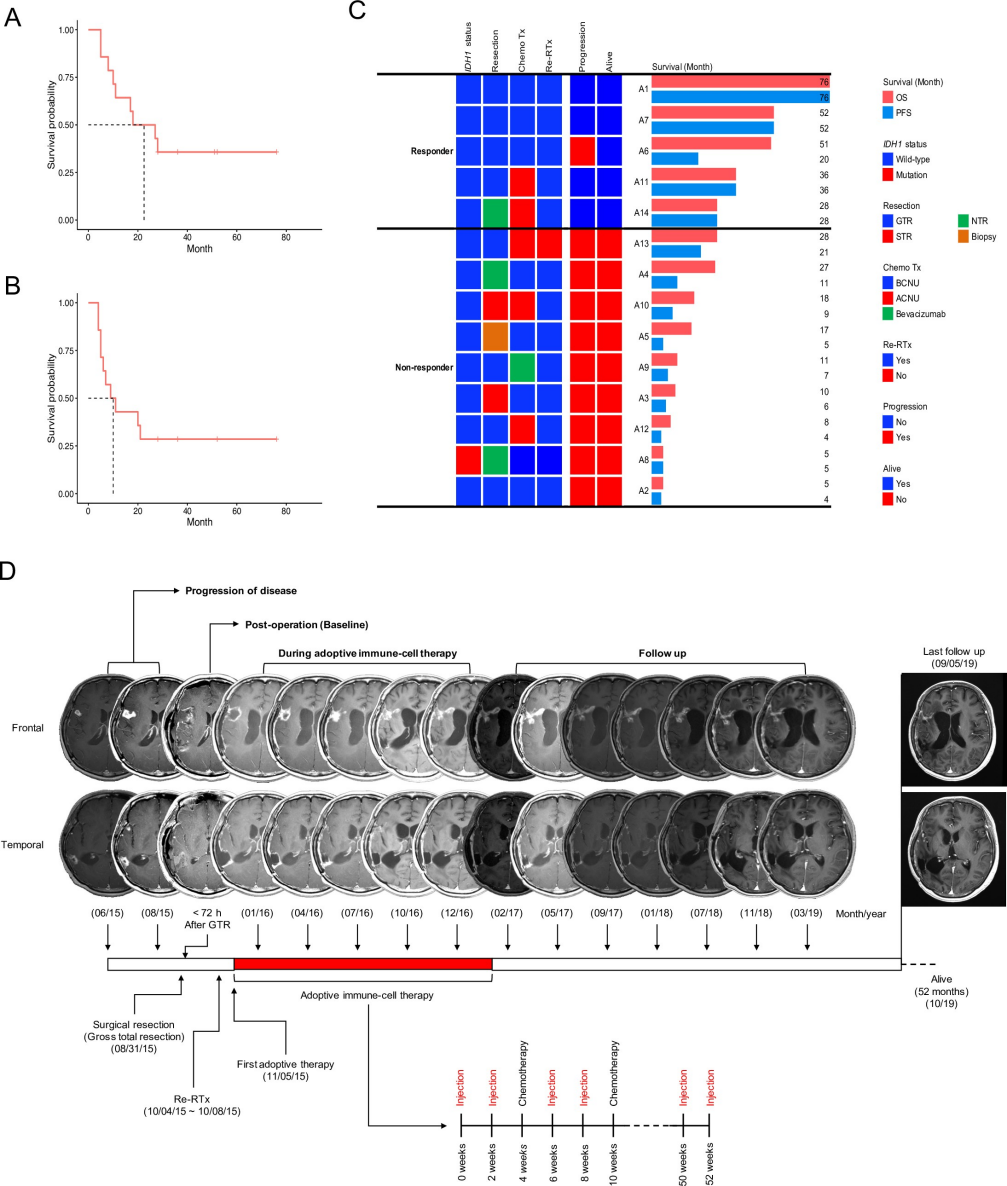
Kaplan-Meier plots. (A) overall survival (OS) and (B) progression free survival (PFS) of 14 patients with recurrent GBM who received the adoptive immune-cell therapy. (C) OS and PFS of good (n = 5) and poor responders (n = 9). (D) Therapeutic response assessment of one patient with recurrent GBM multiforme (A7) who received the adoptive immune-cell therapy with two different tumor lesions (frontal and temporal lobes) and showed good prognosis (overall survival: 46 months). Results of magnetic resonance imaging for evaluating the response to adoptive immune-cell therapy in the frontal and temporal lobes during injection of adoptive immune-cell and follow-up after completion. IDH1; isocitrate dehydrogenase, GTR; gross total resection, NTR; near-total resection, ChemoTx; chemotherapy, BCNU; carmustine, ACNU; nimustine, Re-RTx; re-irradiation therapy.

Of the 14 patients who received adoptive immune cell therapy, nine died during the study period. Five patients who had a durable response survived for at least 28 months and for a maximum of 76 months after tumor recurrence (Table 2 and S6 Fig in S1 File). There were no differences in terms of chemotherapy, re-irradiation, age-at-diagnosis of recurrent GBM, and sex between good and poor responders (Table 2). The median OS of adoptive-immune-cell-treated patients was 22.5 months (Fig 2A), and the median progression-free survival (PFS) was 10 months (Fig 2B). Five patients were alive for over 2 years and continued with activities of daily living. Four patients completed the adoptive immune-cell therapy (Fig 2C). One patient (A14) discontinued this therapy after the 11^th^ expanded cell injection owing to our inability to obtain enough expanded immune cells over four attempts (S2 Table in S1 File). Adoptive immune-cell therapy was discontinued in nine patients because of tumor progression (S2 Table in S1 File).

**Table 2:** Clinical information of good responders and poor responders.

	Good Responders	Poor responders
	(n = 5)	(n = 9)
**Age at recurrence event**		
Median (range)	55.2 (47–62)	52 (27–69)
**Sex**		
Male	2 (33.3%)	4 (66.7%)
Female	3 (37.5%)	5 (62.5%)
**Karnofsky performance status**		
100	0	0
90	1	0
80	2	1
70	2	3
60	0	5
**Chemotherapy**		
Carmustine (BCNU)	3	5
Nimustine (ACNU)	2	3
Bevacizumab	0	1
**Re-radiation therapy**		
Yes	5	8
No	0	1
**IDH1 mutation (R132) status**		
Wild type	5	8
Mutation	0	1
**Survival (above 2 years)**		
Alive	5	0
Dead	0	9
**Progression**		
No (%)	4	0
Yes (%)	1	9

The 2 years for OS and PFS rates of 14 patients who received adoptive immune-cell therapy were 50% (95% confidence interval [CI]: 30–84) and 29% (95% CI: 13–65), respectively. The OS and PFS of the historical cohort were 17% (95% CI: 9–31) and 6% (95% CI: 2–17), respectively (detailed information about historical measurement is in the supplementary data and S6 Table in S1 File).

### Evaluation of tumor response by MRI

Four patients (A1, A7, A11 and A14) with gross total resection (GTR) or near-total resection (NTR) presented long-lasting contrast enhancement (<6 months after completing the adoptive immune-cell therapy) without any clinical decline. The contrast enhancement had a Swiss cheese or soap bubble morphology, which was observed to increase gradually during therapy, followed by a time-dependent decrease after the therapy (S6–S8 Figs in S1 File and Fig 2). According to the iRANO criteria, four patients presented stable disease (SD) with no measurable tumor lesion due to GTR or NTR (S2 Table in S1 File). In three patients (A2, A12, and A13), thick enhancement or ventricular wall enhancement was observed in the first T1-enhanced MR image during adoptive immune-cell therapy. This could be considered as progressive disease (PD) without early measurable radiographic enhancement, considering pseudoprogression after the initial adoptive immune-cell therapy (S9 Fig and S2 Table in S1 File). One patient (A6) presented an atypical MRI pattern. During the therapy, a pattern of marginal fuzzy enhancement similar to that observed in the favorable response group was observed, and the thickness of the enhanced area decreased (S10 Fig in S1 File). However, a new pattern of enhancement was observed 3 months after the completion of the 21st adoptive immune-cell treatments. It was considered as PD with early radiographic enhancement considering pseudoprogression after the initial adoptive immune-cell therapy (S2 Table in S1 File). This lesion was confirmed progression by biopsy. This patient was alive for 51 months after the first recurrence following additional surgery. As mentioned earlier, one patient (A14) with good response was withdrawn from the study after the 12^th^ expanded immune-cell therapy session. MRI revealed a pattern of fuzzy enhancement with cystic changes during the adoptive immune-cell therapy (S7 Fig in S1 File). This patient was alive for 28 months following the first recurrence and maintained a good Karnofsky Performance score (KPS). Finally, A14 was considered to show SD without any clinical decline (S2 Table in S1 File). The demographics of tumor response assessment in all participants are presented in S12 Fig in S1 File.

### NanoString results revealed high immune reaction signatures within good responders

To identify the mechanism of action underlying good and poor responses to treatment, transcriptomic analysis was performed with tissues from 12 patients with recurrent GBM prior to adoptive immune-cell therapy. Among 750 analyzed genes, 85 genes were identified to be significantly associated with PFS by Cox regression analysis or by differentially expressed gene (DEG) analysis between good and poor responders. Most of the 85 significant genes were associated with a protective effect (hazard ratio < 1) for PFS and were upregulated in good responders (detailed information is provided in the Supplemental Appendix in S1 File) (S7 Table and S13 and S14 Figs in S1 File).

**Fig 3:**
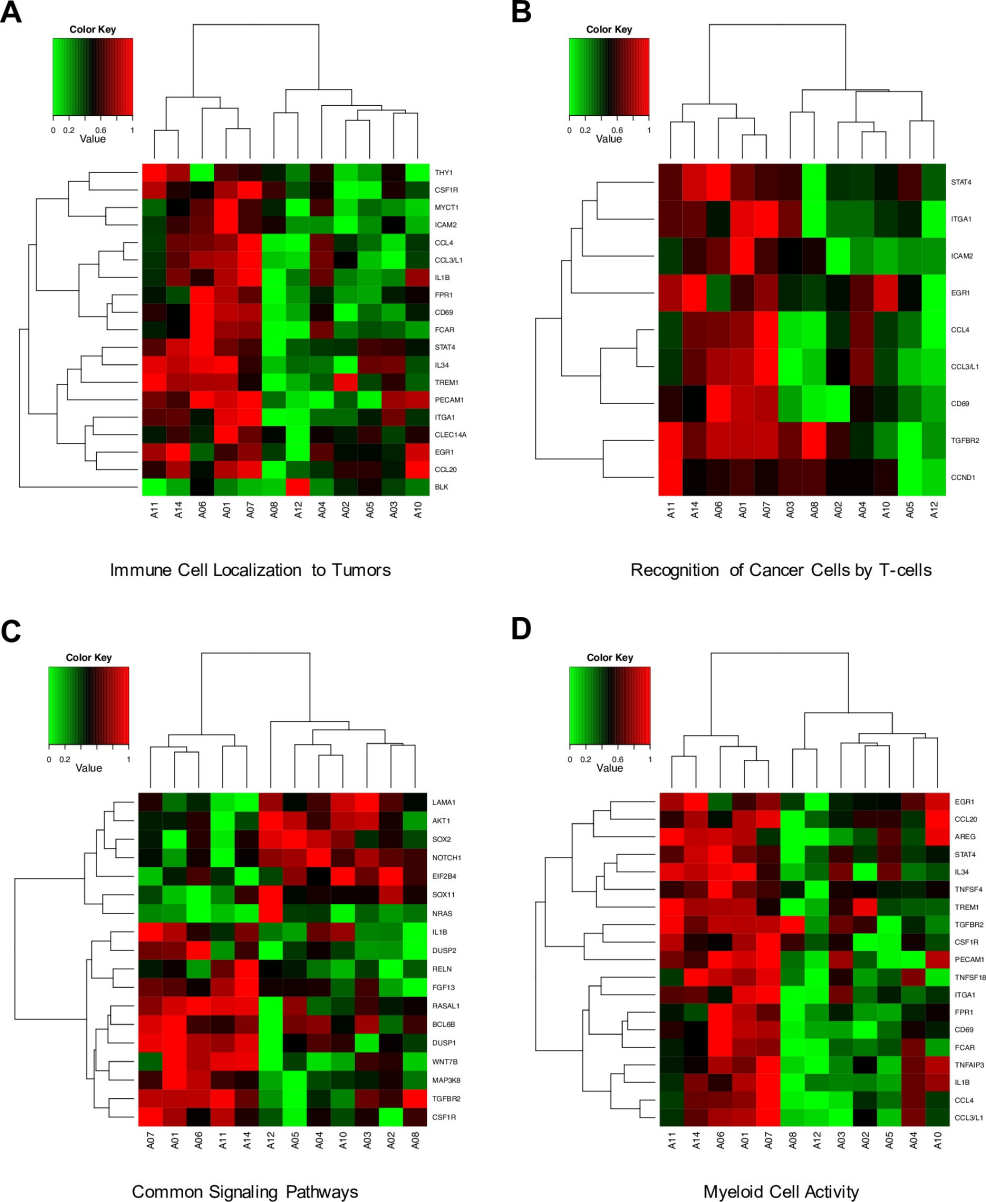
Cluster maps of four annotation terms (A-D) that fully discriminated between good (A1, A6, A7, A11, and A14) and poor responders. The expression levels of the genes were transformed into a 0–1 standard scale. (A) Immune cell localization to tumors, (B) recognition of cancer cells by T-cells, (C) common signaling pathways, (D) myeloid cell activity.

Forty-eight genes found to be significantly associated with PFS by Cox regression analysis were classified according to 12 major annotation terms in NanoString. Except for “NK Cells Activity”, all annotation terms included at least two significant genes (S8 Table in S1 File). Interestingly, genes in the four annotation terms including “Immune Cell Localization to Tumors”, “Recognition of Cancer Cells by T-cells”, “Myeloid Cell Activity”, and “Common Signaling Pathways” were fully discriminated between good responders and poor responders with an adjusted rand score of 1 (S8 Table in S1 File and Fig 3). In the annotated terms, “The Immune Cell Localization”, “Recognition of Cancer Cells by T-cells”, and “Myeloid Cell Activity”, except for B-cells marker (*BLK*), all genes significantly associated with PFS presented a protective effect. Genes linked to a significant protective effect were associated with chemokines (*CCL4*, *CCL3/L1*, and *CCL20*) and extravasation (*ICAM2*, *ITGA1*, and *PECAM1*) (Fig 3).

In addition, all significant genes that were associated with a protective effect for PFS, or that were up-regulated in good responders, were overexpressed in the mesenchymal sub-type in GBM patients of the Cancer Genome Atlas (TCGA) dataset (detailed information is provided in the Supplemental Appendix and S15 Fig in S1 File).

## Discussion

This study reports a phase I/IIa clinical trial on autologous, adoptive, expanded, and activated immune-cell therapy with high proportions of NK cells or T lymphocytes from PBMCs for patients with recurrent GBM. It adopted a single arm and open-labeled design to evaluate safety, efficacy, and mechanism of action of the adoptive immune-cell therapy.

The most important consideration in immune-cell treatment is immunological reaction with severe adverse effects. In this study, most of the patients experienced adverse effects of grades 1 and 2 related to the adoptive immune-cell therapy without any severe immunological rejection (Table 2 and S5 Table in S1 File), which manifests as a cytokine storm [15,16]. The most frequent events were mild immune-related symptoms, such as fever, hot flushes, chills, injection-site reaction, and gastrointestinal symptoms, such as anorexia, nausea, and constipation (Table 2). The hematological symptoms related to the immune-cells therapy were mild and reversed with conventional treatment. The most severe adverse effect related to this adoptive immune-cell therapy was a grade 3 fever, which resolved without any complication (Table 2 and S5 Table in S1 File). During adoptive immune-cell therapy, severe adverse events of grades 4 and 5 occurred 6 times. Leukocytopenia occurred 3 times, and thrombocytopenia occurred once. These events were related to the salvage chemotherapy as they occurred after administration of the chemo-drug, ACNU or BCNU, they were treated with medication and transfusion. Other severe adverse events occurred in the poor response group and were related to tumor progression. Hydrocephalus and severe brain swelling occurred in one case. Taken together, these results suggest that this adoptive immune-cell therapy is safe.

An anticancer therapeutic agent must prolong the patient’s survival and slow the progression of tumor. In the case of GBM, standard treatment using temozolomide and radiation extends the mean OS by approximately 4 months [1,17]. However, this is much lower than the OS observed for other cancers, and extension of OS is extremely rare in recurrent GBM. Recurrent GBM is associated with an OS of 6–8 months despite the administration of adjuvant treatment [4,18,19]. The median OS of adoptive immune-cell-treated patients was 22.5 months, and the median PFS was 10 months. Five patients survived for more than 2 years after the recurrence of GBM. Although this trial was conducted in a small number of patients (14), prolonged OS and PFS are important indicators of efficacy. These results indicate that adoptive immune-cell therapy could be used to treat GBM; however, they will have to be validated in clinical trials on a large number of patients.

Recently, clinical trials have been conducted using several immune cells or neoadjuvant immune check point inhibitors and have shown durable responses with OS of over 2 years in some recurrent GBM patients [8–11,20]. In this study, 5 patients showed a good response with OS of more than 2 years (Fig 2C). Four patients presented a durable response to the adoptive immune-cell therapy, without progression, and with a fuzzy pattern of enhancement or a “soap bubble or Swiss cheese appearance” with cystic changes on MRI (S6-S8 Figs in S1 File and Fig 2D). The fuzzy, enhanced lesions increased gradually during therapy, followed by a time-dependent decrease after the therapy. A thickened pattern of enhancement was observed for up to 14 months after the first adoptive immune-cell therapy session, after which the degree of enhancement decreased. This response was considered to indicate pseudoprogression with high immune reaction. Patients who did not respond to the adoptive immune-cell therapy presented a thick pattern of gadolinium enhancement or progression of ventricle wall enhancement (S9 and S11 Figs in S1 File). It is not clear whether the thickened pattern of enhancement was pseudoprogression or radiation necrosis [21,22]. Except A13 patient, all of the participants received re-irradiation therapy as salvage treatment before the administration of the adoptive immune-cell therapy (S2 Table in S1 File). These MRI signatures, which may be pseudoprogression or radiation necrosis, were continuously observed in good responders for at least 1 year. Compared to other modes of immunotherapy, immune cells were transferred many times (maximum of 24) to recurrent GBM patients in this study. Long-term-immune boosting effects were observed in the MRI signatures, indicating a durable immune effect and longer survival.

We performed NanoString transcriptomic analysis to elucidate the mechanism of action of the adoptive immune-cell therapy used in this study. Our results demonstrated that immune-related genes were associated with a protective effect in terms of PFS or were up-regulated in good responders (Fig 3, S13 and S14 Figs, and S7 Table in S1 File). In addition, four annotated terms including “Immune Cell Localization to Tumors”, “Recognition of Cancer Cells by T-cells”, “Myeloid Cell Activity”, and “Common Signaling Pathways” were able to discriminate between good and poor responders (Fig 3). Hyper-immunoactivity was detected in good responders. Recently, analysis of omics data was able to establish a subtype of GBM with high resolution [23–26]. The subtypes, classified based on multi-omics, were proneural, neural, classical, and mesenchymal. Several previous studies have reported that the mesenchymal subtype GBM is characterized by a higher degree of immune cell infiltration than the other subtypes, and it is considered more suitable to immunotherapy [27–30]. Using public GBM transcriptomic data from TCGA, all genes that were significantly associated with a protective effect for PFS or those that were upregulated in good responders, were found to be overexpressed in the mesenchymal hyper-enriched cluster (S15 Fig in S1 File). This indicates that this immune cell therapy may be efficacious in patients with an immune-response tumor type, such as those with mesenchymal subtype GBM from the TCGA dataset. A vaccination clinical trial using dendritic cells has also reported that the mesenchymal type GBM showed a good prognosis [31].

In the present study, *ex-vivo-*expanded and activated immune cells were administered by intravenous injection. Therefore, trafficking and transport of the immune cells to the tumor microenvironment are very important. Several previous studies revealed that the BBB is destroyed to different levels in patients with GBM, allowing immune cell trafficking [32,33]. In this study, using transcriptomic analysis, we elucidated the mechanism of action of this immune cell therapy and found a component involving the movement of immune cells to the tumor in order to maximize their tumor-killing ability. Several chemokines, including *CCL4*, *CCL3L1*, and *CCL20*, were upregulated in good responders or were associated with a protective effect for PFS. Several studies have reported that the *in vitro* activation of NK cells with IL-2 leads to the production of CCR2, CCR4, CCR5, and CCR8 [34–36]. Notably, these NK cells demonstrate high chemo-attractive responses with various chemokines including CCL4 [34,37–40]. Tumor tissues with increased level of these chemokines may have an enhanced ability to attract immune cells, which would move towards such tumor cells after administration. In the poor responders, although many immune cells were administered, the immune cells could not move toward the tumor microenvironment as the chemotactic signal was weak. Radiologically, an encouraging aspect about our results is pseudoprogression. The iRANO criteria suggest that pseudoprogression, in the MR scan after immunotherapy, could be considered a therapeutic response with immune infiltration [13]. As a result, in the MRI, long-lasting pseudoprogression was detected in only four good responders (S6-S8 Figs in S1 File and Fig 2D) with no additional tumor progression, but not in poor responders (S9 Fig in S1 File). These results suggest that immune cells administered to good responders would reach the tumor site via chemotaxis as a result of the immune response in the cancer microenvironment.

Consequently, in good responders with mesenchymal subtype, immune cells might have reached the brain. Immunohistochemical stain demonstrated that the CD3, CD8 or CD16 positive cells were significantly more infiltrated in tissue during adoptive immune cell therapy compared with pre-Tx in A1 patient with a good response (S1-S16 Figs and supplemental data in S1 File). Furthermore, by presenting long-lasting pseudoprogression, which is considered a treatment response to immunotherapy, good responders could present a long survival benefit. However, we were not able to experimentally determine whether NK cells actually exert anticancer effects among the expanded immune cell populations from PBMCs. In addition, as this was a clinical phase I/IIa trial on the safety and minimal efficacy, sufficient statistical power was not obtained due to the small number of patients. Therefore, further evaluation is needed through phase II clinical trials in the future.

## Conclusions

Autologous adoptive immune-cell therapy was safe and showed good clinical outcome. This therapy demonstrated durable responses in five patients without severe adverse effects, and transcriptomic analysis showed that the good responders had enhanced immune response signatures. Therefore, this adoptive immune-cell therapy may be effective in a group of recurrent GBM patients with enhanced immune signatures, such as those with mesenchymal subtype.

## Supporting information

S1 File Supplementary information. **Supplemental appendix**
○**Method of ex vivo expansion and in vitro evaluation of immune cells**○**Results and discussion of ex vivo expansion and in vitro evaluation of immune cells**○**Imaging analysis**○**Using the NanoString assay to assess the transcriptomic landscape to identify the mechanism of immune cells**○**Historical control group****S1–S8 Tables**○**S1 Table. Inclusion and exclusion criteria of KCT0003815**○**S2 Table. Patient treatments, status, and survival information of immune cell therapy group**○**S3 Table. Detailed relevant demographic of participants**○**S4 Table. Characterization of *ex-vivo-*expanded autologous immune cells from patients**○**S5 Table. Total adverse events during clinical trial of immune cell therapy**○**S6 Table. Patients treatments, status and survival information with historical control**○**S7 Table. Genes associated with progression free survival identified by Cox regression or DEG analysis of good and poor responders in the immune cell-treated group**○**S8 Table. Annotation-based unsupervised evaluation of label of clustering of genes significantly associated with PFS and label with good and poor responders****S1–S15 Figures**○**S1 Fig. Characterization of *ex-vivo*-expanded immune cells**. During immune cell expansion process, flow cytometry was performed for the cell populations, including NK cells (CD56+CD3–), T cells (CD56–CD3+), NKT cells (CD56+CD3+), B cells, or monocytes (CD56–CD3–) in PBMCs or immune cells; representative plots at 0, 6, 10, and 14 days (A). Representative plots for PBMCs (day 0) and immune cells (day 14) are shown for CD3/CD19 and CD14/side scattered light (SSC) cell populations (B). FACS analysis of specific T cells, marked with CD4/CD8 antibodies, is shown by gating T cells (CD3+) in PBMCs (day 0) or immune cells (day 14) (C). Represents the percentages of NK, T, and NKT cells, based on fluorescence-activated cell sorting (FACS) data and cell counting in PBMCs on 0, 6, 10, and 14 days (D). The percentages of cells in PBMCs (day 0) or immune cells (day 12) was calculated based on FACS data of figures B and C (E and G). The fold change of immune cell counts relative to PBMC counts was obtained from cell counting and cell population percentages (H). Percentages of viability of immune cells at 14 days (I).○**S2 Fig. Characterization of NK ligands in ex-vivo-expanded immune cells from seven healthy donors.** Representative flow cytometry histograms of activating and inhibitory NK cell receptors displayed for PBMCs (grey) or ex-vivo-expanded immune cells (white), gated as CD3–CD56+ (A). The positive percentages of NK cell receptors were measured in PBMCs (day 0) or ex-vivo-expanded immune cells (day 14) (B). Statistical comparisons are shown based on paired t-test; ns, no significance; **p ≤ 0.005, ***p ≤ 0.0005, ****p ≤ 0.00005○**S3 Fig. Characterization of intracellular cytotoxicity-related proteins in ex-vivo-expanded immune cells.** Intracellular cytotoxicity-related proteins and activation proteins were increased in ex-vivo-expanded immune cells, which are quantified by FACS analysis on day 0 and day 14 (A–C). Bar graphs represent % positive for the group of 7 healthy donors based on FACS analysis. ****p ≤ 0.00005 compared to PBMC group (day 0) analyzed by paired t-test.○**S4 Fig. Cytotoxicity assay of ex-vivo-expanded immune cells against K562 cell line.** When PBMCs (day 0) or immune cells (day 14) were co-cultured with K-562 cells, two-color flow cytometry data shows that ex-vivo-expanded immune cells showed higher levels of the CD56+CD107a+ cell population (A and B). The cytotoxicity assay confirmed that killing ability (K562 lysis) of immune cells (day 14) was dramatically increased compared to PBMCs (day 0) for the indicated effector: target (E: T) ratios (C). Quantification data represent mean ± SEM from 7 healthy donors. Statistical comparisons are shown; ** p ≤ 0.005, ****p ≤ 0.00005.○**S5 Fig. Cytotoxicity assay of ex-vivo-expanded immune cells against GBM cell lines.** NK cell-associated ligands were characterized in the four GBM cell lines. Isotype (grey) and antibody to each ligand (white) are showed in the histogram. The red and blue text in the histogram indicates the positive percentages and mean fluorescence intensity ratios, respectively (A). For the indicated E:T ratios, the cytolytic activity of PBMCs (day 0) or immune cells (day 14) towards the four GBM cell lines based on Figure A (B). Statistical comparisons are shown; * p ≤ 0.05, **p ≤ 0.005, ***p ≤ 0.0005, and ****p ≤ 0.00005.○**S6 Fig. Therapeutic response assessment of the first recurrent glioblastoma multiforme patient (A1) with the best prognosis (overall survival: 76 months) after adoptive immune-cell therapy.** Results of magnetic resonance imaging for evaluating the response to expanded immune-cell therapy during injection and follow-up after completion. Re-RTx, re-irradiation therapy.○**S7 Fig. MRI of A14 patients who did not receive treatment more than four times due to failure to sufficiently expand immune cells; the patient was withdrawn from the clinical study after the 12th immune cell injection.** The MRI showed fuzzy enhancement pattern with some cystic change. This patient has been alive for 22 months since the recurrence of GBM. ITx; immune cell therapy.○**S8 Fig. Therapeutic response assessment of recurrent GBM A11 patient who showed a good response to immune cell therapy (OS: 36 months).** Results of MRI for evaluating the response to immune cell therapy during injection of immune cells and follow-up after completion. Re-RTx; re-irradiation○**S9 Fig. T1-enhanced MRI after GTR or NTR of patients with poor prognosis.** Patients without durable response in GTR or NTR group showed a thick gadolinium enhancement pattern or progression along the ventricle wall enhancement. ITx; immune cell therapy.○**S10 Fig. MRI of A6 patient.** Until the final day of the immune cell treatment schedule, a marginal enhancement pattern was observed, and the thickness of enhancement area decreased. Newly thick enhancement was seen within 3 months after the completion of immune cell treatments. This lesion was confirmed as a progression by histopathology. ITx; immune cell therapy.○**S11 Fig. T1-enhanced MRI after STR (A) or biopsy (B) patients with poor progression.** Patients without durable response in STR or biopsy groups showed a strong and thick gadolinium enhancement pattern and progression along the ventricle wall enhancement. ITx; immune cell therapy.○**S12 Fig. Systemic demographic of radiographic tumor response assessment in participants.**○**S13 Fig. Proportions of regression risk (hazard ratio > 1) and protective (hazard ratio < 1) effects of genes significantly associated with PFS by Cox regression analysis (A) and proportions of upregulated and downregulated genes in good responders by t-test (|Fold-change| > 2) (B)**○**S14 Fig. Volcano plot of DEG analysis of good and poor responders.** Positive and negative fold change values indicate upregulation and downregulation in good responders, respectively. The 20 labeled genes indicate genes significantly associated with PFS in both the DEG analysis between good and poor responders and in the Cox regression analysis.○**S15 Fig. Transcriptomic landscape in GBM patients from the TCGA dataset with respect to genes identified by NanoString analysis to be significantly associated with PFS in immune cell-treated patients with recurrent GBM in this study.** Cluster map of TCGA GBM patients (A), pie plot to visualize proportions of each subtype based on gene expression clusters (B). The gene expression levels were transformed to a 0–1 standard scale.
(DOCX)

S2 File Clinical trial protocols. (PDF)

S3 File TREND checklist. (DOCX)

S1 Fig (TIF)
