# Supplementary material for: Autologous adoptive immune-cell therapy elicited a durable response with enhanced immune reaction signatures in patients with recurrent glioblastoma: An open label, phase I/IIa trial
Source: PLoS One. 2021 Mar 10;16(3):e0247293. doi: 10.1371/journal.pone.0247293 (PMC7946298; doi:10.1371/journal.pone.0247293)
Supplement: S1 File — Supplemental appendix ○Method of ex vivo expansion and in vitro evaluation of immune cells○Results and discussion of ex vivo expansion and in vitro evaluation of immune cells○Imaging analysis○Using the NanoString assay to assess the transcriptomic landscape to identify the mechanism of immune cells○Historical control groupS1–S8 Tables○S1 Table. Inclusion and exclusion criteria of KCT0003815○S2 Table. Patient treatments, status, and survival information of immune cell therapy group○S3 Table. Detailed relevant demographic of participants○S4 Table. Characterization of ex-vivo-expanded autologous immune cells from patients○S5 Table. Total adverse events during clinical trial of immune cell therapy○S6 Table. Patients treatments, status and survival information with historical control○S7 Table. Genes associated with progression free survival identified by Cox regression or DEG analysis of good and poor responders in the immune cell-treated group○S8 Table. Annotation-based unsupervised evaluation of label of clustering of genes significantly associated with PFS and label with good and poor respondersS1–S15 Figures○S1 Fig. Characterization of ex-vivo-expanded immune cells. During immune cell expansion process, flow cytometry was performed for the cell populations, including NK cells (CD56+CD3–), T cells (CD56–CD3+), NKT cells (CD56+CD3+), B cells, or monocytes (CD56–CD3–) in PBMCs or immune cells; representative plots at 0, 6, 10, and 14 days (A). Representative plots for PBMCs (day 0) and immune cells (day 14) are shown for CD3/CD19 and CD14/side scattered light (SSC) cell populations (B). FACS analysis of specific T cells, marked with CD4/CD8 antibodies, is shown by gating T cells (CD3+) in PBMCs (day 0) or immune cells (day 14) (C). Represents the percentages of NK, T, and NKT cells, based on fluorescence-activated cell sorting (FACS) data and cell counting in PBMCs on 0, 6, 10, and 14 days (D). The percentages of cells in PBMCs (day 0) or immune cells (day 12) was calcu [file pone.0247293.s001.docx]

**Supplementary information**

**Autologous adoptive immune-cell therapy elicited a durable response with enhanced immune reaction signatures in patients with recurrent glioblastoma: an open label, phase I/IIa trial**

Jaejoon Lim^1,¶^, YoungJoon Park^1,2,¶^, Ju Won Ahn^1,2^, JeongMin Sim^2^, Su Jung Kang^2^, Sojung Hwang^1,3^, Jin Chun^3^, Hyejeong Choi^4^, Sang Heum Kim^4^, Duk-Hee Chun^5^, Kyoung Su Sung^6^, KyuBum Kwack^2,*^, Kyunggi Cho^1,*^

^1^ Department of Neurosurgery, Bundang CHA Medical Center, CHA University College of Medicine

^2^ Department of Biomedical Science, College of Life Science, CHA University

^3^ Global Research Supporting Center, Bundang CHA Medical Center, CHA University College of Medicine

^4^ Department of Radiology, Bundang CHA Medical Center, CHA University College of Medicine

^5^ Department of Anesthesiology and Pain Medicine, Bundang CHA Medical Center, CHA University College of Medicine

^6^Department of Neurosurgery, Dong-A University Hospital, Dong-A University College of Medicine, Busan, Republic of Korea

^*^ Corresponding authors

e-mail: [sandori50@gmail.com](mailto:sandori50@gmail.com) (KC)

e-mail: [kbkwack@cha.ac.kr](mailto:kbkwack@cha.ac.kr) (KK)

^¶^These authors contributed equally to this work.

**Supplemental appendix**

1. Method of *ex vivo* expansion and *in vitro* evaluation of immune cells

***Ex-vivo* immune cell expansion**

Peripheral blood mononuclear cells (PBMCs) were isolated from whole blood of donors. The PBMCs were cultured in Alys505NK-EX serum-free medium (CSTI, Japan) containing 1000 IU/mL interleukin-2 (IL-2, Proleukin, Novartis, Switzerland), 50ng/mL IL-18 (R&D system, USA), 500ng/mL anti-NKp46 (R&D system, USA), 0.2 μg/mL gamma-globulin (Green Cross, Korea), and 5% autologous plasma. Fresh Alys505NK-EX serum-free medium was added at intervals of 1-3 days, transferred to Culture Bag A-1000NL (NIPRO, Japan) on the sixth day, and cultured for 14 days in the humidified environment of a CO_2_ incubator containing 5% CO_2_ at 37°C.

**Phenotype and cytotoxicity analyses of *ex-vivo*-expanded immune cells**

*Target cell culture*

K562, A172, U-373MG cell lines and T98G and U-87MG cell lines were cultured in RPMI 1640 (Thermo Fisher- Scientific, USA), while T98G and U-87MG cell lines were cultured in Dulbecco’s Modified Eagle Medium (Thermo Fisher Scientific, USA). Both media contained 10% fetal bovine serum (Thermo Fisher Scientific, USA) and 1% penicillin-streptomycin (Thermo Fisher Scientific, USA).

*Expression of cytotoxicity-related receptors and ligands*

To evaluate the phenotype and cytotoxicity of the expanded immune cells, protein levels of a total 22 natural killer (NK) cell cytotoxicity-related receptors and eight ligands in GBM cell lines were measured by CytoFLEX flow cytometry (Beckman Coulter, USA).

*Functional evaluation of expanded immune cell activity without target cells*

The production of three intracellular cytokines, including interferon-γ, perforin, and granzyme B was stimulated with phorbol 12-myristate 13-acetate (PMA) / ionomycin (BioLegend, USA) for 4 hours at 37°C in a 5% CO2 incubator. Only interferon- γ levels were measured by adding GolgiPlug (BD Bioscience, USA). Anti-interferon-γ-phycoerythrin (PE) (eBioscience, USA), anti-perforin-PE (eBioscience, USA), and anti-granzyme B-PE (eBioscience, USA) were stained with anti-CD56-allophycocyanin (APC) (eBioscience, USA). The three cytokines were analyzed by flow cytometry.

*Functional evaluation of expanded immune cell activity with K562*

PBMCs or expanded immune cells were co-cultured for 4 hours with K562 target cells with anti-CD107a-PE (eBioscience, USA), anti-CD3-fluorescein isothiocyanate (FITC) (eBioscience, USA), and anti-CD56-APC (eBioscience) (effector: target (E: T) = 5:1). After 4 h, CD107a levels in CD3–/CD56+ cells were measured by flow cytometry.

**Cytotoxicity assay**

PBMCs or expanded immune cells were co-incubated with K562 and four GBM cell lines, including T98G, U87MG, A172, and U-373MG, in various E: T ratios for 4 h. The K562 and four GBM cell lines were stained with 5μM carboxyfluorescein succinimidyl ester (Thermo Fisher Scientific, USA). After 4 h, dead cancer cells were analyzed with 7-aminoactinomycin D by flow cytometry.

**Statistical analysis**

Paired t-test was used to identify significant or differences of between day 0 and day 14 after *ex vivo* immune cell culture.

1. **Results and discussion of *ex vivo* expansion and *in vitro* evaluation of immune cells**

**Proportions of immune cells in expanded AKC**

PBMCs were isolated from whole blood of seven healthy donors. To manufacture immune cells, the isolated PBMCs were cultured with IL-2, IL-18, anti-NKp46 and gamma-globulin for 14 days. The percentages of CD3–/CD56+ cells in expanded immune cells were significantly increased at 14 days compared to day 0 (Fig S1A). On the other hand, proportions of CD3–/CD19+ cells, CD3+/CD19– cells, and CD3+/CD4+ cells, but not of CD3+/CD8+ cells and CD3+/CD56+ cells, were significantly reduced (Figs S1B-G). The proportion of other accessory cells, except for NK cells and CD8+ cells, was reduced in immune cells. At 14 days after culture, the number of NK cells increased about 1,259 times as compared to that before culture and increased to 95.7% of the total expanded PBMCs (Fig S1H).

**Protein levels of activating and inhibitory NK cells receptors**

As NK cells comprised a large proportion of the immune cells, the characteristics of NK cell-related receptors were observed. In order to identify the features of *ex-vivo*-expanded immune cells, we measured the protein level of NK cell receptors. Flow cytometry data are shown as histograms of the expression levels of NK cell receptors in PBMCs and *ex-vivo-*expanded immune cells (Fig S2A). The levels of NK cell activating receptors, including NKG2D, DNAM1, CD69, NKp30, NKp44, and CD2, were significantly increased in immune cells (Figs S2A and B). We did not observe any difference in the protein levels of NKp46, CD16, KIR2DL1, KIR2DL3, or KIR3DL1 (Figs S2A and B). Although the levels of the NKG2A, an inhibitory receptor, were increased, the increased in levels of the activating receptors was more noteworthy (Figs S2A and B). In other words, the balance of signaling between the inhibitory and activating receptors was tipped in favor of NK cell activation.

We were able to observe a dramatic increase in the levels of activating receptors. Protein such as perforin, Granzyme B, and IFN-γ that are involved in the activation and effector function of NK cells are produced as a result of their signal transduction cascade. Hence, intracellular levels of perforin, Granzyme B, and IFN-γ in PBMCs and immune cells were measured. When PBMCs or immune cells were activated with phorbol myristate acetate/ ionomycin, the numbers of NK cells producing these proteins greatly increased in immune cells compared to PBMCs (Figs S3A–C). This suggests that levels of perforin, Granzyme B, and IFN- γ were increased due to an upregulation of activating receptor expression.

**Cytotoxicity effect of immune cells towards K562 cells**

Degranulation and cell lytic activity of immune cells were assessed by co-culturing them with K-562 cells. After co-culture for 4 h, the levels of CD107a, a degranulation marker in NK cells, was found to be increased in immune cells compared to PBMCs (Figs S4A and B). It was also observed that the cytolytic activity of NK cells towards K-562 cells increased with each effector: target ratio (Fig S4C).

**Cytotoxicity of immune cells towards GBM cell lines**

Finally, the cytolytic activity of immune cells towards four GBM cell lines, T98G, U-87MG, A172, and U-373MG, was evaluated. Before evaluating cytotoxicity of immune cells, protein levels of ligands produced by GBM cell lines that bind to NK cell activating receptors and inhibitory receptors were determined (Fig S5A). After co-culture for each E:T ratio for 4 h, cytotoxicity against four GBM cell lines was found to be dramatically increased in immune cells as compared to PBMCs (Fig S5B). This suggests that the *in vitro* therapeutic potential of immune cell against GBM is mediated by the enhanced activation, expansion, and effector function of NK cells.

1. **Imaging analysis**

Magnetic resonance imaging (MRI) scans were obtained at screening (second preoperative), 24–72 h after the operation (baseline), and every three months. Brain MRI scans were performed to T1, T2, T2 Fluid-Attenuated Inversion Recovery (FLAIR), contrast enhanced T1, diffusion, and perfusion images. Tumors were evaluated within 7 days by two neuro-radiologists. Radiologic assessments were performed by an investigator blinded to study allocation or steroid dosage. Enhancing tumor measurements, T2/FLAIR change, and new lesions were evaluated in each MRI. Integration of clinical information and steroid dosage were discussed, and assessments were performed based on Response Assessment in Neuro-Oncology (RANO) in consensus. Patients who demonstrated progressive disease based on the RANO criteria, including the development of new lesions and confirmation of radiographic progression in follow-up imaging without clinically worsening, were followed up based on the immunotherapy Response Assessment in Neuro-Oncology (iRANO) criteria. Advanced MRI techniques (diffusion-weighted images and perfusion-weighted images) were performed for additional information in cases of suspected progression in follow-up MRI.

1. **Using the NanoString assay to assess the transcriptomic landscape to identify the mechanism of immune cells**

**Methods of pre-treatment transcriptomic analyses of tissues from adoptive immune cells treated patients**

To analyze NanoString data, Cox regression analysis was performed on 750 genes. We classified the genes found to be significantly associated with PFS (p-value < 0.05) according to 13 major annotation terms from The nCounter PanCancer IO 360 Panel: “Release of Cancer Cell Antigens”, “Cancer Antigen Presentation”, “T-cell Priming and Activation”, “Immune Cell Localization to Tumors”, “Stromal Factors”, “Recognition of Cancer Cells by T-cells”, “Killing of Cancer Cells”, “Myeloid Cell Activity”, “NK Cell Activity”, “Cell Cycle and Proliferation”, “Tumor-Intrinsic Factors”, “Immunometabolism” and “Common Signaling Pathways” (https://www.nanostring.com/download_file/view/1201/7354). These classified genes were clustered by using agglomerative clustering according to Ward’s method. Adjusted Rand scores were calculated between the clustered label and good (alive) and poor responder (death) for each annotation terms. Finally, we visualized the cluster maps for each annotation terms using Python (version: 3.6) and R (version: 3.5.0). To identify differentially expressed genes (DEGs) between good and poor responder, t-test was performed.

**Public GBM database**

Transcriptomic, non-silent somatic mutations and clinical data of GBM were downloaded from The Cancer Genome Atlas (TCGA) database using the Xena public data hubs (https://xenabrowser.net). A total of 147 GBM patients were selected.

**Hyper-immunoactivity phenotype in good responders**

Among 750 genes, 48 were found to be significantly associated with PFS. Of these 48 genes, 15 (31%) and 33 (69%) genes were associated with risk and protective effects for PFS, respectively (Fig S13A). A *t*-test was performed to identify DEGs showing significant differences between the two types of responders (p-value < 0.05 and |fold change| > 2). The expression levels of 57 genes were significantly different for the good and poor responders. Among these 57 genes, only three (5%) genes were upregulated in poor responders, and 54 (95%) genes were upregulated in good responders (Fig S13B). Among the 57 genes with significantly different expression levels between good and poor responders, 20 genes (labeled in Fig S14) were also found to be significantly associated with PFS (Fig S14).

**Expression of significantly upregulated genes in good responders or protective effects for PFS resembled a mesenchymal sub-type feature in GBM from TCGA dataset**

To examine the transcriptomic landscape in immune cell-treated patients with recurrent GBM using NanoString analysis, 85 genes that were significantly associated with PFS were identified by Cox-regression analysis for PFS or DEG analysis in good and poor responders. Agglomerative clustering was performed for these genes using the information from the GBM TGCA dataset. After clustering into two groups, DEG analysis was performed for the two groups, and genes were re-clustered into four groups. Significantly different genes from the two groups were identified by *t*-test with Bonferroni correction and |fold change| > 2.

As a result, the mesenchymal subtype was found to be dominantly enriched in cluster 1 (70%) (Fig S15). All genes that were upregulated in good responders or that had a protective effect for PFS were also overexpressed in cluster 1 (Fig S15). Conversely, all significantly upregulated genes in good responders were downregulated in cluster 2 (Fig S15A). The proneural and classical sub-types were dominantly enriched in cluster 2 (Fig S15B).

1. **Historical control group**

The historical control group comprised patients with recurrent GBM who were treated in our institution along with patients who were eligible to apply for this adoptive immune cell therapy clinical trial based on the same inclusion and exclusion criteria. In the database (IRB: sex, diagnosis, medical history, treatment history, medication, clinical feature, progression free survival, and survival) of patients with recurrent glioma treated in Bundang CHA Hospital since 2010, patients treated with the same strategy except the adoptive immune cell therapy were included in the historical control group.

1. **Immunohistochemical staining of pre- and during Tx in a good responder**

During adoptive immune cell therapy, we performed biopsy in A1 patient (good responder) to determine whether the radiographically enhanced site was progression or pseudoprogression after 4^th^ immunotherapy. As a result, it was confirmed as pseudoprogression. Using the biopsy tissue, we confirmed the immune cell infiltration in brain tissue during adoptive immune cell therapy in a good responder. We compared immune infiltration in tissues between pre-immunotherapy (GTR) and during immunotherapy (biopsy) by immunohistochemical staining. As a result, the number of positive cells for various immune cell markers, CD3, CD8 and CD16, was significantly more infiltrated (Fig S16). Immunohistochemical stain was evaluated according to the average frequency of positive cells after examining 5 randomly selected fields at x400 high-power magnification.

S1 Table. Inclusion and exclusion criteria of KCT0003815.

| **Inclusion criteria** | **Exclusion criteria** |
| --- | --- |
| 1. Age of 20 ~ 70 years. 2. Patients in whom recurrent glioblastoma was confirmed by MRI and in whom standard therapy had failed 3. Patients whose survival was expected to be at least 3 months. 4. Patients with appropriate bone marrow function:  - Hemoglobin ≥ 10 g/dL - White blood cells ≥ 3,000 units/mm^3^ - Absolute neutrophil ≥ 1,500 units/uL - Platelets ≥ 75,000 units/mm^3^  1. Patients with adequate liver and renal function:  - Total bilirubin ≤ 1.51 times the normal upper limit. - AST and ALT ≤ 2.5 times the normal upper limit. - Alkaline phosphatase ≤ 1.51 times the normal upper limit. - Serum creatinine ≤ the normal upper limit.  1. Patients who had been fully informed of the purpose, contents, and characteristics of immune cells, and who could give their signed consent or through their guardian or legal representative before the start of the clinical trial 2. Women who have been identified to not pregnant by the urine or blood pregnancy test 7 days prior to the start of the study, and who have agreed to use the appropriate method of contraception during the trial. 3. Patients without blood infection within 6 months of the trial. 4. Patients not on other immunotherapies. | 1. Patients with severe cardiopulmonary dysfunction (at the clinician’s discretion) 2. Patients with immunodeficiency or autoimmune diseases that can be exacerbated by immunotherapy (eg, rheumatoid arthritis, systemic lupus erythematosis, vasculitis, multiple sclerosis or adolescent-onset insulin-dependent diabetes mellitus) 3. Patients with chronic hepatitis B and hepatitis C carriers or HIV antibody-positive patients 4. Patients with a history of severe allergy 5. Uncontrolled hypertension or diabetic patients 6. Pregnant or lactating women 7. Patients who are considered to be unfit for clinical trials due to a severe medical or psychiatric illness 8. Patients with significant hemorrhagic disease not associated with cancer 9. Patients who participated in other clinical trials within 4 weeks of the start of this trial |

| **S2 Table. Patient treatments, status, and survival information of immune cell therapy group.** | | | | | | | | | | | | | | | | |
| --- | --- | --- | --- | --- | --- | --- | --- | --- | --- | --- | --- | --- | --- | --- | --- | --- |
| ID | sex | age | Prior rec # | Immune cell Tx # | OS rec | OS Tx | PFS rec | PFS Tx | Resection | Measurement of lesion (baseline) | iRANO criteria | PsP status during Tx | Chemo Tx with immune cell | Re-RTx | Post-op KPS | IDH1 status |
| A1 | F | 47 | 2 | 23* | 76+ | 68+ | 76+ | 68+ | GTR | Non-measurable | SD | Yes | BCNU | Yes | 70 | Negative |
| A2 | M | 52 | 2 | 4 | 5 | 3 | 4 | 2 | GTR | Non-measurable | PD | No | BCNU | Yes | 60 | Negative |
| A3 | F | 59 | 1 | 9 | 10 | 7 | 6 | 3 | STR | Non-measurable  (but present) | PD | No | BCNU | Yes | 60 | Negative |
| A4 | F | 27 | 2 | 13 | 27 | 23 | 11 | 8 | NTR | Non-measurable | PD | No | BCNU | Yes | 80 | Negative |
| A5 | F | 48 | 2 | 6 | 17 | 15 | 5 | 3 | biopsy | 14.13 mm × 13.97 mm | PD | No | BCNU | Yes | 70 | Negative |
| A6 | M | 57 | 2 | 21* | 51+ | 49+ | 20 | 18 | GTR | Non-measurable | PD | Yes | BCNU | Yes | 70 | Negative |
| A7 | F | 57 | 1 | 23* | 52+ | 47+ | 52+ | 47+ | GTR | Non-measurable | SD | Yes | BCNU | Yes | 80 | Negative |
| A8 | F | 51 | 1 | 5 | 5 | 4 | 5 | 4 | NTR | Non-measurable | PD | No | BCNU | Yes | 60 | Positive |
| A9 | M | 55 | 1 | 8 | 11 | 9 | 7 | 4 | GTR | Non-measurable | PD | No | Bevacizumab | Yes | 70 | Negative |
| A10 | M | 56 | 2 | 8 | 18 | 14 | 9 | 4 | STR | Non-measurable  (but present) | PD | No | ACNU | Yes | 60 | Negative |
| A11 | F | 53 | 2 | 23* | 36+ | 33+ | 36+ | 33+ | GTR | Non-measurable | SD | Yes | ACNU | Yes | 80 | Negative |
| A12 | F | 51 | 1 | 3 | 8 | 4 | 4 | 1 | GTR | Non-measurable | PD | No | ACNU | Yes | 60 | Negative |
| A13 | M | 69 | 1 | 4 | 28 | 9 | 21 | 2 | GTR | Non-measurable | PD | No | ACNU | No | 70 | Negative |
| A14 | M | 62 | 1 | 11 | 28+ | 21+ | 28+ | 21+ | NTR | Non-measurable | SD | Yes | ACNU | Yes | 90 | Negative |
| **Prior rec #;** Number of recurrence events, **Immune cell Tx #;** Number of injection of immune cell, ***;** Complete injection (in all patients without *, adoptive immune cell therapy was discontinued after progression), **OS rec;** Overall survival in months since recently recurrent event, **OS Tx;** Overall survival in months since first immune cell Tx, **PFS rec;** Progression-free survival in months since recently recurrent event, **PFS Tx;** Progression-free survival in months since first immune cell Tx, **+;** Alive, **GTR;** Gross total resection, **STR;** Subtotal resection, **NTR;** Near total resection, **Post-op KPS;** Post-operative Karnofsky Performance Scale, **Re-RTx**; Re-irradiation, **BCNU**; carmustine, **ACNU**; nimustine**, PsP;** pseudoprogression, SD; stable disease, PD; progressive disease | | | | | | | | | | | | | | | | |

| **S3 Table.** **Detailed relevant demographic of participants.** | | | | | | | | | | |
| --- | --- | --- | --- | --- | --- | --- | --- | --- | --- | --- |
| Patient | Age^a)^ | Hemoglobin count^b)^ | Leukocyte count^c)^ | Absolute neutrophil count^d)^ | Platelet count^e)^ | Total bilirubin^f)^ | AST^g)^ | ALT^h)^ | Alkaline phosphatase^i)^ | serum creatinine^j)^ |
| A1 | 47 | 11.6 | 3930 | 2975 | 245,000 | 0.53 | 11 | 13 | 13 | 0.8 |
| A2 | 52 | 11.9 | 7820 | 6099 | 170,000 | 0.75 | 22 | 38 | 210 | 0.6 |
| A3 | 59 | 12.3 | 6060 | 4728 | 235,000 | 0.45 | 19 | 27 | 266 | 0.5 |
| A4 | 27 | 10.8 | 6490 | 5367 | 133,000 | 0.39 | 10 | 5 | 124 | 0.7 |
| A5 | 48 | 12.9 | 3990 | 2633 | 163,000 | 0.48 | 11 | 11 | 154 | 0.7 |
| A6 | 57 | 14 | 6200 | 4147 | 254,000 | 0.55 | 21 | 22 | 235 | 1.2 |
| A7 | 57 | 12 | 6020 | 3473 | 296,000 | 0.23 | 17 | 13 | 199 | 0.8 |
| A8 | 51 | 11.2 | 3120 | 2115 | 121,000 | 0.42 | 15 | 12 | 60 | 0.7 |
| A9 | 55 | 14.6 | 4780 | 2949 | 159,000 | 0.31 | 21 | 34 | 68 | 0.9 |
| A10 | 56 | 11.5 | 4400 | 2596 | 307,000 | 0.33 | 22 | 32 | 44 | 0.7 |
| A11 | 53 | 10.5 | 4040 | 2908 | 175,000 | 0.29 | 24 | 23 | 65 | 0.5 |
| A12 | 51 | 12.7 | 6230 | 4037 | 252,000 | 0.4 | 15 | 9 | 60 | 0.5 |
| A13 | 69 | 13.2 | 4560 | 2841 | 185,000 | 0.6 | 25 | 34 | 93 | 0.5 |
| A14 | 62 | 13.6 | 7140 | 4169 | 203,000 | 0.46 | 19 | 14 | 78 | 1 |
| ^a)^ > 20 & < 70 ^b)^ > 10 g/dL ^c)^ > 3,000/mm3 ^d)^ > 1,500/ML ^e)^ > 75.000/mm3 ^f)^ < 1.5 times the normal upper limit (1.2 mg/dL) specified by the testing agency ^g)^ < 2.5 times the normal upper limit (40 IU/L) specified by the testing agency ^h)^ < 2.5 times the normal upper limit (40 IU/L) specified by the testing agency ^i)^ < 1.5 times the normal upper limit (250 IU/L) specified by the testing agency ^j)^ < Normal upper limit (1.2 mg/dL) specified by the testing agency | | | | | | | | | | |

| **S4 Table.** **Characterization of *ex-vivo-*expanded autologous immune cells from patients.** | | | | | | | | | | |
| --- | --- | --- | --- | --- | --- | --- | --- | --- | --- | --- |
| **Number** | **Product ID** | **Date of manufacture*** | **Patient ID** | **CD56+** | **CD3-/CD56+** | **CD3+/CD56+** | **CD3+CD56-** | **CD16+/CD56+** | **CD3-/CD19+** | **CD3-CD56+** |
|  |  |  |  |  |  |  |  |  |  | **gated CD16+** |
| 1 | AKC-0004 | 2.6.2014 | A1 | 90.98 | 87.63 | 3.35 | 2.49 | 89.80 | 0.56 | 98.90 |
| 2 | AKC-0005 | 2.6.2014 | A2 | 67.60 | 46.60 | 21.00 | 30.80 | 56.10 | 0.06 | 95.30 |
| 3 | AKC-0006 | 2.20.2014 | A1 | 68.00 | 52.90 | 15.10 | 24.40 | 62.70 | 1.12 | 94.90 |
| 4 | AKC-0007 | 2.20.2014 | A2 | 64.80 | 52.30 | 12.50 | 30.50 | 56.40 | 0.03 | 93.80 |
| 5 | AKC-0008 | 3.6.2014 | A1 | 60.10 | 48.10 | 12.00 | 27.90 | 53.80 | 1.25 | 95.30 |
| 6 | AKC-0010 | 4.3.2014 | A1 | 63.80 | 49.10 | 14.70 | 34.70 | 59.30 | 0.44 | 81.40 |
| 7 | AKC-0011 | 4.3.2014 | A2 | 61.90 | 44.60 | 17.30 | 37.10 | 48.20 | 0.22 | 93.60 |
| 8 | AKC-0012 | 4.17.2014 | A1 | 52.00 | 44.64 | 7.36 | 42.70 | 46.60 | 0.02 | 94.20 |
| 9 | AKC-0013 | 4.17.2014 | A2 | 61.65 | 57.48 | 4.17 | 15.90 | 57.00 | 0.44 | 87.50 |
| 10 | AKC-0014 | 5.15.2014 | A1 | 53.10 | 45.80 | 7.30 | 37.30 | 46.00 | 0.26 | 90.40 |
| 11 | AKC-0015 | 5.29.2014 | A1 | 39.53 | 32.52 | 7.01 | 46.50 | 37.60 | 0.31 | 97.40 |
| 12 | AKC-0016 | 6.26.2014 | A1 | 84.18 | 78.00 | 6.18 | 11.80 | 82.90 | 0.71 | 99.30 |
| 13 | AKC-0017 | 7.10.2014 | A1 | 89.60 | 81.20 | 8.40 | 6.91 | 87.30 | 0.41 | 98.40 |
| 14 | AKC-0018 | 8.7.2014 | A1 | 74.50 | 51.90 | 22.60 | 23.20 | 59.90 | 0.79 | 98.30 |
| 15 | AKC-0019 | 8.21.2014 | A1 | 68.60 | 50.40 | 18.20 | 29.00 | 59.70 | 0.25 | 98.80 |
| 16 | AKC-0020 | 9.18.2014 | A1 | 67.70 | 55.90 | 11.80 | 27.90 | 62.50 | 0.10 | 97.40 |
| 17 | AKC-0021 | 10.2.2014 | A1 | 84.60 | 77.31 | 7.29 | 13.80 | 82.10 | 0.20 | 99.20 |
| 18 | AKC-0022 | 10.16.2014 | A3 | 65.55 | 60.80 | 4.75 | 23.10 | 62.30 | 0.12 | 95.80 |
| 19 | AKC-0023 | 10.30.2014 | A1 | 61.77 | 58.52 | 3.25 | 8.31 | 48.60 | 0.14 | 75.10 |
| 20 | AKC-0024 | 10.30.2014 | A3 | 59.60 | 53.40 | 6.20 | 33.90 | 51.70 | 0.13 | 86.70 |
| 21 | AKC-0025 | 11.13.2014 | A1 | 63.00 | 54.70 | 8.30 | 33.30 | 58.80 | 0.09 | 97.00 |
| 22 | AKC-0026 | 11.13.2014 | A3 | 82.33 | 79.60 | 2.73 | 9.61 | 80.50 | 0.49 | 98.30 |
| 23 | AKC-0027 | 12.11.2014 | A1 | 78.40 | 73.70 | 4.70 | 13.90 | 75.80 | 0.18 | 98.60 |
| 24 | AKC-0028 | 12.11.2014 | A3 | 82.54 | 80.94 | 1.60 | 5.30 | 79.20 | 0.15 | 96.00 |
| 25 | AKC-0029 | 12.24.2014 | A1 | 73.09 | 71.30 | 1.79 | 6.18 | 67.20 | 0.15 | 88.80 |
| 26 | AKC-0030 | 12.24.2014 | A3 | 71.59 | 70.60 | 0.99 | 6.12 | 66.10 | 0.07 | 91.40 |
| 27 | AKC-0031 | 1.22.2015 | A1 | 56.60 | 45.80 | 10.80 | 40.20 | 51.50 | 0.06 | 97.30 |
| 28 | AKC-0032 | 1.22.2015 | A3 | 82.20 | 80.10 | 2.10 | 6.19 | 78.10 | 0.09 | 93.00 |
| 29 | AKC-0033 | 2.5.2015 | A1 | 80.68 | 79.70 | 0.98 | 5.97 | 78.30 | 0.13 | 98.80 |
| 30 | AKC-0034 | 2.5.2015 | A3 | 78.31 | 77.00 | 1.31 | 5.46 | 75.40 | 0.10 | 94.20 |
| 31 | K-P001 | 3.3.2015 | A1 | 78.02 | 72.80 | 5.22 | 18.60 | 75.90 | 0.34 | 98.50 |
| 32 | K-P002 | 3.3.2015 | A3 | 63.90 | 56.30 | 7.60 | 29.50 | 57.90 | 0.14 | 95.00 |
| 33 | K-P003 | 3.17.2015 | A1 | 81.09 | 74.90 | 6.19 | 15.90 | 76.60 | 0.12 | 96.00 |
| 34 | K-P004 | 3.17.2015 | A3 | 85.96 | 83.68 | 2.28 | 4.70 | 81.50 | 0.11 | 93.90 |
| 35 | K-P005 | 4.16.2015 | A1 | 88.78 | 83.70 | 5.08 | 9.07 | 86.90 | 0.46 | 99.00 |
| 36 | K-P007 | 4.30.2015 | A1 | 65.90 | 59.84 | 6.06 | 27.80 | 62.40 | 0.19 | 95.70 |
| 37 | K-P009 | 6.11.2015 | A4 | 80.60 | 76.10 | 4.50 | 15.10 | 77.20 | 0.09 | 97.40 |
| 38 | K-P010 | 6.25.2015 | A4 | 71.68 | 64.50 | 7.18 | 22.70 | 67.30 | 0.08 | 97.40 |
| 39 | K-P011 | 7.9.2015 | A4 | 75.00 | 68.00 | 7.00 | 20.70 | 70.50 | 0.03 | 96.80 |
| 40 | K-P012 | 8.6.2015 | A4 | 62.80 | 52.92 | 9.88 | 35.80 | 58.80 | 0.05 | 96.50 |
| 41 | K-P013 | 8.6.2015 | A5 | 86.70 | 75.40 | 11.30 | 11.40 | 80.70 | 0.08 | 95.60 |
| 42 | K-P014 | 8.20.2015 | A4 | 69.20 | 55.10 | 14.10 | 28.70 | 65.20 | 0.06 | 97.90 |
| 43 | K-P015 | 8.20.2015 | A5 | 91.90 | 82.40 | 9.50 | 6.95 | 86.40 | 0.11 | 96.30 |
| 44 | K-P016 | 9.3.2015 | A5 | 85.80 | 80.43 | 5.37 | 12.10 | 82.20 | 0.13 | 97.70 |
| 45 | K-P017 | 9.3.2015 | A6 | 60.60 | 53.70 | 6.90 | 30.80 | 56.60 | 0.11 | 94.30 |
| 46 | K-P018 | 9.17.2015 | A4 | 58.50 | 45.60 | 12.90 | 39.00 | 53.70 | 0.02 | 97.00 |
| 47 | K-P019 | 9.17.2015 | A6 | 87.20 | 76.90 | 10.30 | 10.50 | 83.40 | 0.06 | 96.90 |
| 48 | K-P020 | 10.1.2015 | A6 | 80.80 | 67.40 | 13.40 | 16.50 | 74.90 | 0.07 | 97.70 |
| 49 | K-P021 | 10.1.2015 | A5 | 88.50 | 80.31 | 8.19 | 9.60 | 85.10 | 0.09 | 97.50 |
| 50 | K-P022 | 10.1.2015 | A4 | 60.40 | 46.30 | 14.10 | 37.40 | 57.50 | 0.01 | 98.70 |
| 51 | K-P023 | 10.15.2015 | A5 | 81.30 | 70.00 | 11.30 | 15.60 | 75.70 | 0.09 | 96.20 |
| 52 | K-P024 | 10.29.2015 | A6 | 80.10 | 60.50 | 19.60 | 17.20 | 75.20 | 0.03 | 97.90 |
| 53 | K-P025 | 10.29.2015 | A4 | 54.90 | 40.80 | 14.10 | 41.70 | 50.70 | 0.09 | 98.00 |
| 54 | K-P026 | 11.5.2015 | A7 | 70.50 | 63.20 | 7.30 | 23.30 | 66.90 | 0.10 | 98.90 |
| 55 | K-P027 | 11.12.2015 | A5 | 76.40 | 56.90 | 19.50 | 22.10 | 68.00 | 0.04 | 95.80 |
| 56 | K-P028 | 11.12.2015 | A6 | 74.50 | 53.00 | 21.50 | 23.70 | 70.70 | 0.12 | 97.70 |
| 57 | K-P029 | 11.12.2015 | A4 | 54.40 | 41.20 | 13.20 | 42.50 | 51.40 | 0.10 | 96.60 |
| 58 | K-P030 | 11.19.2015 | A7 | 75.80 | 67.30 | 8.50 | 20.00 | 69.90 | 0.13 | 94.50 |
| 59 | K-P031 | 12.3.2015 | A7 | 71.40 | 61.20 | 10.20 | 26.90 | 66.80 | 0.09 | 98.80 |
| 60 | K-P032 | 12.10.2015 | A6 | 69.20 | 56.10 | 13.10 | 28.40 | 66.00 | 0.09 | 98.30 |
| 61 | K-P033 | 12.10.2015 | A4 | 72.70 | 46.60 | 26.10 | 25.40 | 69.80 | 0.17 | 99.20 |
| 62 | K-P034 | 12.24.2015 | A4 | 56.80 | 41.90 | 14.90 | 40.80 | 52.10 | 0.03 | 96.80 |
| 63 | K-P035 | 12.24.2015 | A6 | 70.30 | 38.00 | 32.30 | 28.20 | 64.40 | 0.05 | 97.80 |
| 64 | K-P036 | 12.31.2015 | A7 | 53.90 | 47.10 | 6.80 | 37.00 | 50.90 | 0.03 | 97.70 |
| 65 | K-P037 | 1.14.2016 | A7 | 69.30 | 54.73 | 14.57 | 24.58 | 66.00 | 0.03 | 98.81 |
| 66 | K-P038 | 1.21.2016 | A4 | 52.60 | 37.80 | 14.80 | 43.05 | 48.70 | 0.00 | 96.91 |
| 67 | K-P039 | 1.21.2016 | A6 | 68.90 | 37.70 | 31.20 | 27.95 | 63.20 | 0.00 | 98.45 |
| 68 | K-P040 | 2.4.2016 | A4 | 52.50 | 34.80 | 17.70 | 44.51 | 48.20 | 0.49 | 98.06 |
| 69 | K-P041 | 2.4.2016 | A6 | 65.70 | 34.67 | 31.03 | 31.37 | 60.60 | 0.25 | 97.66 |
| 71 | K-P044 | 2.25.2016 | A7 | 81.00 | 69.57 | 11.43 | 12.80 | 78.20 | 0.08 | 98.75 |
| 72 | K-P045 | 3.3.2016 | A6 | 44.10 | 17.92 | 26.18 | 52.18 | 41.90 | 0.01 | 97.77 |
| 74 | K-P048 | 3.17.2016 | A6 | 54.80 | 27.95 | 26.85 | 41.19 | 50.60 | 0.03 | 95.66 |
| 75 | K-P049 | 3.24.2016 | A7 | 65.70 | 56.33 | 9.37 | 21.43 | 63.40 | 0.04 | 98.09 |
| 77 | K-P051 | 4.7.2016 | A7 | 66.60 | 57.30 | 9.30 | 23.73 | 64.10 | 0.04 | 98.87 |
| 79 | K-P054 | 4.19.2016 | A6 | 42.80 | 19.32 | 23.48 | 52.75 | 39.10 | 1.07 | 97.77 |
| 80 | K-P055 | 4.26.2016 | A6 | 44.30 | 24.13 | 20.17 | 48.53 | 41.50 | 0.03 | 95.51 |
| 81 | K-P056 | 5.3.2016 | A7 | 68.60 | 52.30 | 16.30 | 29.01 | 65.20 | 0.30 | 98.18 |
| 83 | K-P058 | 5.19.2016 | A7 | 78.70 | 70.88 | 7.82 | 16.87 | 75.40 | 0.08 | 97.54 |
| 85 | K-P062 | 6.16.2016 | A7 | 66.90 | 54.77 | 12.13 | 29.67 | 63.90 | 0.06 | 98.89 |
| 86 | K-P063 | 6.30.2016 | A7 | 67.10 | 56.05 | 11.05 | 29.40 | 63.90 | 0.09 | 97.73 |
| 87 | K-P065 | 7.7.2016 | A6 | 30.20 | 14.14 | 16.06 | 64.36 | 24.70 | 0.00 | 96.79 |
| 88 | K-P066 | 7.21.2016 | A6 | 69.80 | 49.46 | 20.34 | 26.40 | 65.70 | 0.11 | 98.79 |
| 89 | K-P068 | 7.14.2016 | A7 | 68.80 | 58.64 | 10.16 | 28.57 | 65.50 | 0.11 | 97.06 |
| 90 | K-P069 | 8.4.2016 | A8 | 66.00 | 28.20 | 37.80 | 29.62 | 40.10 | 0.01 | 94.17 |
| 91 | K-P070 | 8.11.2016 | A7 | 58.90 | 46.34 | 12.56 | 38.05 | 55.40 | 0.05 | 98.34 |
| 92 | K-P071 | 8.18.2016 | A6 | 59.30 | 37.60 | 21.70 | 31.43 | 45.80 | 0.02 | 91.23 |
| 93 | K-P072 | 8.18.2016 | A8 | 69.60 | 29.57 | 40.03 | 29.54 | 51.70 | 0.01 | 96.83 |
| 94 | K-P073 | 9.1.2016 | A6 | 76.40 | 60.74 | 15.66 | 18.79 | 71.20 | 0.18 | 98.25 |
| 95 | K-P074 | 9.1.2016 | A8 | 78.50 | 29.00 | 49.50 | 19.50 | 60.90 | 0.26 | 97.88 |
| 96 | K-P075 | 9.8.2016 | A7 | 56.10 | 40.41 | 15.69 | 40.65 | 52.20 | 0.21 | 99.08 |
| 98 | K-P077 | 9.22.2016 | A7 | 63.70 | 51.27 | 12.43 | 31.92 | 60.10 | 0.02 | 98.16 |
| 99 | K-P079 | 9.27.2016 | A8 | 51.90 | 10.99 | 40.91 | 47.07 | 45.90 | 6.75 | 98.70 |
| 101 | K-P081 | 10.11.2016 | A6 | 82.40 | 62.44 | 19.96 | 14.26 | 76.20 | 0.26 | 97.98 |
| 102 | K-P082 | 10.11.2016 | A8 | 33.50 | 16.74 | 16.76 | 64.77 | 27.50 | 0.07 | 98.80 |
| 103 | K-P083 | 10.20.2016 | A7 | 54.90 | 38.96 | 15.94 | 36.84 | 47.80 | 0.03 | 96.67 |
| 105 | K-P085 | 11.3.2016 | A7 | 51.66 | 42.65 | 9.01 | 43.34 | 48.40 | 0.06 | 97.83 |
| 106 | K-P086 | 11.3.2016 | A9 | 62.80 | 45.24 | 17.56 | 27.28 | 49.60 | 0.47 | 94.84 |
| 107 | K-P087 | 11.10.2016 | A6 | 69.20 | 51.16 | 18.04 | 25.41 | 62.80 | 0.05 | 96.78 |
| 108 | K-P089 | 11.17.2016 | A9 | 84.81 | 74.55 | 10.26 | 10.18 | 78.70 | 0.37 | 98.87 |
| 109 | K-P090 | 11.24.2016 | A6 | 73.20 | 50.99 | 22.21 | 21.70 | 68.50 | 0.08 | 98.81 |
| 110 | K-P091 | 12.1.2016 | A7 | 71.69 | 60.91 | 10.78 | 24.71 | 68.70 | 0.01 | 99.03 |
| 111 | K-P092 | 12.1.2016 | A9 | 82.20 | 66.97 | 15.23 | 13.35 | 73.20 | 0.23 | 97.87 |
| 112 | K-P093 | 12.8.2016 | A6 | 66.40 | 39.15 | 27.25 | 28.44 | 60.80 | 0.07 | 99.04 |
| 113 | K-P094 | 12.15.2016 | A7 | 66.80 | 54.68 | 12.12 | 28.43 | 64.00 | 0.06 | 99.31 |
| 114 | K-P095 | 12.22.2016 | A10 | 73.00 | 45.06 | 27.94 | 26.50 | 61.20 | 0.03 | 98.73 |
| 115 | K-P096 | 12.29.2016 | A9 | 76.70 | 60.69 | 16.01 | 17.48 | 65.90 | 0.39 | 97.69 |
| 116 | K-P097 | 1.5.2017 | A10 | 68.60 | 39.40 | 29.20 | 30.37 | 56.00 | 0.02 | 99.34 |
| 117 | K-P098 | 1.5.2017 | A11 | 52.90 | 29.08 | 23.82 | 44.28 | 45.20 | 0.01 | 98.92 |
| 118 | K-P099 | 1.12.2017 | A7 | 58.68 | 47.96 | 10.72 | 36.76 | 55.60 | 0.03 | 98.87 |
| 119 | K-P100 | 1.12.2017 | A9 | 74.70 | 59.64 | 15.06 | 18.85 | 62.80 | 0.72 | 95.32 |
| 120 | K-P101 | 1.19.2017 | A10 | 69.10 | 43.60 | 25.50 | 29.87 | 57.50 | 0.00 | 99.03 |
| 121 | K-P102 | 1.19.2017 | A11 | 82.80 | 64.91 | 17.89 | 14.46 | 74.30 | 0.01 | 99.17 |
| 122 | K-P103 | 1.26.2017 | A7 | 65.41 | 54.98 | 10.43 | 29.93 | 63.00 | 0.00 | 99.45 |
| 123 | K-P105 | 2.2.2017 | A11 | 73.40 | 45.32 | 28.08 | 25.06 | 58.10 | 0.03 | 99.54 |
| 124 | K-P106 | 2.9.2017 | A7 | 73.67 | 65.00 | 8.67 | 20.93 | 71.70 | 0.02 | 99.56 |
| 125 | K-P107 | 2.9.2017 | A9 | 78.35 | 70.38 | 7.97 | 12.33 | 71.20 | 0.62 | 98.17 |
| 126 | K-P109 | 2.16.2017 | A10 | 67.30 | 39.58 | 27.72 | 31.75 | 55.40 | 0.26 | 99.24 |
| 127 | K-P110 | 2.16.2017 | A12 | 80.67 | 77.38 | 3.29 | 10.80 | 79.20 | 0.12 | 98.59 |
| 128 | K-P112 | 3.2.2017 | A10 | 71.00 | 48.00 | 23.00 | 26.77 | 62.00 | 0.17 | 99.08 |
| 129 | K-P114 | 3.2.2017 | A12 | 79.05 | 75.43 | 3.62 | 7.70 | 77.30 | 0.19 | 99.23 |
| 130 | K-P115 | 3.9.2017 | A9 | 78.07 | 72.64 | 5.43 | 15.29 | 71.10 | 0.03 | 97.59 |
| 131 | K-P116 | 3.16.2017 | A11 | 73.10 | 35.38 | 37.72 | 25.22 | 54.00 | 0.06 | 98.60 |
| 132 | K-P117 | 3.16.2017 | A12 | 82.99 | 78.36 | 4.63 | 7.46 | 82.30 | 0.04 | 99.47 |
| 133 | K-P118 | 3.23.2017 | A9 | 76.29 | 66.92 | 9.37 | 18.41 | 69.50 | 0.04 | 96.80 |
| 134 | K-P119 | 3.30.2017 | A10 | 79.20 | 59.18 | 20.02 | 18.82 | 72.60 | 0.00 | 99.45 |
| 135 | K-P120 | 3.30.2017 | A11 | 78.30 | 41.86 | 36.44 | 18.93 | 59.20 | 0.10 | 97.06 |
| 136 | K-P123 | 4.13.2017 | A11 | 58.60 | 37.37 | 21.23 | 36.38 | 48.70 | 0.09 | 97.13 |
| 137 | K-P124 | 4.27.2017 | A11 | 79.40 | 51.54 | 27.86 | 16.32 | 65.20 | 0.03 | 98.31 |
| 138 | K-P125 | 4.27.2017 | A10 | 63.00 | 22.05 | 40.95 | 35.52 | 46.20 | 0.07 | 97.36 |
| 139 | K-P126 | 5.11.2017 | A10 | 50.50 | 21.03 | 29.47 | 48.33 | 45.70 | 0.01 | 97.56 |
| 140 | K-P127 | 5.11.2017 | A11 | 77.80 | 46.10 | 31.70 | 21.30 | 65.70 | 0.06 | 98.79 |
| 141 | K-P129 | 5.25.2017 | A11 | 71.20 | 26.90 | 44.30 | 27.67 | 52.80 | 0.03 | 98.27 |
| 142 | K-P134 | 6.29.2017 | A11 | 66.10 | 22.42 | 43.68 | 33.04 | 46.40 | 0.04 | 98.83 |
| 143 | K-P137 | 7.27.2017 | A11 | 68.40 | 27.86 | 40.54 | 30.46 | 46.70 | 0.00 | 98.86 |
| 144 | K-P138 | 8.10.2017 | A11 | 69.90 | 24.89 | 45.01 | 29.34 | 47.40 | 0.00 | 98.84 |
| 145 | K-P139 | 8.24.2017 | A11 | 69.00 | 21.49 | 47.51 | 30.15 | 46.40 | 0.00 | 98.68 |
| 146 | K-P140 | 9.7.2017 | A11 | 67.70 | 25.43 | 42.27 | 32.13 | 45.70 | 0.01 | 98.63 |
| 147 | K-P141 | 9.21.2017 | A11 | 67.90 | 25.33 | 42.57 | 31.29 | 48.50 | 0.01 | 97.41 |
| 148 | K-P142 | 9.21.2017 | A13 | 91.68 | 87.96 | 3.72 | 5.03 | 87.00 | 0.09 | 95.67 |
| 149 | K-P143 | 10.12.2017 | A11 | 67.50 | 41.15 | 26.35 | 31.71 | 56.10 | 0.00 | 98.82 |
| 150 | K-P144 | 10.12.2017 | A13 | 87.26 | 83.07 | 4.19 | 7.89 | 83.10 | 0.20 | 97.43 |
| 151 | K-P145 | 10.26.2017 | A11 | 72.40 | 31.10 | 41.30 | 26.48 | 51.40 | 0.01 | 98.28 |
| 152 | K-P146 | 10.26.2017 | A13 | 90.19 | 85.72 | 4.47 | 5.62 | 84.60 | 0.13 | 95.54 |
| 153 | K-P147 | 11.9.2017 | A11 | 69.20 | 29.36 | 39.84 | 29.17 | 51.00 | 0.05 | 98.78 |
| 154 | K-P149 | 11.23.2017 | A11 | 88.00 | 78.87 | 9.13 | 9.11 | 84.50 | 0.05 | 97.78 |
| 155 | K-P150 | 11.23.2017 | A13 | 67.60 | 32.98 | 34.62 | 31.69 | 50.10 | 0.02 | 97.58 |
| 156 | K-P151 | 12.7.2017 | A11 | 66.60 | 23.96 | 42.64 | 33.06 | 45.10 | 0.02 | 97.68 |
| 157 | K-P153 | 12.21.2017 | A11 | 65.00 | 22.50 | 42.50 | 31.99 | 45.80 | 0.01 | 96.25 |
| 158 | KP-155 | 1.4.2017 | A11 | 57.20 | 16.47 | 40.73 | 40.81 | 45.60 | 0.00 | 97.07 |
| 159 | KP-156 | 1.18.2018 | A11 | 71.50 | 22.62 | 48.88 | 26.90 | 48.70 | 0.01 | 97.52 |
| 160 | KP-158 | 2.22.2018 | A14 | 50.42 | 40.05 | 10.37 | 43.08 | 45.20 | 0.32 | 90.07 |
| 161 | KP-159 | 3.8.2018 | A14 | 80.90 | 68.28 | 12.62 | 16.54 | 76.20 | 0.18 | 98.15 |
| 162 | KP-160 | 3.22.2018 | A14 | 87.80 | 78.34 | 9.46 | 10.11 | 84.30 | 0.33 | 98.16 |
| 163 | KP-161 | 4.12.2018 | A14 | 66.80 | 52.90 | 13.90 | 28.95 | 61.30 | 0.10 | 97.90 |
| 164 | KP-163 | 5.10.2018 | A14 | 66.59 | 55.32 | 11.27 | 28.60 | 60.00 | 0.07 | 98.46 |
| 165 | KP-165 | 6.7.2018 | A14 | 52.37 | 41.69 | 10.68 | 43.95 | 46.10 | 0.22 | 98.53 |
| 166 | KP-166 | 6.21.2018 | A14 | 72.36 | 62.07 | 10.29 | 23.44 | 66.20 | 0.01 | 98.03 |
| 167 | KP-169 | 8.2.2018 | A14 | 66.25 | 57.39 | 8.86 | 27.04 | 58.90 | 0.11 | 97.66 |
| 168 | KP-170 | 8.16.2018 | A14 | 58.75 | 48.30 | 10.45 | 36.37 | 53.30 | 0.16 | 95.79 |
| 169 | KP-171 | 8.30.2018 | A14 | 67.50 | 55.14 | 12.36 | 29.54 | 59.50 | 0.02 | 99.14 |
| 170 | KP-172 | 9.13.2018 | A14 | 65.63 | 54.70 | 10.93 | 30.43 | 59.80 | 0.06 | 98.52 |
| 171 | KP-174 | 10.11.2018 | A14 | 66.91 | 56.48 | 10.43 | 29.57 | 60.10 | 0.02 | 97.59 |
| 172 | KP-175 | 10.25.2018 | A14 | 67.28 | 56.86 | 10.42 | 27.93 | 59.30 | 0.03 | 93.98 |
| Date of manufacture*; Date of manufacture of immune cells AKC in GMP facility; all values indicate percentages | | | | | | | | | | |

| **S5 Table. Total adverse events during clinical trial of immune cell therapy.** | | | | | |
| --- | --- | --- | --- | --- | --- |
| **Adverse event** | **Grade 1** | **Grade 2** | **Grade 3** | **Grade 4** | **Grade 5** |
| **Blood and lymphatic system disorders** |  |  |  |  |  |
| Anemia | 2 | 4 | - | - | - |
| Febrile neutropenia | - | - | 1 | - | - |
| Leucopenia | 1 | - | - | - | - |
| Leukocytosis | 1 | - | - | - | - |
| Leukopenia | 1 | - | - | - | - |
| Neutropenia | - | 1 | - | - | - |
| Neutropenic fever | - | 1 | - | - | - |
| Pancytopenia | - | - | 1 | - | - |
| **Cardiac disorders** |  |  |  |  |  |
| Chest pain - cardiac | 1 | - | - | - | - |
| Tachycardia | 1 | - | - | - | - |
| **Eye disorders** |  |  |  |  |  |
| Anisocoria | 1 | - | - | - | - |
| Conjunctivitis | - | 1 | - | - | - |
| **Gastrointestinal disorders** |  |  |  |  |  |
| Abdominal discomfort | 2 | - | - | - | - |
| Aphagia | - | 1 | - | - | - |
| Aphthous stomatitis | 1 | - | - | - | - |
| Burning in abdomen | 1 | - | - | - | - |
| Constipation | 8 | 1 | - | - | - |
| Diarrhea | 2 | - | - | - | - |
| Dyspepsia | 3 | - | - | - | - |
| Gastric ulcer | - | 1 | - | - | - |
| Gastritis | - | 1 | - | - | - |
| Gastrointestinal disorder | 1 | - | - | - | - |
| Gastrointestinal pain | - | 1 | - | - | - |
| Hemorrhoids | - | 1 | - | - | - |
| Nausea | 3 | - | - | - | - |
| Vomiting | 4 | - | - | - | - |
| **General disorders and administration site conditions** |  |  |  |  |  |
| Chills | 1 | - | - | - | - |
| Edema generalized | 1 | - | - | - | - |
| Edema limbs | 1 | - | - | - | - |
| Fatigue | 1 | - | - | - | - |
| Fever | 6 | 1 | 1 | - | - |
| Flu like symptoms | 6 | - | - | - | - |
| Gait disturbance | - | 1 | - | - | - |
| General body pain | 1 | - | - | - | - |
| Injection site reaction | - | 1 | - | - | - |
| Injection site swelling | 1 | - | - | - | - |
| Localized edema | 1 | - | - | - | - |
| Malaise | 1 | - | - | - | - |
| Weakness | 1 | - | - | - | - |
| Weakness generalized | 2 | - | - | - | - |
| **Immune system disorders** |  |  |  |  |  |
| Autoimmune disorder | - | 1 | - | - | - |
| Back abscess | 1 | - | - | - | - |
| Brain abscess | - | - | 1 | - | - |
| Gastroenteritis | - | 1 | - | - | - |
| Herpes zoster | 1 | - | - | - | - |
| Lip infection | - | - | 1 | - | - |
| Skin infection | 2 | - | - | - | - |
| Tinea pedis | 1 | - | - | - | - |
| Urinary tract infection | - | - | 1 | - | - |
| Vaginitis | 1 | - | - | - | - |
| **Injury, poisoning and procedural complications** |  |  |  |  |  |
| Postoperative discharge | - | 1 | 1 | - | - |
| Postoperative pain | 2 | - | 1 | - | - |
| Wound complication | 2 | - | - | - | - |
| Wound discharge increased | - | - | 1 | - | - |
| Wound oozing | 1 | - | - | - | - |
| Dislocation of shoulder | 1 | - | - | - | - |
| **Investigations** |  |  |  |  |  |
| Alanine aminotransferase increase | 1 | 1 | - | - | - |
| ALT increased | 1 | 1 | - | - | - |
| Aspartate aminotransferase increase | 1 | 1 | - | - | - |
| AST increased | 1 | - | - | - | - |
| HBsAg | - | 1 | - | - | - |
| Helicobacter pylori antibody | 1 | - | - | - | - |
| Neutrophil count decreased | 1 | - | - | 3 | - |
| Platelet count decreased | 1 | - | 2 | 1 | - |
| Stool culture positive | 1 | - | - | - | - |
| WBC decreased | 6 | 1 | 1 | - | - |
| Weight loss | 1 | - | - | - | - |
| **Metabolism and nutrition disorders** |  |  |  |  |  |
| Anorexia | 3 | 1 | - | - | - |
| Hyperglycemia | 2 | - | - | - | - |
| Hypoalbuminemia | 2 | 1 | - | - | - |
| Hypokalemia | 3 | - | - | - | - |
| **Musculoskeletal and connective tissue disorders** |  |  |  |  |  |
| Arthralgia | 1 | - | - | - | - |
| Coxalgia | 1 | - | - | - | - |
| Flank pain | 1 | - | - | - | - |
| Knee pain | 2 | - | - | - | - |
| Leg pain | 3 | - | - | - | - |
| Low back pain | 1 | - | - | - | - |
| Lumbar pain | 1 | - | - | - | - |
| Muscle weakness | 1 | - | - | - | - |
| Muscle weakness aggravated | - | 1 | - | - | - |
| Muscle weakness NOS | - | 1 | - | - | - |
| Neck pain | 1 | - | - | - | - |
| Shoulder pain | 2 | - | - | - | - |
| **Nervous system disorders** |  |  |  |  |  |
| Accessory nerve disorder NOS | - | - | 1 | - | - |
| Amnesia | 1 | - | - | - | - |
| Brain swelling | - | - | 1 | 1 | - |
| Cerebral edema | - | 1 | 1 | - | - |
| Cognitive disorders | - | - | 1 | - | - |
| Depressed level of consciousness | 1 | - | - | - | - |
| Dizziness | 5 | - | - | - | - |
| Drowsy on awakening | - | 1 | - | - | - |
| Dysarthria | 1 | 1 | - | - | - |
| Head pain | - | 1 | - | - | - |
| Headache | 11 | 1 | - | - | - |
| Hydrocephalus | - | - | 1 | - | 1 |
| Hypersomnia | 1 | - | - | - | - |
| Hypoesthesia | - | 1 | - | - | - |
| Lethargy | 1 | - | - | - | - |
| Memory impairment | 2 | - | - | - | - |
| Movements involuntary | 1 | - | - | - | - |
| Neuropathic pain | 1 | - | - | - | - |
| Paresthesia | - | 2 | - | - | - |
| Seizure | - | 1 | 1 | - | - |
| Tremor limb | 1 | - | - | - | - |
| **Psychiatric disorders** |  |  |  |  |  |
| Agitation | 1 | - | - | - | - |
| Anxiety | 1 | - | - | - | - |
| Depression | 1 | 1 | - | - | - |
| Insomnia | 4 | - | - | - | - |
| **Renal and urinary disorders** |  |  |  |  |  |
| Cystitis noninfective | 1 | - | - | - | - |
| Frequency urinary | 1 | - | - | - | - |
| Polyuria | 1 | - | - | - | - |
| Pyuria | 1 | - | - | - | - |
| Urination abnormal NOS | 1 | - | - | - | - |
| **Reproductive system and breast disorders** |  |  |  |  |  |
| Pelvic pain | 2 | - | - | - | - |
| **Respiratory, thoracic and mediastinal disorders** |  |  |  |  |  |
| Cough | 4 | - | - | - | - |
| Dyspnea | - | 1 | - | - | - |
| Nasal congestion | 1 | - | - | - | - |
| Productive cough | 2 | - | - | - | - |
| Rhinorrhea | 1 | - | - | - | - |
| Sputum | 2 | - | - | - | - |
| **Skin and subcutaneous tissue disorders** |  |  |  |  |  |
| Allergic rash | 1 | - | - | - | - |
| Bullous dermatitis | 1 | - | - | - | - |
| Itching | 2 | - | - | - | - |
| Pruritis | 1 | - | - | - | - |
| Rash face | 1 | - | - | - | - |
| Redness of legs | 1 | - | - | - | - |
| Skin cysts | 1 | - | - | - | - |
| Skin rash | 1 | - | - | - | - |
| Vesicle | 1 | - | - | - | - |
| **Vascular disorders** |  |  |  |  |  |
| Hot flushes | 1 | - | - | - | - |
| Hypertension | - | 1 | 1 | - | - |
| Hypotension | - | - | 1 | - | - |
| **Total** | 168 | 40 | 20 | 5 | 1 |
|  |  |  |  |  |  |

| **S6 Table. Patients treatments, status and survival information with historical control.** | | | | | | | | | |
| --- | --- | --- | --- | --- | --- | --- | --- | --- | --- |
| ID | Sex | Age | IDH1 status | Chemo Tx | Postop KPS | Re-RTx | OS | PFS | Resection |
| C1 | M | 38 | wild type | Temozolomide | 70 | Yes | 7 | 4 | NTR |
| C2 | M | 63 | wild type | None | 60 | No | 5 | 4 | STR |
| C3 | M | 39 | wild type | BCNU | 70 | Yes | 10 | 6 | STR |
| C4 | M | 56 | wild type | Temozolomide | 60 | Yes | 9 | 4 | GTR |
| C5 | M | 42 | wild type | BCNU | 80 | Yes | 10 | 5 | GTR |
| C6 | M | 55 | wild type | BCNU | 60 | No | 5 | 4 | STR |
| C7 | F | 52 | wild type | None | 60 | No | 3 | 3 | STR |
| C8 | M | 36 | wild type | None | 80 | No | 7 | 0 | GTR |
| C9 | M | 40 | wild type | Temozolomide | 80 | No | 8 | 3 | STR |
| C10 | M | 50 | wild type | Tamoxifen | 80 | Yes | 25 | 13 | STR |
| C11 | F | 41 | wild type | Temozolomide | 60 | Yes | 10 | 3 | NTR |
| C12 | F | 47 | mutation | None | 80 | Yes | 13 | 4 | STR |
| C13 | M | 65 | wild type | BCNU | 80 | Yes | 27 | 13 | GTR |
| C14 | M | 70 | mutation | Temozolomide | 70 | Yes | 19 | 2 | NTR |
| C15 | F | 32 | mutation | Temozolomide | 80 | Yes | 20 | 8 | NTR |
| C16 | M | 25 | wild type | Temozolomide | 60 | No | 6 | 2 | STR |
| C17 | M | 41 | wild type | PCV | 70 | Yes | 12 | 6 | GTR |
| C18 | M | 43 | wild type | Temozolomide | 80 | Yes | 28 | 9 | STR |
| C19 | M | 36 | wild type | Temozolomide | 90 | Yes | 9 | 3 | STR |
| C20 | M | 65 | wild type | None | 90 | Yes | 6 | 3 | STR |
| C21 | M | 49 | mutation | Tamoxifen | 90 | Yes | 34 | 9 | STR |
| C22 | F | 57 | wild type | BCNU | 90 | Yes | 8 | 1 | NTR |
| C23 | F | 42 | wild type | Temozolomide | 60 | No | 8 | 6 | GTR |
| C24 | M | 65 | wild type | Temozolomide | 80 | Yes | 20 | 5 | GTR |
| C25 | M | 42 | wild type | BCNU | 90 | No | 59+ | 59+ | GTR |
| C26 | M | 53 | wild type | None | 70 | Yes | 10 | 4 | GTR |
| C27 | M | 49 | wild type | BCNU | 80 | Yes | 8 | 5 | NTR |
| C28 | M | 37 | mutation | Temozolomide | 80 | Yes | 11 | 8 | NTR |
| C29 | F | 54 | wild type | Temozolomide | 90 | No | 70+ | 9 | GTR |
| C30 | F | 52 | wild type | BCNU | 80 | Yes | 51 | 13 | NTR |
| C31 | M | 26 | wild type | None | 60 | Yes | 7 | 3 | NTR |
| C32 | F | 54 | wild type | Tamoxifen | 70 | No | 18 | 12 | STR |
| C33 | F | 67 | wild type | None | 70 | No | 4 | 3 | NTR |
| C34 | F | 54 | wild type | BCNU | 60 | No | 11 | 7 | NTR |
| C35 | F | 58 | wild type | Temozolomide | 80 | Yes | 9 | 6 | STR |
| C36 | M | 65 | wild type | PCV | 90 | Yes | 9 | 8 | GTR |
| C37 | F | 60 | wild type | Tamoxifen | 80 | Yes | 12 | 10 | NTR |
| C38 | F | 66 | wild type | Temozolomide | 100 | Yes | 4 | 3 | STR |
| C39 | M | 55 | wild type | None | 80 | No | 8 | 3 | NTR |
| C40 | M | 47 | wild type | None | 80 | Yes | 15 | 10 | NTR |
| C41 | F | 56 | wild type | Temozolomide | 80 | Yes | 13 | 6 | STR |
| C42 | F | 60 | wild type | Tamoxifen | 70 | No | 19 | 3 | GTR |
| C43 | M | 23 | wild type | None | 70 | No | 12 | 8 | GTR |
| C44 | F | 50 | mutation | Temozolomide | 100 | Yes | 15 | 6 | STR |
| C45 | M | 35 | mutation | ACNU and Avastin | 80 | No | 61+ | 34 | NTR |
| C46 | M | 63 | wild type | Avastin | 60 | No | 9 | 5 | NTR |
| C47 | F | 58 | wild type | Temozolomide | 80 | No | 7 | 5 | STR |
| C48 | M | 38 | wild type | Avastin | 70 | Yes | 47+ | 47+ | STR |
| C49 | F | 42 | wild type | ACNU | 80 | Yes | 11 | 1 | GTR |
| C50 | F | 66 | wild type | Tamoxifen | 60 | No | 6 | 3 | STR |
| C51 | F | 68 | wild type | Tamoxifen | 70 | No | 9 | 7 | NTR |
| C52 | M | 54 | wild type | Avastin | 80 | No | 5 | 4 | STR |
| C53 | M | 67 | wild type | None | 90 | No | 3 | 3 | STR |
| **GTR**, gross total resection; **STR**, subtotal resection; **NTR**, near total resection; **Postop KPS**; postoperative Karnofsky performance scale, **Re-RTx**; re-radiotherapy, **+**; event did not occur | | | | | | | | | |

| **S7 Table. Genes associated with progression free survival identified by Cox regression or DEG analysis of good and poor responders in the immune cell-treated group.** | | | | | | |
| --- | --- | --- | --- | --- | --- | --- |
| **Gene** | Cox-regression | | | | t-test | |
|  | **Hazard ratio** | **95% CI* lower** | **95% CI* upper** | **P-value** | **Log2(FC)*** | **P-value** |
| *DUSP1* | 0.0139 | 0.0007 | 0.2821 | 0.0054 | 1.7439 | 0.0015 |
| *CSF1R* | 0.0063 | 0.0002 | 0.2231 | 0.0054 | 0.8523 | 0.0145 |
| *ITGA1* | 0.0085 | 0.0003 | 0.265 | 0.0066 | 0.8483 | 0.0099 |
| *FGF13* | 0.0147 | 0.0006 | 0.3498 | 0.0091 | 2.0272 | 0.0179 |
| *BLK* | 75.6994 | 2.7135 | 2111.7994 | 0.0108 | -1.3898 | 0.1829 |
| *CD69* | 0.0037 | 0 | 0.281 | 0.0112 | 2.0202 | 0.0015 |
| *BCL6B* | 0.008 | 0.0002 | 0.3589 | 0.0129 | 0.4877 | 0.1314 |
| *MSH6* | 92.339 | 2.5713 | 3316.037 | 0.0133 | -0.2669 | 0.0943 |
| *PDGFB* | 0.006 | 0.0001 | 0.3444 | 0.0133 | 0.8015 | 0.0303 |
| *CCL4* | 0.0151 | 0.0005 | 0.4229 | 0.0136 | 1.9846 | 0.0097 |
| *RASAL1* | 0.0281 | 0.0016 | 0.4848 | 0.014 | 1.7118 | 0.0022 |
| *IL1B* | 0.0152 | 0.0005 | 0.4358 | 0.0145 | 2.0572 | 0.0416 |
| *TNFSF18* | 0.0434 | 0.0035 | 0.5441 | 0.015 | 1.4257 | 0.0081 |
| *IL34* | 0.0303 | 0.0018 | 0.5199 | 0.0159 | 1.401 | 0.0036 |
| *BRD3* | 24.1742 | 1.7444 | 335.0151 | 0.0176 | -0.348 | 0.0088 |
| *EGR1* | 0.0218 | 0.0009 | 0.5127 | 0.0176 | 0.6403 | 0.1804 |
| *MAP3K8* | 0.0138 | 0.0004 | 0.5108 | 0.0201 | 1.0266 | 0.0036 |
| *CLEC14A* | 0.0045 | 0 | 0.4291 | 0.0201 | 0.4311 | 0.1197 |
| *CCL3/L1* | 0.029 | 0.0014 | 0.5852 | 0.0209 | 1.8839 | 0.0094 |
| *EIF2B4* | 34.7998 | 1.6358 | 740.3135 | 0.0229 | -0.2014 | 0.0234 |
| *TNFSF4* | 0.0072 | 0.0001 | 0.5144 | 0.0235 | 0.5557 | 0.0147 |
| *HELLS* | 41.2045 | 1.5991 | 1061.6963 | 0.0249 | -0.913 | 0.0256 |
| *SOX11* | 101.6605 | 1.747 | 5915.7527 | 0.0258 | -1.3749 | 0.0141 |
| *FCAR* | 0.0244 | 0.0009 | 0.6658 | 0.0277 | 2.0237 | 0.0098 |
| *ICAM2* | 0.0127 | 0.0003 | 0.6314 | 0.0285 | 0.8115 | 0.0073 |
| *MYCT1* | 0.0176 | 0.0005 | 0.6714 | 0.0297 | 0.8015 | 0.0242 |
| *NOTCH1* | 32.4794 | 1.4078 | 749.3219 | 0.0297 | -0.8081 | 0.0031 |
| *NRAS* | 32.8448 | 1.3679 | 788.6445 | 0.0313 | -0.3295 | 0.1138 |
| *LAMA1* | 25.1316 | 1.3162 | 479.8608 | 0.0321 | -1.4133 | 0.0062 |
| *STAT4* | 0.0408 | 0.0022 | 0.7688 | 0.0327 | 1.6404 | 0.0077 |
| *AREG* | 0.0715 | 0.0062 | 0.8271 | 0.0347 | 1.6497 | 0.0327 |
| *TNFAIP3* | 0.0293 | 0.0011 | 0.7803 | 0.035 | 1.1665 | 0.065 |
| *PECAM1* | 0.1317 | 0.0198 | 0.8775 | 0.0362 | 0.7393 | 0.0151 |
| *AKT1* | 73.1329 | 1.2957 | 4127.8976 | 0.037 | -0.3896 | 0.0421 |
| *CCND1* | 0.03 | 0.0011 | 0.8116 | 0.0372 | 0.6961 | 0.138 |
| *THY1* | 0.0963 | 0.0103 | 0.8971 | 0.0398 | 0.6431 | 0.1047 |
| *POLD1* | 32.3866 | 1.1531 | 909.6495 | 0.041 | -0.5745 | 0.1323 |
| *TREM1* | 0.0521 | 0.0031 | 0.8873 | 0.0411 | 1.272 | 0.0202 |
| *FANCA* | 31.4417 | 1.1339 | 871.8609 | 0.0419 | -0.7702 | 0.0513 |
| *CCL20* | 0.0542 | 0.0033 | 0.9017 | 0.0421 | 0.8565 | 0.1293 |
| *TGFBR2* | 0.0788 | 0.0067 | 0.9294 | 0.0436 | 0.696 | 0.0307 |
| *MELK* | 29.3856 | 1.0724 | 805.2111 | 0.0454 | -0.6725 | 0.2225 |
| *CCNB1* | 37.5531 | 1.0587 | 1332.0396 | 0.0464 | -1.3398 | 0.0333 |
| *DUSP2* | 0.0483 | 0.0024 | 0.9647 | 0.0473 | 1.7908 | 0.0145 |
| *RELN* | 0.0151 | 0.0002 | 0.9675 | 0.0482 | 0.9344 | 0.0647 |
| *SOX2* | 21.5481 | 1.0101 | 459.6893 | 0.0493 | -0.595 | 0.0375 |
| *FPR1* | 0.0287 | 0.0008 | 0.9927 | 0.0495 | 1.1752 | 0.0146 |
| *WNT7B* | 0.1019 | 0.0104 | 0.9998 | 0.05 | 1.4401 | 0.006 |
| *SGK1* | 0.1495 | 0.0068 | 3.2629 | 0.227 | 1.0053 | 0.0337 |
| *FCGR2A* | 0.1023 | 0.0034 | 3.052 | 0.1882 | 1.01 | 0.0239 |
| *TLR2* | 0.1718 | 0.0108 | 2.7212 | 0.2114 | 1.0124 | 0.0303 |
| *CD14* | 0.1863 | 0.0117 | 2.9754 | 0.2346 | 1.0147 | 0.0412 |
| *CLEC7A* | 0.0733 | 0.004 | 1.3434 | 0.0782 | 1.0493 | 0.0153 |
| *MS4A4A* | 0.1671 | 0.0076 | 3.6667 | 0.2562 | 1.1168 | 0.037 |
| *HLA-DPB1* | 0.0882 | 0.0036 | 2.1584 | 0.1367 | 1.1174 | 0.0171 |
| *CD45RO* | 0.1725 | 0.0099 | 3.0135 | 0.2285 | 1.1407 | 0.0147 |
| *IL10RA* | 0.0499 | 0.0012 | 2.1231 | 0.1172 | 1.1446 | 0.0169 |
| *PTPRC* | 0.2044 | 0.0068 | 6.1402 | 0.3605 | 1.1448 | 0.0405 |
| *TNFRSF1B* | 0.0442 | 0.001 | 1.8705 | 0.1026 | 1.192 | 0.0155 |
| *HLA-DRA* | 0.0591 | 0.0018 | 1.9333 | 0.112 | 1.1937 | 0.011 |
| *CMKLR1* | 0.1329 | 0.0041 | 4.2595 | 0.254 | 1.1965 | 0.0364 |
| *HLA-DPA1* | 0.063 | 0.0021 | 1.8756 | 0.1103 | 1.2092 | 0.0087 |
| *IL10* | 0.1619 | 0.0063 | 4.1696 | 0.2719 | 1.2504 | 0.0189 |
| *SNCA* | 0.229 | 0.0286 | 1.8316 | 0.1647 | 1.2572 | 0.0178 |
| *ADGRE1* | 0.1018 | 0.0086 | 1.2001 | 0.0695 | 1.2726 | 0.0092 |
| *IFI27* | 0.1202 | 0.0025 | 5.8129 | 0.2843 | 1.2925 | 0.0352 |
| *ICAM1* | 0.0955 | 0.0071 | 1.2852 | 0.0766 | 1.2934 | 0.0424 |
| *CXCR6* | 0.1847 | 0.0055 | 6.2321 | 0.3468 | 1.2952 | 0.04 |
| *PDZK1IP1* | 0.2632 | 0.0167 | 4.1584 | 0.3431 | 1.3054 | 0.0291 |
| *IL12RB2* | 0.1383 | 0.0157 | 1.2226 | 0.0752 | 1.3088 | 0.0236 |
| *PLA1A* | 0.1694 | 0.0097 | 2.9703 | 0.2244 | 1.3573 | 0.0197 |
| *CD80* | 0.2926 | 0.0269 | 3.1838 | 0.3129 | 1.389 | 0.0348 |
| *TNFRSF10C* | 0.1528 | 0.0091 | 2.5638 | 0.1917 | 1.3959 | 0.0244 |
| *CCR5* | 0.103 | 0.0072 | 1.4785 | 0.0945 | 1.439 | 0.0051 |
| *IL2RA* | 0.1213 | 0.0059 | 2.4723 | 0.1702 | 1.4602 | 0.0333 |
| *CD5* | 0.1366 | 0.0039 | 4.7528 | 0.2717 | 1.4633 | 0.0208 |
| *CXCL1* | 0.0864 | 0.0044 | 1.6873 | 0.1063 | 1.5213 | 0.0304 |
| *COL6A3* | 0.1863 | 0.0147 | 2.3659 | 0.195 | 1.8061 | 0.0392 |
| *CXCL6* | 0.1359 | 0.0088 | 2.1048 | 0.1534 | 1.8751 | 0.0343 |
| *IL6* | 0.0637 | 0.0034 | 1.1948 | 0.0656 | 1.9532 | 0.0476 |
| *PTGS2* | 0.0619 | 0.0038 | 1.0159 | 0.0513 | 1.9781 | 0.029 |
| *ICAM5* | 0.2242 | 0.0336 | 1.4986 | 0.1229 | 1.9842 | 0.0441 |
| *CCL8* | 0.1737 | 0.0165 | 1.8263 | 0.1448 | 2.1059 | 0.0421 |
| *PNOC* | 0.1028 | 0.0097 | 1.0938 | 0.0593 | 2.4128 | 0.0251 |
| *C7* | 0.0484 | 0.0012 | 1.9017 | 0.1059 | 2.4763 | 0.014 |
| Log2(FC)*, Log2(fold change); CI*, confidence interval; two-sided *t*-test was conducted. Because of the small number of patients, the range of confidence interval of the cox-regression results is wide. | | | | | | |

| **S8 Table. Annotation-based unsupervised evaluation of label of clustering of genes significantly associated with PFS and label with good and poor responders.** | | | | |
| --- | --- | --- | --- | --- |
| Annotation Terms of genes  in NanoString analysis | ARS* | Enrichment rate (%)* | Significant gene* | Total gene* |
| Immune Cell Localization to Tumors | 1.000 | 6.51 | 19 | 292 |
| Recognition of Cancer Cells by T-cells | 1.000 | 8.57 | 9 | 105 |
| Myeloid Cell Activity | 1.000 | 7.31 | 19 | 260 |
| Common Signaling Pathways | 1.000 | 11.11 | 18 | 162 |
| Cancer Antigen Presentation | 0.667 | 3.96 | 4 | 101 |
| Killing of Cancer Cells | 0.667 | 6.70 | 12 | 179 |
| T-cell Priming and Activation | 0.392 | 6.67 | 10 | 150 |
| Stromal Factors | 0.392 | 3.92 | 4 | 102 |
| Tumor-Intrinsic Factors | 0.392 | 6.49 | 10 | 154 |
| Immunometabolism | 0.177 | 3.96 | 4 | 101 |
| Release of Cancer Cell Antigens | -0.036 | 8.22 | 6 | 73 |
| Cell Cycle and Proliferation | -0.036 | 11.11 | 6 | 54 |
| ARS; Adjusted Rand Score for cluster label of genes significantly associate with PFS by Cox regression analysis and label of good/poor responder, Enrichment rate; Proportion of significant gene/Total gene, Significant gene; Number of genes significantly associated with PFS within the immune cell-treated group, Total gene; Total number of genes involved in the annotation terms | | | | |


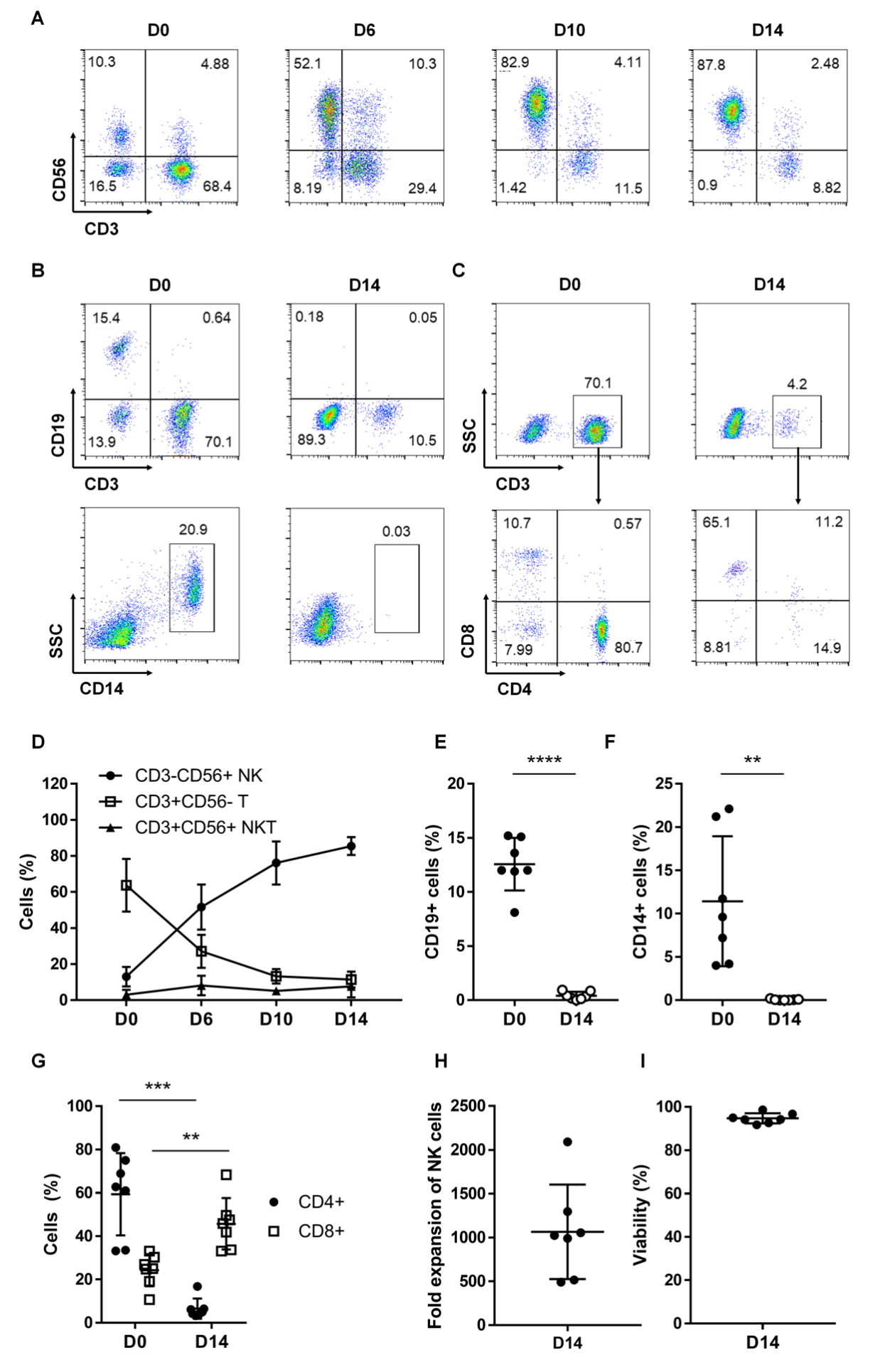


**S1 Fig. Characterization of *ex-vivo*-expanded immune cells**. During immune cell expansion process, flow cytometry was performed for the cell populations, including NK cells (CD56+CD3–), T cells (CD56–CD3+), NKT cells (CD56+CD3+), B cells, or monocytes (CD56–CD3–) in PBMCs or immune cells; representative plots at 0, 6, 10, and 14 days (A). Representative plots for PBMCs (day 0) and immune cells (day 14) are shown for CD3/CD19 and CD14/side scattered light (SSC) cell populations (B). FACS analysis of specific T cells, marked with CD4/CD8 antibodies, is shown by gating T cells (CD3+) in PBMCs (day 0) or immune cells (day 14) (C). Represents the percentages of NK, T, and NKT cells, based on fluorescence-activated cell sorting (FACS) data and cell counting in PBMCs on 0, 6, 10, and 14 days (D). The percentages of cells in PBMCs (day 0) or immune cells (day 12) was calculated based on FACS data of figures B and C (E and G). The fold change of immune cell counts relative to PBMC counts was obtained from cell counting and cell population percentages (H). Percentages of viability of immune cells at 14 days (I).


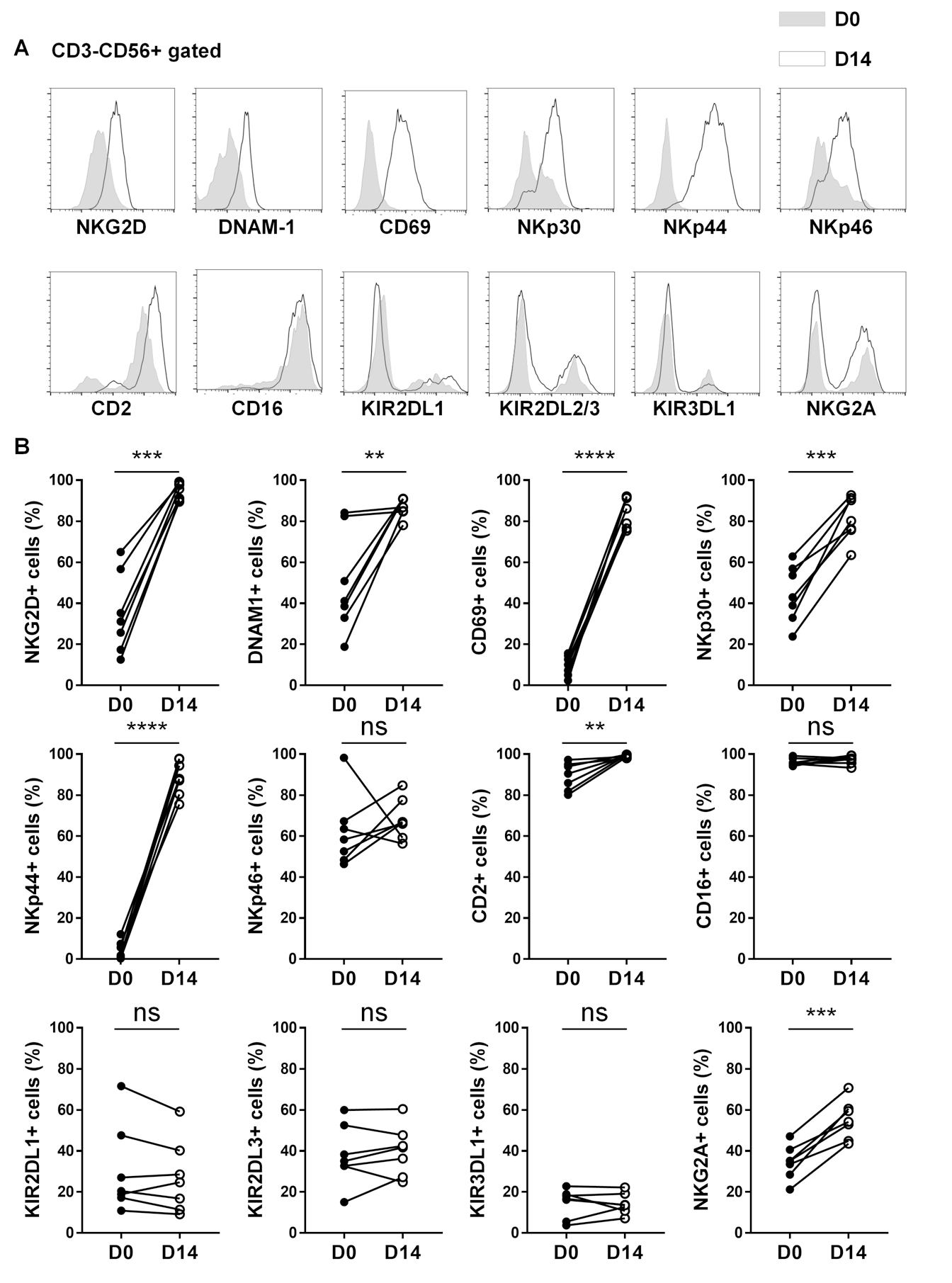


**S2 Fig. Characterization of NK ligands in *ex-vivo*-expanded immune cells from seven healthy donors.** Representative flow cytometry histograms of activating and inhibitory NK cell receptors displayed for PBMCs (grey) or *ex-vivo*-expanded immune cells (white), gated as CD3–CD56+ (A). The positive percentages of NK cell receptors were measured in PBMCs (day 0) or *ex-vivo*-expanded immune cells (day 14) (B). Statistical comparisons are shown based on paired *t*-test; ns, no significance; **p ≤ 0.005, ***p ≤ 0.0005, ****p ≤ 0.00005


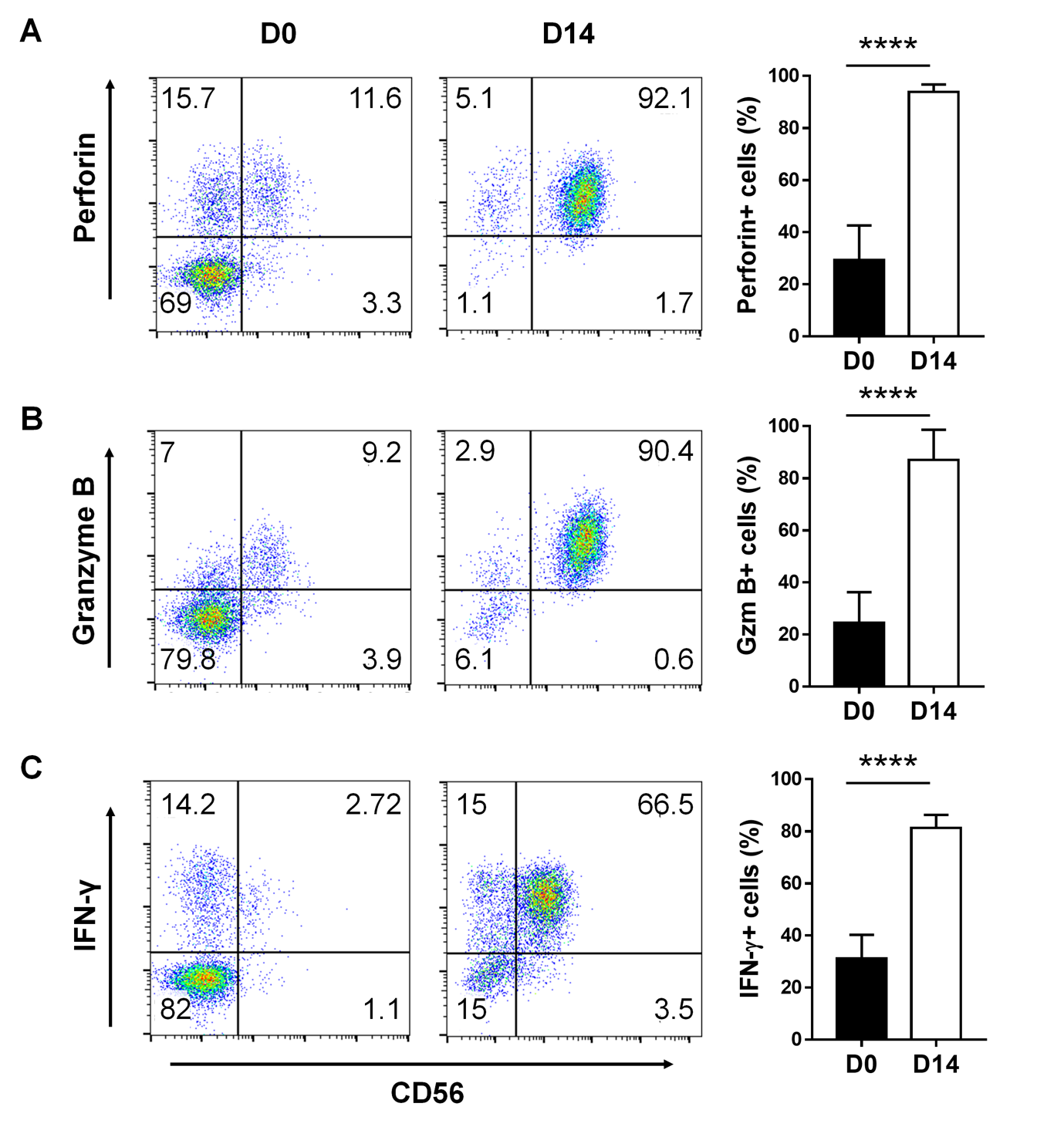


**S3 Fig. Characterization of intracellular cytotoxicity-related proteins in *ex-vivo*-expanded immune cells**. Intracellular cytotoxicity-related proteins and activation proteins were increased in *ex-vivo*-expanded immune cells, which are quantified by FACS analysis on day 0 and day 14 (A–C). Bar graphs represent % positive for the group of 7 healthy donors based on FACS analysis. ****p ≤ 0.00005 compared to PBMC group (day 0) analyzed by paired *t*-test.


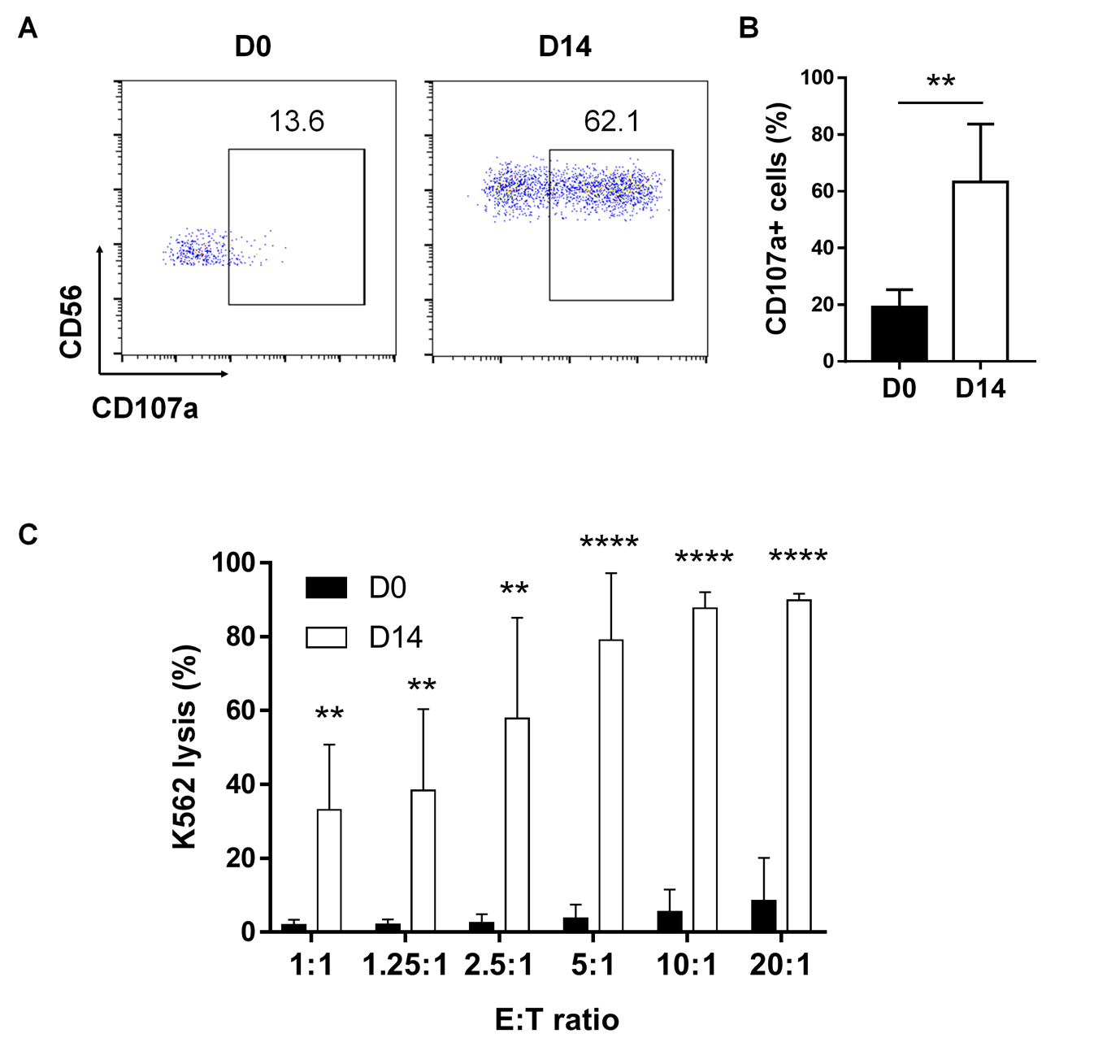


**S4** **Fig. Cytotoxicity assay of *ex-vivo*-expanded immune cells against K562 cell line.** When PBMCs (day 0) or immune cells (day 14) were co-cultured with K-562 cells, two-color flow cytometry data shows that *ex-vivo*-expanded immune cells showed higher levels of the CD56+CD107a+ cell population (A and B). The cytotoxicity assay confirmed that killing ability (K562 lysis) of immune cells (day 14) was dramatically increased compared to PBMCs (day 0) for the indicated effector: target (E: T) ratios (C). Quantification data represent mean ± SEM from 7 healthy donors. Statistical comparisons are shown; ** p ≤ 0.005, ****p ≤ 0.00005.


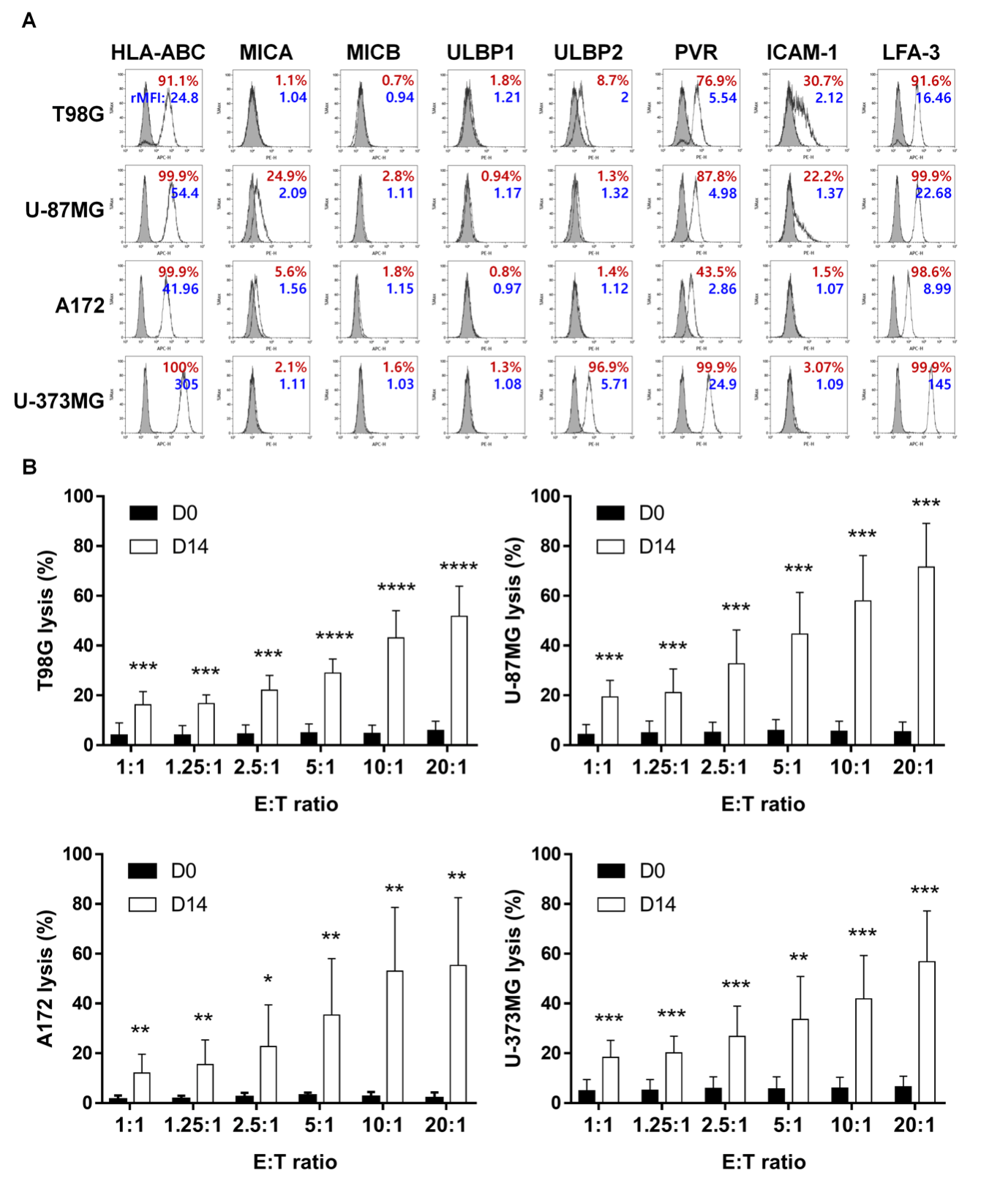


**S5 Fig. Cytotoxicity assay of *ex-vivo*-expanded immune cells against GBM cell lines.** NK cell-associated ligands were characterized in the four GBM cell lines. Isotype (grey) and antibody to each ligand (white) are showed in the histogram. The red and blue text in the histogram indicates the positive percentages and mean fluorescence intensity ratios, respectively (A). For the indicated E:T ratios, the cytolytic activity of PBMCs (day 0) or immune cells (day 14) towards the four GBM cell lines based on Figure A (B). Statistical comparisons are shown; * p ≤ 0.05, **p ≤ 0.005, ***p ≤ 0.0005, and ****p ≤ 0.00005.

**S6 Fig. Therapeutic response assessment of the first recurrent glioblastoma multiforme patient (A1) with the best prognosis (overall survival: 76 months) after adoptive immune-cell therapy.** Results of magnetic resonance imaging for evaluating the response to expanded immune-cell therapy during injection and follow-up after completion. Re-RTx, re-irradiation therapy.

**S7 Fig. MRI of A14 patients who did not receive treatment more than four times due to failure to sufficiently expand immune cells; the patient was withdrawn from the clinical study after the 12th immune cell injection.** The MRI showed fuzzy enhancement pattern with some cystic change. This patient has been alive for 22 months since the recurrence of GBM. ITx; immune cell therapy.

**S8 Fig. Therapeutic response assessment of recurrent GBM A11 patient who showed a good response to immune cell therapy (OS: 36 months).** Results of MRI for evaluating the response to immune cell therapy during injection of immune cells and follow-up after completion. Re-RTx; re-irradiation

**S9 Fig. T1-enhanced MRI after GTR or NTR of patients with poor prognosis.** Patients without durable response in GTR or NTR group showed a thick gadolinium enhancement pattern or progression along the ventricle wall enhancement. ITx; immune cell therapy.

**S10 Fig. MRI of A6 patient.** Until the final day of the immune cell treatment schedule, a marginal enhancement pattern was observed, and the thickness of enhancement area decreased. Newly thick enhancement was seen within 3 months after the completion of immune cell treatments. This lesion was confirmed as a progression by histopathology. ITx; immune cell therapy.

**S11** **Fig. T1-enhanced MRI after STR (A) or biopsy (B) patients with poor progression**. Patients without durable response in STR or biopsy groups showed a strong and thick gadolinium enhancement pattern and progression along the ventricle wall enhancement. ITx; immune cell therapy.

**S12 Fig.** **Systemic demographic of radiographic tumor response assessment in participants.**

**S13 Fig. Proportions of regression risk (hazard ratio > 1) and protective (hazard ratio < 1) effects of genes significantly associated with PFS by Cox regression analysis (A) and proportions of upregulated and downregulated genes in good responders by *t-*test (|Fold-change| > 2) (B)**

**S14 Fig.** **Volcano plot of DEG analysis of good and poor responders.** Positive and negative fold change values indicate upregulation and downregulation in good responders, respectively. The 20 labeled genes indicate genes significantly associated with PFS in both the DEG analysis between good and poor responders and in the Cox regression analysis.

**S15** **Fig. Transcriptomic landscape in GBM patients from the TCGA dataset with respect to genes identified by NanoString analysis to be significantly associated with PFS in immune cell-treated patients with recurrent GBM in this study.** Cluster map of TCGA GBM patients (A), pie plot to visualize proportions of each subtype based on gene expression clusters (B). The gene expression levels were transformed to a 0-1 standard scale.

**S16 Fig. Comparison of immune infiltration between pre-Tx (GTR) and during Tx (biopsy) tissues in A1 patient.** Immunohistochemical staining result for CD3, CD8 and CD16 with pre- and during Tx (adoptive immune cell therapy) (A), boxplot of the number of cells/high power field (HPF). * < 0.05 and ** < 0.01 (Wilcoxon signed-rank test) (B)
