# Supplementary material for: Autologous adoptive immune-cell therapy elicited a durable response with enhanced immune reaction signatures in patients with recurrent glioblastoma: An open label, phase I/IIa trial
Source: PLoS One. 2021 Mar 10;16(3):e0247293. doi: 10.1371/journal.pone.0247293 (PMC7946298; doi:10.1371/journal.pone.0247293)
Supplement: S2 File — (PDF) [file pone.0247293.s002.pdf]

Protocol

Manuscript Title

Autologous adoptive immune-cell therapy elicited durable response with enhanced immune reaction signatures in recurrent glioblastoma patients

For review, we have extracted the above statistical sections from both the original and final protocols.

This supplement contains the following items:

1. Statistical plan from the original protocol (November 1, 2013) ------------------------- page 3
2. Statistical plan from the final protocol (July 12, 2018) ------------------------------------- page 6
3. Original protocol (November 1, 2013) -- page 9
4. Final protocol (July 12, 2018) -- page 44
5. Summary of protocol amendments -- page 83

1. Statistical method section from original protocol

10.2 Statistical analysis method

10.2.1 General principles for results analysis

The data obtained from the subjects through this clinical trial are largely divided into three groups: Safety, Full Analysis (FA) and Per-Protocol (PP). The main population of analysis in this clinical trial is the FA group, while additional analysis is performed on the PP group and safety is analyzed based on the Safety group. However, in the case of the FA group for the primary effectiveness assessment, the analysis group shall be redefined by setting forth the exclusion criteria for the subjects taking into consideration of the characteristics of the assessment variables. The results of all three groups shall be compared, and when the results differ among the groups, the results of each analysis method shall be presented with the reasons for the differences described in detail. All of the analyses shall be performed at a significance level of 5%.

Assignment to the FA Group:
Those who have been administered the investigational drug at least once after being registered as a subject in this clinical trial and had the variables for the effectiveness assessment measured at least once after the administration shall be included in the analysis. For the FA group, the intention-to-treat (ITT) shall be a fundamental principle.
However, the FA group for the primary effectiveness assessment, the following subjects shall additionally be excluded.

① A subject who no longer meets the selection criteria or meets the exclusion criteria
② A subject who has never been administered the drug used in the clinical trial after registration in the study
③ A subject without any measurement values for effectiveness assessment after registration in the study

Assignment to the PP Group:
The PP group is comprised of subjects included in the FA group that have completed the clinical trial in accordance with the clinical trial protocol. However, subjects falling under any of the following shall be excluded:

① A subject whose investigational drug administration has been postponed or cancelled three consecutive times or more or four or more times in total during the clinical trial period
② A subject who did not recover from an adverse event even after 4 weeks
③ A subject who dropped out or was removed from the clinical trial before fulfilling the duration specified in the protocol
④ A subject who no longer meets the selection criteria or meets the exclusion criteria
⑤ Any other cases that could be considered a serious violation of the protocol

Assignment to the Safety Group:
Those who have been administered the investigational drug at least once shall be included in the analysis.

Handling of missing values
If there is a missing value at any point or a missing value occurs due to a subject dropping out or being removed before the completion of the clinical trial, the missing data shall be substituted using the Last Observation Carried Forward (LOCF) method.

Analysis point
Statistical analysis will be performed 12 months after the last patient is enrolled in this study.

10.2.2 Demographic data

In the case of continuous data, the descriptive statistics (mean, standard deviation, minimum value, maximum value, etc.) shall be presented according to the time of visit, while in the case of categorical data, the frequency and ratio for each category shall be presented.

10.2.3 Safety data

10.2.3.1 Adverse events

In this clinical trial, adverse events shall be classified into 1) adverse events prior to administration of the investigational drug (i.e. underlying symptoms and signs) and 2) treatment emergent adverse events (TEAE). TEAE shall be defined as ① an adverse reaction that occurred for the first time after the administration of the investigational drug and ② an adverse event in which the severity of underlying symptoms and signs that have occurred before the administration of the investigational drug has worsened after the administration. In the analysis of the adverse events, TEAEs shall be analyzed and all of the adverse events shall be presented as a list.

In case of multiple occurrences of the same adverse event to a subject, the event shall be regarded as a single adverse event. If the severity of the same adverse event differs, the worst severity shall be noted. If the causality of the same TEAE in a subject is different, the causality that is most relevant to the investigational drug shall be noted.

The percentage of subjects who experienced an adverse event and the 95% confidence interval in relation thereto shall be presented. All of the adverse events that have occurred shall be documented according to the level of severity. The adverse events and serious adverse events associated with the investigational drug shall also be documented.

10.2.3.2 Clinical laboratory tests

In the case of continuous data such as the hematological or hematobiochemical test results, the descriptive statistics (mean, standard deviation, minimum value, maximum value, etc.) shall be presented, whereas in the case of categorical data such as the urine test and 12-lead ECG results, the frequency and ratio for each category shall be presented.

For continuous variables, a paired t-test or Wilcoxon signed-rank test shall be performed on the differences between before and after the administration of the investigational drug to examine the differences between the visits. McNemar's test shall be carried out for categorical variables. In addition, clinically significant non-normalized rates compared to before treatment shall be presented.

10.2.3.3 Vital signs and physical examination

The descriptive statistics for vital signs (blood pressure, pulse, and body temperature) shall be presented, and a shift table indicating the changes between before and after the administration of the investigational drug with respect to the normal and abnormal values measured in the physical examination shall be presented. The before and after differences shall be analyzed by a paired t-test or Wilcoxon signed-rank test for continuous variables and the McNemar's test for categorical variables.

10.2.4 Effectiveness data

<u>Progression-free survival (PFS)</u>

PFS shall be estimated by the Kaplan-Meier method, and the median survival time shall be calculated.

<u>Response rate</u>

The degree and percentage of the subjects who showed a response to treatment shall be presented, with the 95% confidence interval indicated.

<u>Overall survival (OS)</u>

OS shall be estimated by the Kaplan-Meier method, and the median survival time shall be calculated. OS shall be the period between the date on which the individual was registered as a subject of this clinical trial and assigned a number and the date on which he/she died, irrespective of the cause.

<u>QLQ-C30 (Ver 3.0) Korean</u>

The measurements for each item shall be compared in order to determine the level, and the changes according to the progress of treatment shall be analyzed. The descriptive statistics (mean, standard deviation, median, minimum value, and maximum value) shall be presented with respect to the QLQ-C30 (Ver 3.0) Korean evaluation scores and be tested using an independent t-test or Wilcoxon's rank sum test depending on the presence of normality. As for making a comparison between before and after the administration of the

investigational drug, a paired t-test or Wilcoxon's signed-rank test shall be performed.

<u>Karnofsky performance status (KPS)</u>
The measurements for each item shall be compared in order to determine the level, and the changes according to the progress of treatment shall be analyzed. The descriptive statistics (mean, standard deviation, median, minimum value, and maximum value) shall be presented with respect to the KPS evaluation scores and be tested using an independent t-test or Wilcoxon's rank sum test depending on the presence of normality. As for making a comparison between before and after the administration of the investigational drug, a paired t-test or Wilcoxon's signed-rank test shall be performed.

2. Statistical method section from final protocol

10.2 Statistical analysis method

10.2.1 General principles for results analysis

The data obtained from the subjects through this clinical trial are largely divided into three groups: Safety, Full Analysis (FA) and Per-Protocol (PP). The main population of analysis in this clinical trial is the FA group, while additional analysis is performed on the PP group and safety is analyzed based on the Safety group. However, in the case of the FA group for the primary effectiveness assessment, the analysis group shall be redefined by setting forth the exclusion criteria for the subjects taking into consideration of the characteristics of the assessment variables. The results of all three groups shall be compared, and when the results differ among the groups, the results of each analysis method shall be presented with the reasons for the differences described in detail. All of the analyses shall be performed at a significance level of 5%.

Assignment to the FA Group:

Those who have been administered the investigational drug at least once after being registered as a subject in this clinical trial and had the variables for the effectiveness assessment measured at least once after the administration shall be included in the analysis. For the FA group, the intention-to-treat (ITT) shall be a fundamental principle.

However, the FA group for the primary effectiveness assessment, the following subjects shall additionally be excluded.

⑥ A subject who no longer meets the selection criteria or meets the exclusion criteria

⑦ A subject who has never been administered the drug used in the clinical trial after registration in the study

⑧ A subject without any measurement values for effectiveness assessment after registration in the study

Assignment to the PP Group:

The PP group is comprised of subjects included in the FA group that have completed the clinical trial in accordance with the clinical trial protocol. However, subjects falling under any of the following shall be excluded:

④ A subject whose investigational drug administration has been postponed or cancelled two or more times in each cycle during the clinical trial period

⑤ A subject whose investigational drug administration has been postponed or cancelled three consecutive times or more or four or more times in total during the clinical trial period

⑥ A subject who did not recover from an adverse event even after 4 weeks

⑦ A subject who dropped out or was removed from the clinical trial before fulfilling the duration specified in the protocol

⑨ A subject who no longer meets the selection criteria or meets the exclusion criteria

⑧ Any other cases that could be considered a serious violation of the protocol

Assignment to the Safety Group:

Those who have been administered the investigational drug at least once shall be included in the analysis.

Handling of missing values

If there is a missing value at any point or a missing value occurs due to a subject dropping out or being removed before the completion of the clinical trial, the missing data shall be substituted using the Last Observation Carried Forward (LOCF) method.

Interim Analysis

1) Purpose of the Interim Analysis

The research team has planned an interim analysis for the purpose of checking for any correlation between the administration of the investigational drug and the death cases and extended survival period by performing an analysis of the 14 subjects after completing an assessment of 14 of the 23 subjects. The aim is to assess the futility of using the investigational drug and to decide on whether to continue with the clinical trial, based on the results

of the interim analysis. If the results indicate that it is futile to use the investigational drug, the clinical trial shall be terminated after completing the program for the current subjects, without registering any additional patients. In contrast, if it is not deemed to be futile, then the clinical trial shall be continued until completion and an additional clinical trial plan will be set forth for glioblastoma patients.

2) Method of the Interim Analysis
The interim analysis shall be carried out after completing the necessary assessment for 14 of the 23 subjects. The interim analysis and the final analysis shall be performed on the variables in the safety assessment, which are adverse reactions, clinical trial lab test results, measurements of vital signs, and physical examination results, as well as the variables in the efficacy assessment such as OS, PFS, response rate, QLQ-C30 (ver 3.0) Korean, KPS (Karnofsky performance status), and immune reaction evaluation. The assessment items and statistical analysis method applied to the interim analysis and the final analysis shall be identical. The final analysis shall be conducted after the completion of F/U for all subjects.

3) Multiplicity Test
Adjustment for multiplicity is not needed for this study, as it is a Phase 1/2 exploratory clinical trial.

4) Assessment Criteria and Follow-up Measures
The final decision on whether to continue on with the study, based on the results of the interim analysis, shall be made by the investigator, based on the direction of the clinical trial presented after an analysis of the safety and therapeutic effects of the investigational drug.
As for the futility assessment, it will be deemed that the use of the investigational drug is futile in the event of a serious adverse event in relation to the investigational drug or the mOS is lower than the weighted average (8.3 months) of the therapeutic agents reported in the literature. If the results of the interim analysis indicate the futility of the use of the investigational drug, the clinical trial shall be terminated after completing the program for the current subjects, without registering any additional patients. If not, the clinical trial shall be continued according to the protocol.

10.2.2 Demographic data
In the case of continuous data, the descriptive statistics (mean, standard deviation, minimum value, maximum value, etc.) shall be presented according to the time of visit, while in the case of categorical data, the frequency and ratio for each category shall be presented.

10.2.3 Safety data

10.2.3.1 Adverse events
In this clinical trial, adverse events shall be classified into 1) adverse events prior to administration of the investigational drug (i.e. underlying symptoms and signs) and 2) treatment emergent adverse events (TEAE). TEAE shall be defined as ① an adverse reaction that occurred for the first time after the administration of the investigational drug and ② an adverse event in which the severity of underlying symptoms and signs that have occurred before the administration of the investigational drug has worsened after the administration. In the analysis of the adverse events, TEAEs shall be analyzed and all of the adverse events shall be presented as a list.
In case of multiple occurrences of the same adverse event to a subject, the event shall be regarded as a single adverse event. If the severity of the same adverse event differs, the worst severity shall be noted. If the causality of the same TEAE in a subject is different, the causality that is most relevant to the investigational drug shall be noted.
The percentage of subjects who experienced an adverse event and the 95% confidence interval in relation thereto shall be presented. All of the adverse events that have occurred shall be documented according to the level of severity. The adverse events and serious adverse events associated with the investigational drug shall also be documented.

10.2.3.2 Clinical laboratory tests
In the case of continuous data such as the hematological or hematobiochemical test results, the descriptive statistics (mean, standard deviation, minimum value, maximum value, etc.) shall be presented, whereas in the case of categorical data such as the urine test and 12-lead ECG results, the frequency and ratio for each category

shall be presented.
For continuous variables, a paired t-test or Wilcoxon signed-rank test shall be performed on the differences between before and after the administration of the investigational drug to examine the differences between the visits. McNemar's test shall be carried out for categorical variables. In addition, clinically significant non-normalized rates compared to before treatment shall be presented.

10.2.3.3 Vital signs and physical examination

The descriptive statistics for vital signs (blood pressure, pulse, and body temperature) shall be presented, and a shift table indicating the changes between before and after the administration of the investigational drug with respect to the normal and abnormal values measured in the physical examination shall be presented. The before and after differences shall be analyzed by a paired t-test or Wilcoxon signed-rank test for continuous variables and the McNemar's test for categorical variables.

10.2.4 Effectiveness data

Progression-free survival (PFS)
PFS shall be estimated by the Kaplan-Meier method, and the median survival time shall be calculated.

Overall survival (OS)
OS shall be estimated by the Kaplan-Meier method, and the median survival time shall be calculated. OS shall be the period between the date on which the individual was registered as a subject of this clinical trial and assigned a number and the date on which he/she died, irrespective of the cause.

Response rate
The degree and percentage of the subjects who showed a response to treatment shall be presented, with the 95% confidence interval indicated.

QLQ-C30 (Ver 3.0) Korean
The measurements for each item shall be compared in order to determine the level, and the changes according to the progress of treatment shall be analyzed. The descriptive statistics (mean, standard deviation, median, minimum value, and maximum value) shall be presented with respect to the QLQ-C30 (Ver 3.0) Korean evaluation scores and be tested using an independent t-test or Wilcoxon's rank sum test depending on the presence of normality. As for making a comparison between before and after the administration of the investigational drug, a paired t-test or Wilcoxon's signed-rank test shall be performed.

Karnofsky performance status (KPS)
The measurements for each item shall be compared in order to determine the level, and the changes according to the progress of treatment shall be analyzed. The descriptive statistics (mean, standard deviation, median, minimum value, and maximum value) shall be presented with respect to the KPS evaluation scores and be tested using an independent t-test or Wilcoxon's rank sum test depending on the presence of normality. As for making a comparison between before and after the administration of the investigational drug, a paired t-test or Wilcoxon's signed-rank test shall be performed.

Evaluation of immune reaction
Statistical analysis of the immune reaction evaluation: The analysis can be carried out by a repeated measure of ANOVA in relation to the change from baseline or measured value, depending on the nature of the variable, or the X2 test or Fisher's exact test may be applied to compare the frequency of certain levels between the groups or immune reactions.

3. Original protocol (version 1.1)

Clinical Trial Protocol

Investigator Clinical Phase 1/2 Study of Immunotherapy Using Autologous Killer Cell in Patients with Recurrent Glioblastoma

Protocol No.: The Autologous Killer Cell
Version 1.1_2013.01.04

Confidentiality Statement
The information contained in this clinical trial protocol is solely intended as a guidance for the head of the testing agency, principal investigator, sub-investigators, managing pharmacist, related administrative agencies, and Institutional Review Board (IRB) of the clinical trial institution.<br>This protocol may not be disclosed to any party unrelated to the clinical trial without obtaining written consent, except in cases where it is necessary to obtain written consent from the subjects.

Contents

1. Summary 14
2. Schedule 20
3. Background of the clinical trial 22
4. Purpose of the clinical trial 23
5. Clinical trial period 24
6. Medicinal products used in the clinical trial 24
6.1 Investigational drug 24
6.2 Investigational drug management and records 24
6.3 Production, packaging and labeling of the investigational drug 24
7. Subject selection criteria, exclusion criteria, target number of subjects, and calculation basis 24
7.1 Selection criteria 24
7.2 Exclusion criteria 25
7.3 Target number of subjects 25
7.4 Basis for calculating the target number of subjects 25
7.5 Random assignment 25
7.6 Clinical trial design 25
7.7 Basis for setting the dose 26
7.8 Method and duration of administration 27
7.9 Conservative treatment 27
7.10 Drugs that can be used in combination 27
7.11 Drugs that cannot be used in combination 27
7.12 Matters for observation and observation method 28
7.12.2.1 Written consent and demographic survey 30
7.12.2.2 Investigation of medical and medication history 30
7.12.2.3 Measurement of vital signs 30
7.12.2.4 Physical examination 30
7.12.2.5 Pregnancy test and clinical laboratory tests 30
7.12.2.6 Tumor evaluation 31
7.12.2.7 KPS, QLQ-C30 (ver 3.0) Korean 31
7.12.2.8 Screening based on selection/exclusion criteria (subject suitability assessment) 31
7.12.2.9 Investigational drug 31
7.12.2.10 Dose-limiting toxicity assessment 31
7.12.2.11 Investigation into adverse events 31
7.12.3 Clinical trial method by visit 32
8. User precautions for the medicinal products used in the clinical trial 33
9. Withdrawal and termination criteria and criteria for exclusion from analysis 34
9.1 Subject withdrawal (drop-out) and trial termination criteria 34
9.2 Compliance in the clinical trial and handling a protocol violation 34
10. Effectiveness assessment criteria, assessment method and analysis method (statistical analysis method) 34
10.1 Assessment criteria 34
10.1.1 Safety assessment variables 34
10.1.2 Effectiveness assessment variables 35
10.2 Statistical analysis method 35
10.2.1 General principles for results analysis 35
10.2.3 Safety data 36
10.2.4 Effectiveness data 36
11. Safety assessment method and criteria and reporting method incl. adverse events 37
11.1 Terms related to safety and definitions thereof 37
11.2 Assessment method 37

11.3 Assessment criteria 38
11.3.1 Fever 38
11.3.2 Subjective and objective symptoms 38
11.4 Causal relationship with the investigational drug 39
11.5 Reporting method 39
11.5.1 Reporting adverse events 39
11.6 Measures to be taken for serious ADRs 41
12. Written consent, compensation agreement, and post-trial medical examination and treatment of the subjects 41
12.1 Consent form and written explanations 41
12.2 Agreement on compensation 41
12.3 Post- trial medical examination and treatment of the subjects 41
13. Measures for ensuring the safety of the subjects 42
13.1 Clinical trial institution 42
13.2 Approval of and amendments to the clinical trial protocol 42
13.3 Familiarization of the clinical trial protocol 42
13.4 Consent to participate in the clinical trial 42
13.5 Accurate selection of subjects 42
13.6 Checking the progress of the clinical trial 42
13.7 Monitoring of the clinical trial institution 42
13.8 Confidentiality 42
13.9 Measures taken in the event of an adverse event 43
14. Matters necessary for conducting the clinical trial in a safe and scientific way 43
15. References 43
16. Appendix 43

Acronyms

Acronym	Definition
ADE	Adverse Device Effect
ADR	Adverse Drug Reaction
AE	Adverse Event
BMI	Body Mass Index
BUN	Blood Urea Nitrogen
CRF	Case Report Form
CRO	Contract Research Organization
CTCAE	Common Terminology Criteria of Adverse Event
DLT	Dose Limiting Toxicity
FAS	Full Analysis Set
IRB	Institutional Review Board
ITT	Intent To Treat
KGCP	Korea Good Clinical Practice
KPS	Karnofsky Performance Status
OS	Overall Survival
PFS	Progression-free survival
PP	Per-Protocol
PT	Preferred Term
QOL	Quality Of Life
RBC	Red Blood Cell
SAE	Serious Adverse Event
WBC	White Blood Cell

1. Summary

Clinical trial title	Investigator Clinical Phase 1/2 Study of Immunotherapy Using Autologous Killer Cell in Patients with Recurrent Glioblastoma
Implementing agency	CHA Bundang Medical Center (CBMC) of CHA University, 59, Yatap-ro, Bundang-gu, Seongnam-si, Gyeonggi-do, Republic of Korea
Principal investigator	CHO Kyung Gi, Professor of Neurosurgery, CHA Bundang Medical Center (CBMC) of CHA University / 031-780-5263
Co-investigators	Park YoungSuk, Professor of Neurosurgery, CHA Bundang Medical Center (CBMC) of CHA University
Purpose	To administer the autologous killer cell, the investigational drug, to patients with recurrent glioblastoma and monitor the safety and effectiveness of the immunotherapy
Background of research	Glioma accounts for approx. 35% of all primary brain tumors, while glioblastoma accounts for around 40% of all glioma cases. Glioblastoma, which occurs commonly in men aged between 45 and 65, is the most aggressive malignant glioma and is very difficult to treat. Although glioblastomas form a mass in some cases, most exhibit invasive growth into the surrounding tissue, rendering treatment by a localized surgical procedure impossible.<br>With the introduction of nitrosoureas in the early 1970s, numerous clinical trials have been carried out with patients suffering from primary or recurrent malignant brain tumors in order to examine the therapeutic effects of anticancer agents. According to a meta-analysis, chemotherapy is recommended in combination with radiation therapy following surgery, but the 5-year survival rate is very low at under 2%. Thus, the purpose of treatment for glioblastoma, which has a grim prognosis as such, is to extend the survival period as much as possible and to manage the disease so that the patient's quality of life does not deteriorate while he/she is still alive.<br>Weller M. et al. (Neuro Oncol. 2012 Nov 7. [Epub ahead of print]) reviewed the abstracts on PubMed and American Society of Clinical Oncology from Jan. 2006 until Jan. 2012, and examined the clinical trials that used anticancer drugs such as nitrosoureas, which are the most common type of drug prescribed for recurrent glioblastoma patients, as well as temozolomide and bevacizumab, which have recently been developed and are now used regularly.<br>It has been reported that reoperation or reirradiation is attempted for some patients and there is increased toxicity from the anticancer drugs used afterwards, but the progression-free survival rate at 6 months is only 20~30%. The aim of this study is, therefore, to try a new type of immunotherapy in such cases where further treatment cannot be anticipated in the event of recurrence.<br>With respect to the immune function of the human body, basic research has been conducted on not only pathogens that enter the body from the outside, but also malignant tumors that arise from inside the body, and the findings of some of the studies are applied clinically. Based on the fact that many studies have shown that immune cells attack tumors, there have been a number of studies on prophylactic treatment using agents that specifically or nonspecifically enhance immunity with the involvement of lymphocytes and phagocytes. In addition, one of the most active fields of research today is to proliferate specific immune cells by cytokines in peripheral blood and to apply them to tumor immunity.<br>The cellular immunotherapy agent used in this study is the autologous killer cell (AKC), a type of autologous activated lymphocyte obtained by isolating lymphocytes from the peripheral blood and inducing selective proliferation of the lymphocytes, NK, NKT and T cells. Of these, the natural killer (NK) cells are the immune cells playing the primary function of AKC. AKC was cultured after adding a specific antigen and IL-2 to induce proliferation of the immune cells.<br>Although the safety of domestic and foreign autologous immune cell therapy agents

	has been reported by a number of clinical trials, the safety of AKC specifically produced by CHA Bundang Medical Center's GMP facility has yet to be directly established and thus, securing safety is most important in this clinical trial. In addition, tumor is typically treated by surgery, radiotherapy, or chemotherapy, but most malignant tumors are difficult to treat with the above therapies alone due to their anatomical location, which makes it essential to perform adjuvant therapy to improve the prognosis. Among the available methods for adjuvant therapy, adoptive immunotherapy by autologous activated immune cells is very effective. This method of therapy is easy to administer and associated with fewer adverse effects than conventional chemotherapy and thus is expected to help improve the prognosis of cancer.
Clinical trial period	48 months from the date of approval by the Ministry of Food and Drug Safety (MFDS) and the IRB (the duration of the clinical trial or the registration period may vary)
Investigational drug	The investigational drug in this study is the AKC, a self-activating lymphocyte for cellular immunotherapy extracted from the patient's own blood
Research method (administration method, dose, duration of administration, and concomitant therapy)	1. If more than 1 x 10^{9} cells of a single dose are administered to 3 subjects and safety is confirmed through an 8-week dose-limiting toxicity assessment, an additional 20 subjects are listed and safety and efficacy are assessed. However, in the case of 1 dose of DLT (Dose Limiting Toxicity) observed in 1 of 3 subjects, 3 persons were added to evaluate the dose safety for a total of 6 subjects.<br>2. The Autologous Killer Cell is obtained from taking 60 ml of the patient's blood once every two weeks by culturing for 2 weeks, which is intravenously injecting more than 1 x 10^9 cells of the set dose. The number of injections of the immune cell therapeutic agent that the subject can receive the maximum dose is 24 times.<br>3. Tumor assessment shall be performed once every three months using MRI.
Target disease/population	Patients with recurrent glioblastoma
Number of subjects	23

Selection criteria	1) Individuals aged 20 years or older but under 70<br>2) Individuals for whom the standard therapy failed after being diagnosed with recurrent glioblastoma based on an MRI scan<br>3) Individuals whose survival period is at least 3 months<br>4) Individuals with an appropriate bone marrow function<br>- Hemoglobin count > 10 g/dL<br>- Leukocyte count > 3,000/mm^3<br>- Absolute neutrophil count > 1,500/ML<br>- Platelet count > 75.000/mm3<br>5) Individuals with appropriate liver and kidney functions<br>- Total bilirubin < 1.5 times the normal upper limit specified by the testing agency<br>- AST, ALT < 2.5 times the normal upper limit specified by the testing agency<br>- Alkaline phosphatase < 1.5 times the normal upper limit specified by the testing agency<br>- Normal kidney function (serum creatinine < normal upper limit specified by the testing agency)<br>6) Individuals who have obtained a sufficient explanation of the purpose and details of the clinical trial and the characteristics of the investigational drug from an investigator and who signed the consent form or had a legal guardian or representative sign the consent form prior to the beginning of the clinical trial<br>7) In the case of women, they must have been tested negative in a urine or blood test for pregnancy seven days before the beginning of the clinical trial and agreed to use an appropriate method of contraception during the clinical trial<br>8) Individuals who have not had any blood infection within the last 6 months<br>9) Individuals who have not received any other forms of immunotherapy
Exclusion criteria	1) An individual who is deemed to have a severe cardiopulmonary dysfunction by an investigator<br>2) An individual with immune deficiency or an autoimmune disease that may be exacerbated by immunotherapy (e.g. rheumatoid arthritis, systemic lupus erythematosus, vasculitis, multiple sclerosis, juvenile-onset insulin-dependent diabetes mellitus, etc.)<br>3) A carrier of chronic hepatitis B and/or C or who is HIV antibody positive<br>4) An individual with history of severe allergic reactions<br>5) An individual with uncontrolled hypertension or diabetes<br>6) A pregnant or lactating woman<br>7) An individual who is considered to be unfit for the clinical trial due to a serious medical or psychiatric condition<br>8) An individual with a major hemorrhagic disease not associated with cancer<br>9) An individual who has participated in another clinical trial within 4 weeks before the start of this clinical trial

Matters for observation, clinical examination items and observation method	1. Vital signs and physical examination<br>The vital signs and physical examination shall be performed at the screening visit, at every administration of the investigational drug, and at every F/U visit.<br>(1) Vital signs: Blood pressure, pulse, respiration, and body temperature<br>(2) Physical examination: Cardiovascular, respiratory, gastro-intestinal, central & peripheral nervous system, genitourinary, ENT, skin, etc.<br>1) Radiographic examination<br>- General X ray - chest PA, abdomen erect, KUB<br>- Chest CT with contrast enhancement<br>- PRN - PET/CT, bone scan, CT, sonogram, etc. at the discretion of the investigator<br>2) Blood test<br>- Hematological tests: Hb, Hct, WBC, RBC, PLT, ESR, differential count<br>- Hematobiochemical tests: Glucose, AST, ALT, ALP, albumin, total bilirubin (BUN), creatinine, Na, K, Cl, Ca, total cholesterol, triglyceride, HDL, LDL, uric acid<br>3) Urine test: Color, specific gravity, pH, glucose, protein, β-hCG<br>4) Blood coagulation test: PT/aPTT<br>5) Autoimmune reaction test<br>RF (rheumatoid factor), ANA (antinuclear antibody), FT4, TSH, T3, CRP<br>6) Virus test (only at screening): HBs Ag, HBs Ab, anti-HCV, anti-HIV<br>(3) Electrocardiogram (ECG)<br>ECG is performed to evaluate the patient's cardiac function during screening. If an abnormality that has been found is not clinically significant, the patient shall be deemed to meet the selection criteria. If necessary, an additional test/examination shall be performed to assess cardiac function.<br>(4) Pregnancy test<br>During screening, women of childbearing age shall be subject to a blood or urine test (P-hCG) to check for pregnancy.<br>(5) Routine urinalysis<br>(6) Check whether the patient is taking any medications<br>The medications that each patient is currently taking shall be checked during the administration period of the investigational drug and on each day of administration of the investigational drug, and the details shall be recorded in the case records. Obtain details of the dose, reason for intake, start date of treatment, etc. in relation to the medications the patient is taking and make records thereof in the case records.<br>2. Assessment of the chemotherapy response<br>The anticancer therapy response is assessed by CT or MRI, while the immune reaction is assessed based on T-cell proliferation assay and ELISPOT test in patient's blood.
Withdrawal (drop-out) and termination criteria	1) Evidence of tumor progression<br>2) Request of subject<br>3) Occur the severe toxicity<br>4) According to investigator
Safety and effectiveness assessment criteria	The anticancer therapy response is assessed by CT or MRI, while the immune reaction is assessed based on T-cell proliferation assay and ELISPOT test in patient's blood.<br>1. Safety assessment<br>Adverse reactions: Fever, subjective and/or objective symptoms, causal relationship with the investigational drug, etc.<br>Clinical laboratory tests<br>Vital signs and physical examination<br>2. Effectiveness assessment<br>Progression-free survival<br>Overall survival<br>Treatment response rate according to the tumor evaluation<br>QLQ-C30 (Ver 3.0) Korean

	Karnofsky performance status (KPS)<br>Evaluation of immune reaction
Safety and effectiveness assessment and analysis methods (methods of statistical analysis)	<u>General principles</u><br>The data obtained from the subjects through this clinical trial are largely divided into three groups: Safety, Full Analysis (FA) and Per-Protocol (PP). The main population of analysis in this clinical trial is the FA group, while additional analysis is performed on the PP group and safety is analyzed based on the Safety group. However, in the case of the FA group for the primary effectiveness assessment, the analysis group shall be redefined by setting forth the exclusion criteria for the subjects taking into consideration of the characteristics of the assessment variables. The results of all three groups shall be compared, and when the results differ among the groups, the results of each analysis method shall be presented with the reasons for the differences described in detail. All of the analyses shall be performed at a significance level of 5%.<br><u>Safety assessment method</u><br>1) Adverse events: The percentage of subjects who experienced an adverse event and the 95% confidence interval in relation thereto shall be presented. All of the adverse events that have occurred shall be documented according to the level of severity. The adverse events and serious adverse events associated with the investigational drug shall also be documented.<br>2) Clinical laboratory tests: In the case of continuous data, the descriptive statistics (mean, standard deviation, minimum value, maximum value, etc.) shall be presented according to the time of visit, while in the case of categorical data, the frequency and ratio for each category shall be presented. For continuous variables, a paired t-test or Wilcoxon signed-rank test shall be performed on the differences between before and after the administration of the investigational drug to examine the differences between the visits. McNemar's test shall be carried out for categorical variables. In addition, clinically significant non-normalized rates compared to before treatment shall be presented.<br>3) Vital signs and physical examination: The descriptive statistics for vital signs (blood pressure, pulse, and body temperature) shall be presented, and a shift table indicating the changes between before and after the administration of the investigational drug with respect to the normal and abnormal values measured in the physical examination shall be presented. The before and after differences shall be analyzed by a paired t-test or Wilcoxon signed-rank test for continuous variables and the McNemar's test for categorical variables.<br><br><u>Effectiveness assessment method</u><br>1) Overall survival (OS): OS shall be the period from the date on which the individual was registered as a subject of this clinical trial and assigned a number to the date on which he/she died, irrespective of the cause. OS shall be estimated by the Kaplan-Meier method, and the median survival time shall be calculated.<br>2) Progression-free survival (PFS): PFS shall be the period from the date on which the individual was registered as a subject of this clinical trial and assigned a number to the date on which a lesion arose or on which he/she died. The progress of the glioblastoma shall be assessed before and after the administration of the investigational drug at Week 12, Week 24, Week 36, Week 48, Week 60, and Week 68 based on a CT or MRI scan. The PFS of patients without any progress or recurrence of tumor at final analysis shall be determined as the period from the date on which he/she was registered as a subject of this clinical trial and assigned a number to the date of the final tumor assessment. PFS, without any progression of the disease, shall be estimated by the Kaplan-Meier method, and the median survival time shall be calculated.<br>3) Response rate: A tumor response assessment shall be carried out, in accordance with mRECIST (Appendix 3), before and after the administration of the investigational drug at Week 12, Week 24, Week 36, Week 48, Week 60, and Week 68 based on a CT or MRI scan. The disease control rate (DCR), which is defined as the ratio of the subjects displaying an overall response that is classified as CD, PR or SD to the total

	number of subjects, shall be presented, with the 95% confidence interval indicated.<br>4) QLQ-C30 (Ver 3.0) Korean: The measurements for each item shall be compared in order to determine the level, and the changes according to the progress of treatment shall be analyzed. The descriptive statistics (mean, standard deviation, median, minimum value, and maximum value) shall be presented with respect to the QLQ-C30 (Ver 3.0) Korean evaluation scores and depending on the presence of normality, a paired t-test or Wilcoxon's signed-rank test shall be performed for a comparison between before and after the administration of the investigational drug.<br>5) Karnofsky performance status (KPS): The measurements for each item shall be compared in order to determine the level, and the changes according to the progress of treatment shall be analyzed. The descriptive statistics (mean, standard deviation, median, minimum value, and maximum value) shall be presented with respect to the KPS evaluation scores and depending on the presence of normality, a paired t-test or Wilcoxon's signed-rank test shall be performed for a comparison between before and after the administration of the investigational drug.
Measures for ensuring safety of subjects	In case any of the subjects in this clinical trial suffers from an adverse event with a causal relationship with the investigational drug used in this clinical trial, notwithstanding the fact that the investigators have complied with the relevant laws and regulations and performed the clinical trial in strict adherence to the protocol, the principal investigator shall follow the Victim Compensation Regulations submitted to the IRB and provide reasonable compensation for treatment within the scope of compensations that are not provided by health insurance, the government or a third party.
Research-based clinical literature (references)	1. N Engl J Med (2005)352(10);987-96<br>2. J Neuro-Oncology (2005)72;57-62<br>3. J Clin Oncol (2005)23(10);2372-7<br>4. Rosenberg SA. Progress in human tumour immunology and immunotherapy. Nature 2001; 411:380-4.<br>5. Weller M. Neuro Oncol. 2012 Nov 7.

2. Schedule

	Screening		Treatment period[1)]																Termination[2)]	Survival[3)]
**Visit**	1	2	3	4	5	6	7	8	9	10	11	12	13	14	15	16	17	18		
**Week**	Within -3	Within -2	0	1	2	3	4	5	6	8	10	12	14	16	18	20	22	24		Every 4 weeks
Written consent	✓																			
Assignment of screening number	✓																			
Demographic survey [4)]	✓																			
Investigation of medical history[5)]	✓																			
Investigation of medication history[6)]	✓		✓	✓	✓	✓	✓	✓	✓	✓	✓	✓	✓	✓	✓	✓	✓	✓	✓	
Vital signs[7)]	✓		✓	✓	✓	✓	✓	✓	✓	✓	✓	✓	✓	✓	✓	✓	✓	✓	✓	
ECG	✓																			
Physical examination[8)]	✓		✓	✓	✓	✓	✓	✓	✓	✓	✓	✓	✓	✓	✓	✓	✓	✓	✓	
Pregnancy test[9)]	✓																			
Clinical laboratory tests[10)]	✓		✓				✓			✓		✓		✓		✓		✓	✓	
QLQ-C30 (Ver 3.0)	✓		✓				✓			✓		✓		✓		✓		✓	✓	
KPS	✓		✓				✓			✓		✓		✓		✓		✓	✓	
Selection/exclusion criteria		✓																		
Assignment of registration number		✓																		
Blood collection[11)]		✓	✓		✓		✓		✓	✓	✓	✓	✓	✓	✓	✓	✓			
Administration of investigational drug[12)]			✓		✓		✓		✓	✓	✓	✓	✓	✓	✓	✓	✓			
Tumor evaluation[13)]	✓											✓						✓	✓	
Evaluation of dose-limiting toxicity [14)]					✓	✓	✓	✓	✓	✓										
Check for adverse events			✓	✓	✓	✓	✓	✓	✓	✓	✓	✓	✓	✓	✓	✓	✓	✓	✓	✓

1) Treatment period:	All visits shall be made within ± 3 days of the scheduled date. Due to the issue of test drug supply, the visit can be delayed for up to two weeks.
2) Termination:	A final visit for termination or discontinuation of the clinical trial shall be scheduled in the event that any of the withdrawal (drop-out) and termination criteria applies.
3) Survival:	After the clinical trial is terminated for a reason other than withdrawal of consent, the survival information on the subjects shall be collected once every 4 weeks in person or by phone after the termination visit.
4) Demographic Survey:	The subjects' initials, sex, age, height and weight shall be determined, and the BMI shall be calculated.
5) Investigation of medical history:	The onset of prior medical conditions within the past 5 years and current medical conditions shall be examined.
6) Investigation of medication history:	The subjects' anticancer therapy history shall be investigated through an inquiry (verbal or questionnaire) during the screening process, and as for the use of other types of pharmaceutical drugs, the history of taking drugs in the past 3 months and the current administration status (dosage and directions, duration of administration, etc.) shall be investigated. After registration, the subjects' medical history shall be examined at every visit and all of the drugs taken before the visit (administration of anticancer agent) shall be documented.
7) Vital signs:	The body temperature, pulse and blood pressure shall be checked.
8) Physical examination:	The subjects' external appearance, skin, head and neck, chest and lungs, heart, abdomen, urinary and reproductive systems, limbs, musculoskeletal system, nervous system, lymph nodes and other body organs shall be examined.
9) Pregnancy test:	For women of childbearing age, an HCG response test shall be performed using a urine sample.
10) Clinical laboratory tests	- Hematological test: RBC, hemoglobin, hematocrit, WBC, differential white blood cell, platelet, absolute neutrophil count (ANC)<br>- Hematobiochemical test: SGOT, SGPT, alkaline phosphatase, total protein, albumin, total bilirubin, cholesterol, uric acid, BUN,<br>creatinine, LDH, electrolytes (Na, K, and Cl)<br>- Autoimmune reaction test: RF (Rheumatoid Factor), ANA (Antinuclear Antibody), FT4, TSH, T3, CRP<br>- Urine test: Color, pH, specific gravity, protein, glucose<br><br>If there are test results obtained within four weeks prior to the date on which the written consent was obtained, the said results may be used instead.
11) Blood collection:	60ml shall be collected to produce the cellular therapy agent (investigational drug).
12) Administration of investigational drug:	The investigational drug shall be administered once every two weeks for a maximum of 24 doses.
13) Tumor evaluation:	Response of therapeutic effects shall be evaluated RECIST and MR image. Tumor evaluation must be performed within 7 days.
14) Evaluation of dose-limiting toxicity:	The first three subjects will be assessed for up to eight weeks from the test drug dose.

3. Background of the clinical trial

Glioma accounts for approx. 35% of all primary brain tumors, while glioblastoma accounts for around 40% of all glioma cases. Glioblastoma, which occurs commonly in men aged between 45 and 65, is the most aggressive malignant glioma and is very difficult to treat. Although glioblastomas form a mass in some cases, most exhibit invasive growth into the surrounding tissue, rendering treatment by a localized surgical procedure impossible.

With the introduction of nitrosoureas in the early 1970s, numerous clinical trials have been carried out with patients suffering from primary or recurrent malignant brain tumors in order to examine the therapeutic effects of anticancer agents. According to a meta-analysis, chemotherapy is recommended in combination with radiation therapy following surgery, but the 5-year survival rate is very low at under 2%. Thus, the purpose of treatment for glioblastoma, which has a grim prognosis as such, is to extend the survival period as much as possible and to manage the disease so that the patient's quality of life does not deteriorate while he/she is still alive.

Weller M. et al. (Neuro Oncol. 2012 Nov 7. [Epub ahead of print]) reviewed the abstracts on PubMed and American Society of Clinical Oncology from Jan. 2006 until Jan. 2012, and examined the clinical trials that used anticancer drugs such as nitrosoureas, which are the most common type of drug prescribed for recurrent glioblastoma patients, as well as temozolomide and bevacizumab, which have recently been developed and are now used regularly.

It has been reported that reoperation or reirradiation is attempted for some patients and there is increased toxicity from the anticancer drugs used afterwards, but the progression-free survival rate at 6 months is only 20~30%. The aim of this study is, therefore, to try a new type of immunotherapy in such cases where further treatment cannot be anticipated in the event of recurrence.

With respect to the immune function of the human body, basic research has been conducted on not only pathogens that enter the body from the outside, but also malignant tumors that arise from inside the body, and the findings of some of the studies are applied clinically. Based on the fact that many studies have shown that immune cells attack tumors, there have been a number of studies on prophylactic treatment using agents that specifically or nonspecifically enhance immunity with the involvement of lymphocytes and phagocytes. In addition, one of the most active fields of research today is to proliferate specific immune cells by cytokines in peripheral blood and to apply them to tumor immunity.

According to the study published in Anticancer Research 24: 1861-1872 (2004) by Ishikawa et al., the autologous natural killer (NK) cells are safe to use with partial effectiveness. As shown in the following table, autologous NK cells have no neurological toxicity and induce a partial response.

Table IV. *Responses and neurological toxicities of NK therapy.*

Case No.	Course of therapy	Total ($x10^9$)	dose (-fold)	Injected route	(*i.c.* %)	Response to the therapy	Neuro-logical toxicity	Posttreatment K.P.S. (from initial NK cell injection to outcome)
1	1	3.1	43.7	*i.v.*	(0)	NC	Grade 0	Dead(18 months)
2	1	3.3	43.6	*i.v.+i.c.*	(50)	PD	Grade 0	Dead (6 months)
3	1	0.6	11.3 #	*i.v.*	(0)	PR	Grade 0	50%
	2	3.5	52.5	*i.v.*	(0)	PD	Grade 0	40%
	3	6.5	90.1	*i.v.*	(0)	PD	Grade 0	Dead(11 months)
4	1	0.7	12.1 #	*i.v. +i.c.*	(51)	NC	Grade 0	10%
	2	2.5	42.6	*i.v.*	(0)	MR	Grade 0	10% (15 months)
5	1	5.1	52.7	*i.v.+i.c.*	(43)	PR	Grade I*	70%
	2	4.6	64.1	*i.v.+i.c.*	(50)	PR	Grade I*	70%
	3	4.0	54.9	*i.v.+i.c.*	(50)	PD	Grade 0	70% (12 months)
6	1	0.6	13.9	*i.v.+i.c.*	(65)	PD	Grade 0	Dead**
7	1	4.3	59.6	*i.v.+i.c.*	(13)	NC	Grade 0	Dead (4 months)
8	1	1.8	63.6	*i.v.+i.c.*	(50)	MR	Grade 0	40%
	2	1.7	23.8 #	*i.v.+i.c.*	(50)	PD	Grade 0	40% (5 months)
9	1	0.8	11.5	*i.v.*	(0)	NC	Grade 0	80%
	2	3.2	44.7	*i.v.+i.c.*	(50)	PD	Grade 0	80% (5 months)

# Data regarding cell expansion that proceeded with difficulty are included in the calculation.
* Headache, nausea and irritability.
** Another therapy was performed one month after the period of NK therapy.
i.v.: intravenous; *i.c.*: intracranial.

The cellular immunotherapy agent used in this study is the autologous killer cell (AKC), a type of autologous activated lymphocyte obtained by isolating lymphocytes from the peripheral blood and inducing selective proliferation of the lymphocytes, NK, NKT and T cells. Of these, the natural killer (NK) cells are the immune cells playing the primary function of AKC.

AKC is a cellular immunotherapy agent manufactured at CHA Bundang Medical Center's GMP facility and it will be applied to the human body for the first time as a medicinal product. AKC used in this study was cultured for around two weeks after adding a specific antigen and IL-2 to induce proliferation of the immune cells.

Although the safety of domestic and foreign autologous immune cell therapy agents has been reported by a number of clinical trials, the safety of AKC specifically produced by CHA Bundang Medical Center's GMP facility has yet to be directly established and thus, securing safety is most important in this clinical trial. In addition, tumor is typically treated by surgery, radiotherapy, or chemotherapy, but most malignant tumors are difficult to treat with the above therapies alone due to their anatomical location, which makes it essential to perform adjuvant therapy to improve the prognosis. Among the available methods for adjuvant therapy, adaptive immunotherapy by autologous activated immune cells is very effective. This method of therapy is easy to administer and associated with fewer adverse effects than conventional chemotherapy and thus is expected to help improve the prognosis of cancer.

4. Purpose of the clinical trial

The purpose of this clinical trial is to administer the autologous killer cell, the investigational drug, to patients with recurrent glioblastoma and monitor the safety and effectiveness of the immunotherapy.

5. Clinical trial period

48 months from the date of approval by the Ministry of Food and Drug Safety (MFDS) and the IRB (the duration of the clinical trial or the registration period may vary)

6. Medicinal products used in the clinical trial

6.1 Investigational drug

1) Name of the formulation: The Autologous Killer Cell
2) Properties and dosage form: A self-derived activated cell infusion solution filled in an opaque poly (ethylene)
3) Amount of active ingredient
Main ingredient: Autologous activated lymphocytes (more than 1 x 10^9 cells)

6.2 Investigational drug management and records

In consideration of the characteristics of the cell therapy agent, the test person designated by the test director in place of the management pharmacist must confirm in writing and sign the receipt and quantity of the test drug and manage it according to the storage conditions. The examiners designated by the examiners shall ensure that the test drug is administered only to the subjects according to the protocol and ensure that records are kept of all the drugs administered to each subject. Each dosage information of the test drug is recorded in the case report. The investigational drug in this clinical trial must be manufactured individually for each subject at CHA Bundang Medical Center's GMP facility to be supplied to the sub-investigator and be administered to the subjects within 24 hours after manufacture.
1) After producing the test drug, fill out the quality test form and call the person in charge of clinical trial to inform the delivery schedule, the examiner will check the schedule of dosing so that the test drug can be administered.
2) CHA Bundang Medical Center's GMP facility shall deliver the investigational drug to the sub-investigator, who shall then check the investigational drug and sign and record the date on the hand-over sheet.
3) Principal investigator shall check the investigational drug and administer it to the subjects.
4) administration to subject is complete, the transfer bag returns to the GMP representative. The representative will sign and date the return confirmation.

6.3 Production, packaging and labeling of the investigational drug

A single dose of the investigational drug shall be packaged individually in a transfer bag. It shall be labeled and delivered to the clinical trial institution with the related records.
The sub-investigator designated by the principal investigator shall be responsible for the acquisition, storage, management and return of the investigational drug. The packaging container for the investigational drug shall be have a label with the following information in Korean, and the investigational drug shall be supplied to the clinical trial institution after the packaging process. The sub-investigator designated by the principal investigator shall handle and store the delivered investigational drug in a safe and appropriate manner. The investigational drug shall be stored in a safe location that is accessible only to the investigators and designated personnel.
• Labeled as "Investigational Drug"
• The product's code name or the general name of the active ingredient
• Lot number and shelf life or re-examination date
• Storage method
• Name and address of the party that acquired approval for the clinical trial protocol
• Labeled "Cannot be used for purposes other than the clinical trial"
• Registration number

7. Subject selection criteria, exclusion criteria, target number of subjects, and calculation basis

7.1 Selection criteria

1) Individuals aged 18 years or older but under 70
2) Individuals for whom the standard therapy failed after being diagnosed with recurrent glioblastoma based

on an MRI scan
3) Individuals whose survival period is at least 3 months
4) Individuals with an appropriate bone marrow function
- Hemoglobin count > 10 g/dL
- Leukocyte count > 3,000/mm^3
- Absolute neutrophil count > 11,500/μL
- Platelet count > 75.000/mm^3
5) Individuals with appropriate liver and kidney functions
- Total bilirubin ≤ 1.5 times the normal upper limit specified by the testing agency
- AST, ALT ≤ 2.5 times the normal upper limit specified by the testing agency
- Alkaline phosphatase ≤ 1.5 times the normal upper limit specified by the testing agency
- Normal kidney function (serum creatinine ≤ normal upper limit specified by the testing agency)
6) Individuals who have obtained a sufficient explanation of the purpose and details of the clinical trial and the characteristics of the investigational drug from an investigator and who signed the consent form or had a legal guardian or representative sign the consent form prior to the beginning of the clinical trial
7) In the case of women, they must have been tested negative in a urine or blood test for pregnancy seven days before the beginning of the clinical trial and agreed to use an appropriate method of contraception during the clinical trial
8) Individuals who have not had any blood infection within the last 6 months
9) Individuals who have not received any other forms of immunotherapy

7.2 Exclusion criteria

1) An individual who is deemed to have a severe cardiopulmonary dysfunction by an investigator
2) An individual with immune deficiency or an autoimmune disease that may be exacerbated by immunotherapy (e.g. rheumatoid arthritis, systemic lupus erythematosus, vasculitis, multiple sclerosis, juvenile-onset insulin-dependent diabetes mellitus, etc.)
3) A carrier of chronic hepatitis B and/or C or who is HIV antibody positive
4) An individual with history of severe allergic reactions
5) An individual with uncontrolled hypertension or diabetes
6) A pregnant or lactating woman
7) An individual who is considered to be unfit for the clinical trial due to a serious medical or psychiatric condition
8) An individual with a major hemorrhagic disease not associated with cancer
9) An individual who has participated in another clinical trial within 4 weeks before the start of this clinical trial

7.3 Target number of subjects

The clinical trial shall be carried out on a total of 23 subjects with recurrent glioblastoma among the applicants who meet the selection criteria and do not fall under any of the exclusion criteria.

7.4 Basis for calculating the target number of subjects

As this clinical trial is an exploratory study, the number of subjects was not calculated based on statistical significance.

7.5 Random assignment

This is a single-arm trial, so randomization is not applicable.

7.6 Clinical trial design

This is a prospective, open, single-arm, investigator-initiated trial carried out for the purpose of observing the safety and effectiveness of AKC, a cellular immunotherapy agent, in treating patients with recurrent glioblastoma.

A relatively safe dose of 1×10^9 cells was set as a single dose based on the clinical data reported in the existing literature. However, to ensure the safety of the indications in a stepwise manner, a dose - limiting toxicity assessment was devised. In addition, it is safer and more efficient to administer a safe dose reported in literature and carefully review the response before conducting additional research, rather than carrying out a toxicity test using a dose that has already been verified, in case significant overall survival (OS) or progression-free survival (PFS) cannot be expected from a standard therapy for patients with recurrent glioblastoma. Thus, a single dose

of 1 x 10^9 cells shall be administered to the 3 subjects. After conformed safety, the single dose of 1 x 10^9 cells shall be administered to the 20 additional subjects and the safety and effectiveness shall be assessed thereafter. The safety assessment shall be performed by the DSMB.

However, dose safety was assessed for a total of 6 subjects by adding 3 patients when dose-limiting toxicity (DLT: Dose-Limiting Toxicity) was observed in one of three primary subjects at the initial dose level. If dose-limiting toxicity is observed in more than 2 out of 2 or more than 2 out of 6 people, the safety and efficacy is assessed in the same way by a 50% reduction from the initially set dose. When dose-limiting toxicity is observed in one or less than six patients, the clinical dose is assessed as a safety dose and a safety and efficacy assessment is performed by adding the patient to the same dose level. The dose-limiting toxicity assessment should be carried out until the 8th week of the fourth dose of the test, and if dose-limiting toxicity appears after the fifth dose, it is reflected in the corresponding dose safety assessment.

*Dose Limiting Toxicity: After administration of a defined dose, it is assessed to be severe (grade 3 or higher) toxicity according to CTCAE (version 4.0).

7.7 Basis for setting the dose

Ishikawa et al. carried out 16 courses of cellular immunotherapy to patients with malignant glioma, with a dose of 0.6 x 10^9~6.5 x 10^9 cells. One course consisted three injections in a week, with 1 x 10^9 cells administered per injection, and the assessment was performed four to five weeks after treatment. The clinical results showed little to no neurological toxicity or adverse events after 9 courses and excellent cancer-killing ability. Clinical effectiveness was achieved by administering large amounts of a high-purity cellular immunotherapy agent.

Sally et al., on the other hand, administered 1 x 10^8 ~ 3 x 10^9 cells by increasing the dose incrementally to patients with kidney cancer or melanoma, and assessed the safety over the course of a 4-week treatment period after one course (3 injections). The group administered 1 x 10^8, 3 x 10^8 cells experienced no adverse events, while only one out of the three subjects administered 1 x 10^9 cells experienced a Grade 1 fever, whereas the other two subjects exhibited a stable disease condition. Meanwhile, three subjects administered 3 x 10^9 cells reported adverse events ranging from Grade 1 to 4, but they all exhibited either a stable disease condition or minor response, without death.

The objective of this study is to assess the therapeutic safety and effectiveness of the cellular immunotherapy agent in question in patients with recurrent glioblastoma, and more than 1 x 10^9 cells were set as a single dose, as it was deemed to be a safe dose in reference to the aforementioned clinical trials.

7.8 Method and duration of administration

For administration of the test drug, 60 ml of blood is collected two weeks before. After the blood collection, incubate for about 2 weeks for in vitro proliferation and inject 100 mL of the autologous Killer Cell containing 100 mL or more of the finished product, the Autologous Killer Cell, for 30 minutes to 1 hour. A maximum of 24 doses can be administered once every two weeks and a tumor is assessed every 3 months using MRI.

If there is a problem with the production of the test drug, failure of culture or infection, or difficulty in supplying the test drug, the visit can be delayed for up to two weeks. If the test drug is delayed more than 2 times per cycle, it is excluded from the PP. Also, check the GMP quality control results of The Autologous Killer Cell. If the quality control test results for adventitious viruses are positive, the patient should be closely followed up, and appropriate medical procedures including the administration of antibiotics should be conducted and recorded and reported to the trial director.

7.9 Conservative treatment

If blood transfusion is necessary (e.g. platelet transfusion to keep the platelet count above 20,000/μL), it may be carried out based on the judgment of the investigator. In case of deep vein thrombosis or pulmonary embolism, heparin, warfarin, etc. may be used for treatment. Other conservative treatments shall be carried out at the discretion of the investigator and be documented in detail in the case records.

7.10 Drugs that can be used in combination

In this clinical trial, all kinds of drugs may be administered in combination. However, in the case of anticancer agents that can be administered, they shall be restricted to BCNU, ACNU, PCV therapy, and Nolvadex tab.

7.11 Drugs that cannot be used in combination

There are no drugs prohibited in this clinical trial except for cell therapy agents.

However, if it is deemed by the investigator or the doctor responsible for treating other medical conditions of the subjects that it is necessary to use drugs in combination to treat the subjects during the clinical trial period, the related information must be documented in the case records in detail.

7.12 Matters for observation and observation method

7.12.1 Clinical trial schedule

	Screening		Treatment period[1)]																Termination[2)]	Survival[3)]
**Visit**	1	2	3	4	5	6	7	8	9	10	11	12	13	14	15	16	17	18		
**Week**	Within -3	Within -2	0	1	2	3	4	5	6	8	10	12	14	16	18	20	22	24		Every 4 weeks
Written consent	✓																			
Assignment of screening number	✓																			
Demographic survey [4)]	✓																			
Investigation of medical history[5)]	✓																			
Investigation of medication history[6)]	✓		✓	✓	✓	✓	✓	✓	✓	✓	✓	✓	✓	✓	✓	✓	✓	✓	✓	
Vital signs[7)]	✓		✓	✓	✓	✓	✓	✓	✓	✓	✓	✓	✓	✓	✓	✓	✓	✓	✓	
ECG	✓																			
Physical examination[8)]	✓		✓	✓	✓	✓	✓	✓	✓	✓	✓	✓	✓	✓	✓	✓	✓	✓	✓	
Pregnancy test[9)]	✓																			
Clinical laboratory tests[10)]	✓		✓				✓			✓		✓		✓		✓		✓	✓	
QLQ-C30 (Ver 3.0)	✓		✓				✓			✓		✓		✓		✓		✓	✓	
KPS	✓		✓				✓			✓		✓		✓		✓		✓	✓	
Selection/exclusion criteria		✓																		
Assignment of registration number		✓																		
Blood collection[11)]		✓	✓		✓		✓		✓	✓	✓	✓	✓	✓	✓	✓	✓			
Administration of investigational drug[12)]			✓		✓		✓		✓	✓	✓	✓	✓	✓	✓	✓	✓			
Tumor evaluation[13)]	✓											✓						✓	✓	
Evaluation of dose-limiting toxicity [14)]					✓	✓	✓	✓	✓	✓										

Check for adverse events			✓	✓	✓	✓	✓	✓	✓	✓	✓	✓	✓	✓	✓	✓	✓	✓	✓	✓

1) Treatment period:	All visits shall be made within ± 3 days of the scheduled date. Due to the issue of test drug supply, the visit can be delayed for up to two weeks.
2) Termination:	A final visit for termination or discontinuation of the clinical trial shall be scheduled in the event that any of the withdrawal (drop-out) and termination criteria applies.
3) Survival:	After the clinical trial is terminated for a reason other than withdrawal of consent, the survival information on the subjects shall be collected once every 4 weeks in person or by phone after the termination visit.
4) Demographic Survey:	The subjects' initials, sex, age, height and weight shall be determined, and the BMI shall be calculated.
5) Investigation of medical history:	The onset of prior medical conditions within the past 5 years and current medical conditions shall be examined.
6) Investigation of medication history:	The subjects' anticancer therapy history shall be investigated through an inquiry (verbal or questionnaire) during the screening process, and as for the use of other types of pharmaceutical drugs, the history of taking drugs in the past 3 months and the current administration status (dosage and directions, duration of administration, etc.) shall be investigated. After registration, the subjects' medical history shall be examined at every visit and all of the drugs taken before the visit (administration of anticancer agent) shall be documented.
7) Vital signs:	The body temperature, pulse and blood pressure shall be checked.
8) Physical examination:	The subjects' external appearance, skin, head and neck, chest and lungs, heart, abdomen, urinary and reproductive systems, limbs, musculoskeletal system, nervous system, lymph nodes and other body organs shall be examined.
9) Pregnancy test:	For women of childbearing age, an HCG response test shall be performed using a urine sample.
10) Clinical laboratory tests	- Hematological test: RBC, hemoglobin, hematocrit, WBC, differential white blood cell, platelet, absolute neutrophil count (ANC)<br>- Hematobiochemical test: SGOT, SGPT, alkaline phosphatase, total protein, albumin, total bilirubin, cholesterol, uric acid, BUN, creatinine, LDH, electrolytes (Na, K, and Cl)<br>- Autoimmune reaction test: RF (Rheumatoid Factor), ANA (Antinuclear Antibody), FT4, TSH, T3, CRP<br>- Urine test: Color, pH, specific gravity, protein, glucose<br><br>If there are test results obtained within four weeks prior to the date on which the written consent was obtained, the said results may be used instead.
11) Blood collection:	60ml shall be collected to produce the cellular therapy agent (investigational drug).
12) Administration of investigational drug:	The investigational drug shall be administered once every two weeks for a maximum of 24 doses.
13) Tumor evaluation:	Response of therapeutic effects shall be evaluated RECIST and MR image. Tumor evaluation must be performed within 7 days.
14) Evaluation of dose-limiting toxicity:	The first three subjects will be assessed for up to eight weeks from the test drug dose.

7.12.2 Matters for observation

7.12.2.1 Written consent and demographic survey

Before initiating the clinical trial, explain the purpose and details of the trial to the subjects in detail and obtain their written consent. Assign a screening number to each of the subjects in the order that the consent was provided and obtain their demographic information.
Check the subjects' demographic information such as initials, sex, and age, measure their height and weight, and calculate the body mass index (BMI) when administering an anticancer drug.

7.12.2.2 Investigation of medical and medication history

Obtain detailed information on the subjects' medical and medication history through an inquiry (verbal or questionnaire) and by checking their prior medical records, and record the information. The information that must be obtained is as follows:
Document the onset (year of occurrence or year and month of occurrence) of prior medical conditions in the last 5 years and current medical conditions including surgery, allergies, etc. and write down the investigator's comments and opinions. With respect to glioblastoma, the medical history including the duration of the disease shall be examined in detail through the inquiry process.
As for the medication history examined during screening, all prior anticancer therapies must be examined through an inquiry. As for other types of drugs used, the history of taking drugs in the past 3 months and the current administration status (dosage and directions, duration of administration, etc.) shall be investigated.
After registration, the subjects' medical history shall be examined at every visit and all of the drugs taken before the visit (administration of anticancer agent) shall be documented. However, information on general infusions and infusion preparations for mixing needs not be documented.

7.12.2.3 Measurement of vital signs

The vital signs such as blood pressure, heart rate, and body temperature shall be measured at each visit before the test/examination scheduled for the subject.

7.12.2.4 Physical examination

The physical examination shall be carried out at every visit. This examination includes an examination of external appearance, skin, head and neck, chest and lungs, heart, abdomen, urinary and reproductive systems, limbs, musculoskeletal system, nervous system, lymph nodes and other body organs shall be examined.
Any significant findings discovered during the screening test shall be recorded in the section on physical examination in the case records. In case of discovering any significant matters in the physical findings that fall under the definition of "adverse event" after initiating the administration of the investigational drug, the information shall be recorded in the section on adverse events in the case records. However, if an undesirable physical symptom develops prior to initiating the administration of the investigational drug, it shall be recorded in addition to the section on current medical conditions.

7.12.2.5 Pregnancy test and clinical laboratory tests

At the screening, women of childbearing age shall be subject to an HCG response test using a urine sample to determine whether they are pregnant, except for women who have undergone a sterilization operation or who are in menopause (incl. menopause caused by surgery).
Clinical laboratory tests shall be carried out at Visit 3 and 7 during the screening process and at 1-month intervals thereafter until the final visit. On days when drug administration is scheduled, these tests shall be performed before the administration of the investigational drug. However, if there are test results obtained within four weeks prior to the date on which the written consent was obtained, the said results may be used instead.

Inspection matters include the following.

- Hematology: RBC, hemoglobin, hematocrit, WBC, differential white blood cell, platelet, absolute neutrophil count (ANC)
- Hematochemistry: SGOT, SGPT, alkaline phosphatase, total protein, albumin, total bilirubin, cholesterol, uric acid, BUN, creatinine, LDH, electrolytes (Na, K, CL)
- Immunologic: RF (rheumatoid factor), ANA (antinuclear antibody), FT4, TSH, T3, CRP
- Virus: HBs Ag, HBs ab, anti-HCV, anti-HIV
- Urinalysis: color, pH, specific gravity, protein, glucose

- Pregnancy reaction test: urine HCG

7.12.2.6 Tumor evaluation

Tumor evaluation shall be performed once at the screening and every three months thereafter by using a brain MRI scan. Brain MRI scans are carried out to acquire axial (T1-pre & post, T2, proton), coronal (T1-post), and sagittal (T1-post) images. However, tumor evaluation results obtained within four weeks prior to the date on which the written consent was obtained, the said results may be used instead for the screening process.
The tumor evaluation criteria are as follows, and the tumor measurement method will involve the use of RECIST (Version 1.1).
"Treatment response" refers to a case in which a "complete response" or a "partial response" among the following five cases.
The terms used in the tumor evaluation and the definitions thereof are as follows:

- Complete Response (CR): Disappearance of all target lesions. Any pathological lymph nodes (whether target or non-target) must have reduction in short axis to < 10mm
- Partial Response (PR): At least a 30% decrease in the sum of diameters of target lesions, taking as reference the baseline sum diameters
- Progressive Disease (PD): At least a 20% increase in the sum of diameters of target lesions, taking as reference the smallest sum on study. In addition to the relative increase of 20%, the sum must also demonstrate an absolute increase of at least 5 mm.
- Stable Disease (SD): Neither a partial response (PR) nor progressive disease (PD)
- Not evaluated (NE): Applicable only if none of the target lesions have been evaluated or are evaluable, or only a procedure was carried out on the lesions. Note: If the sum of the diameters meets the criteria for disease progression, disease progression in the target lesion response takes precedence over NE.

As for the evaluation of the immune reactions of the subjects, other than the tumor evaluation, T cell proliferation assay and ELISPOT shall be examined using the subjects' blood samples with skin test.

7.12.2.7 KPS, QLQ-C30 (ver 3.0) Korean

Implement the Karnofsky performance status (KPS) examination for neurological examination and QLQ-C30 (Ver 3.0) Korean to examine the subjects' health status. These tests shall be carried out at screening and once every month until the final visit.

7.12.2.8 Screening based on selection/exclusion criteria (subject suitability assessment)

Select patients who meet the selection criteria based on the medical history and basic survey data and conduct a screening test after obtaining their written consent. Carry out a final evaluation at Visit 2 to check whether the subjects conforming to the selection criteria and exclusion criteria have been chosen, based on a comprehensive review of the screening tests.

7.12.2.9 Investigational drug

The administration of the investigational drug shall be initiated at Visit 3 and terminated at Visit 18. Up to maximum 12 doses can be administered.

7.12.2.10 Dose-limiting toxicity assessment

The dose-limiting toxicity assessment should be conducted until 8th week (10th visit) of the fourth dose of the test. However, if dose-limiting toxicity appears after the 5th dose, the dose shall be evaluated in the safety evaluation.

7.12.2.11 Investigation into adverse events

Once the administration of the investigational drug is initiated, the subjects shall be instructed to voluntarily report any adverse events and the occurrence of adverse events shall be checked at every visit through an interview or inquiry (verbal or questionnaire). The adverse events shall be documented in the case records in detail in relation to the date of occurrence and disappearance, the severity and consequences of the adverse event, the causal relationship between the investigational drug and the measures taken in relation to the investigational drug, the name of the drug other than the investigational drug suspected to have caused the adverse event, the treatment of adverse events and other details. The adverse events shall be categorized according to the Common Terminology Criteria for Adverse Events (CTCAE) attached hereto, in principle. If

applying these standards is not appropriate, however, another general standard classification method shall be used for the evaluation.

7.12.3 Clinical trial method by visit

7.12.3.1 Visit 1 (within -3 weeks; screening)

The subjects who have been selected to participate in this study shall receive an explanation of the clinical trial and assessed in the following order:

① Acquisition of written consent
② Assignment of screening number
③ Demographic survey: Initials, sex, age, height, weight, BMI
④ Investigation of medical history: Prior medical conditions within the last 5 years and current medical conditions incl. surgical operations
⑤ Investigation of medication history: All types of anticancer treatments and other drugs administered within the last 3 months
⑥ Vital signs: Blood pressure, pulse, body temperature
⑦ ECG
⑧ Physical examination: External appearance, skin, head and neck, chest and lungs, heart, abdomen, urinary and reproductive systems, limbs, musculoskeletal system, nervous system, lymph nodes and other body organs
⑨ Pregnancy test (female subjects of childbearing age)
⑩ Clinical laboratory tests: Hematological test, hematochemical test, urine test (may be substituted by test results obtained within 4 weeks prior to the acquisition of the written consent)
⑪ KPS, QLQ-C30 (Ver 3.0) Korean examinations
⑫ Tumor evaluation (may be substituted by test results obtained within 4 weeks prior to the acquisition of the written consent)
⑬ Scheduling of next visit

7.12.3.2 Visit 2 (within -2 weeks)

The screening test results are checked to see whether the candidate conforms to the selection/exclusion criteria for registration as a subject in the clinical trial.

① Final assessment based on the selection/exclusion criteria
② Assignment of registration number
③ Blood collection for investigational drug production (cell preparation)
④ Scheduling of next visit

7.12.3.3 Visit 3 (0 week)

The investigational drug shall be administered for the first time during this visit, and the following types of assessment shall be carried out:

① Investigation of medication history
② Vital signs: Blood pressure, pulse, body temperature
③ Physical examination: External appearance, skin, head and neck, chest and lungs, heart, abdomen, urinary and reproductive systems, limbs, musculoskeletal system, nervous system, lymph nodes and other body organs
④ Clinical laboratory tests: Hematological test, hematobiochemical test, and urine test
⑤ KPS, QLQ-C30 (Ver 3.0) Korean examinations
⑥ Blood collection for investigational drug production (cell preparation)
⑦ Administration of the investigational drug
⑧ Check for adverse events (at every visit)

⑨ Scheduling of next visit

7.12.3.4 Visit 4 ~ Visit 18 (1 ~ 6 weeks, 8 ~ 24 weeks)
The investigational drug shall be administered during these visits, and the following types of assessment shall be carried out:

① Investigation of medication history (at every visit)

② Vital signs: Blood pressure, pulse, body temperature (at every visit)

③ Physical examination: External appearance, skin, head and neck, chest and lungs, heart, abdomen, urinary and reproductive systems, limbs, musculoskeletal system, nervous system, lymph nodes and other body organs (at every visit)

④ Clinical laboratory tests: Hematological test, hematobiochemical test, and urine test (at visits 7, 10, 12, 14, 16 and 18)

⑤ KPS, QLQ-C30 (Ver 3.0) Korean examinations (at visits 7, 10, 12, 14, 16 and 18)

⑥ Blood collection for investigational drug production (at visits 5, 7 and 9 to 17)

⑦ Administration of the investigational drug (at visits 5, 7 and 9 to 18)

⑧ Tumor evaluation (at visits 12 and 18)

⑨ Dose-limiting toxicity assessment (at visits 5 to 10)

⑩ Check for adverse events (at every visit)

7.12.3.5 Termination visit
Where <u>9.1 Subject withdrawal (drop-out) and trial termination criteria</u> apply, a final visit for termination or discontinuation of the clinical trial shall be scheduled, and the following types of assessment shall be carried out during the visit:

① Investigation of medication history

② Vital signs

③ Physical examination

④ Clinical laboratory tests

⑤ KPS, QLQ-C30 (Ver 3.0) Korean examinations

⑥ Tumor evaluation (only if it is at F/U that occurs every three months)

⑦ Check for adverse events

7.12.3.6 Visit for checking survival
After the clinical trial is terminated for a reason other than withdrawal of consent, the survival information on the subjects shall be collected once every 4 weeks in person or by phone after the termination visit.

① Survival information

② Tumor evaluation (only if it is at F/U that occurs every three months)

③ Check for adverse events

7.12.3.7 Additional visit
Additional visits may be scheduled at any time when it is deemed necessary by the investigator or when it is requested by the subject or his/her legal representative.

8. User precautions for the medicinal products used in the clinical trial
The investigational drug in this clinical trial will be applied to the human body for the first time, and the potential adverse events that are expected are follows:

1. Cold-like symptoms such as fever, chills, and headache
2. Anaphylaxis: Cold sweat, skin flare, urticaria, dyspnea, etc.
3. General caution: Use of the investigational drug by those other than the subject is strictly prohibited.
4. Administration to pregnant and lactating women, newborns, infants and young children: A safety and

effectiveness study has not been conducted on pregnant and lactating women, newborns, infants and young children

9. Withdrawal and termination criteria and criteria for exclusion from analysis

9.1 Subject withdrawal (drop-out) and trial termination criteria

For all of the subjects participating in the clinical trial, whether they have completed the trial or not shall be recorded. If the drug administration or observation has been discontinued, the reason shall be recorded. A subject may withdraw or be removed from the clinical trial for any of the following reasons:

① The subject disqualified based on the selection/exclusion criteria

② A serious adverse event has occurred to the subject or the subject has requested to discontinue due to an adverse event

③ The subject who is found to have a systemic disease that was not discovered in the screening test

④ The subject (or his/her guardian) withdrew his/her consent to participate in the clinical trial

⑤ A serious violation of the protocol is committed by the principal investigator, a sub-investigator, or the subject in question

⑥ The subject does not recover from an adverse event

⑦ The subject cannot be tracked

⑧ There is a problem in administering the investigational drug to the subject

⑨ The subject has taken a drug, etc. that may affect the results of the study without any directions from the doctor in charge during the administration period or follow-up observation period

⑩ It is deemed by the principal investigator or sub-investigator that the study is not proceeding in an appropriate manner

⑪ The administration of the investigational drug has been postponed or cancelled three consecutive times or more during the clinical trial period or postponed four times or more in total

9.2 Compliance in the clinical trial and handling a protocol violation

The principal investigator and sub-investigator of this clinical trial shall fully familiarize themselves with and observe this clinical trial protocol to prevent any violations thereof. For the purpose of complying with the investigational drug administration and test/examination schedules, the sub-investigator shall take the appropriate measures for the subjects to make an outpatient visit as scheduled and such measures may include notifying them in writing or by phone, for example. If an unavoidable violation of the protocol has occurred, it shall be dealt with as follows:

In the event of a critical violation of the protocol, the subject in question shall be excluded from analysis (excl. PP), in principle.

① Failure to obtain written consent

② Does not the selection criteria or falls under the exclusion criteria

As for other minor violations of the clinical trial protocol that are considered to have no effect on the analysis of the study results, the severity of the violation or extent of delay and the reason behind the violation/delay shall be accurately described. The investigator, monitor, and statistician shall examine its impact on the study and include the information in the PP analysis.

10. Effectiveness assessment criteria, assessment method and analysis method (statistical analysis method)

10.1 Assessment criteria

The variables for the safety and effectiveness assessment for assessing the results of the clinical trial are as follows:

10.1.1 Safety assessment variables

① Adverse reactions: Fever, subjective and/or objective symptoms, causal relationship with the

investigational drug, etc.

② Clinical laboratory tests

③ Vital signs and physical examination

10.1.2 Effectiveness assessment variables

① Progression-free survival

② Overall survival

③ Treatment response rate according to the tumor evaluation

④ QLQ-C30 (Ver 3.0) Korean

⑤ Karnofsky performance status (KPS)

10.2 Statistical analysis method

10.2.1 General principles for results analysis

The data obtained from the subjects through this clinical trial are largely divided into three groups: Safety, Full Analysis (FA) and Per-Protocol (PP). The main population of analysis in this clinical trial is the FA group, while additional analysis is performed on the PP group and safety is analyzed based on the Safety group. However, in the case of the FA group for the primary effectiveness assessment, the analysis group shall be redefined by setting forth the exclusion criteria for the subjects taking into consideration of the characteristics of the assessment variables. The results of all three groups shall be compared, and when the results differ among the groups, the results of each analysis method shall be presented with the reasons for the differences described in detail. All of the analyses shall be performed at a significance level of 5%.

Assignment to the FA Group:

Those who have been administered the investigational drug at least once after being registered as a subject in this clinical trial and had the variables for the effectiveness assessment measured at least once after the administration shall be included in the analysis. For the FA group, the intention-to-treat (ITT) shall be a fundamental principle.

However, the FA group for the primary effectiveness assessment, the following subjects shall additionally be excluded.

⑩ A subject who no longer meets the selection criteria or meets the exclusion criteria

⑪ A subject who has never been administered the drug used in the clinical trial after registration in the study

⑫ A subject without any measurement values for effectiveness assessment after registration in the study

Assignment to the PP Group:

The PP group is comprised of subjects included in the FA group that have completed the clinical trial in accordance with the clinical trial protocol. However, subjects falling under any of the following shall be excluded:

⑨ A subject whose investigational drug administration has been postponed or cancelled three consecutive times or more or four or more times in total during the clinical trial period

⑩ A subject who did not recover from an adverse event even after 4 weeks

⑪ A subject who dropped out or was removed from the clinical trial before fulfilling the duration specified in the protocol

⑬ A subject who no longer meets the selection criteria or meets the exclusion criteria

⑭ Any other cases that could be considered a serious violation of the protocol

Assignment to the Safety Group:

Those who have been administered the investigational drug at least once shall be included in the analysis.

Handling of missing values

If there is a missing value at any point or a missing value occurs due to a subject dropping out or being removed

before the completion of the clinical trial, the missing data shall be substituted using the Last Observation Carried Forward (LOCF) method.

Analysis point

Statistical analysis will be performed 12 months after the last patient is enrolled in this study.

10.2.2 Demographic data

In the case of continuous data, the descriptive statistics (mean, standard deviation, minimum value, maximum value, etc.) shall be presented according to the time of visit, while in the case of categorical data, the frequency and ratio for each category shall be presented.

10.2.3 Safety data

10.2.3.1 Adverse events

In this clinical trial, adverse events shall be classified into 1) adverse events prior to administration of the investigational drug (i.e. underlying symptoms and signs) and 2) treatment emergent adverse events (TEAE). TEAE shall be defined as ① an adverse reaction that occurred for the first time after the administration of the investigational drug and ② an adverse event in which the severity of underlying symptoms and signs that have occurred before the administration of the investigational drug has worsened after the administration. In the analysis of the adverse events, TEAEs shall be analyzed and all of the adverse events shall be presented as a list.

In case of multiple occurrences of the same adverse event to a subject, the event shall be regarded as a single adverse event. If the severity of the same adverse event differs, the worst severity shall be noted. If the causality of the same TEAE in a subject is different, the causality that is most relevant to the investigational drug shall be noted.

The percentage of subjects who experienced an adverse event and the 95% confidence interval in relation thereto shall be presented. All of the adverse events that have occurred shall be documented according to the level of severity. The adverse events and serious adverse events associated with the investigational drug shall also be documented.

10.2.3.2 Clinical laboratory tests

In the case of continuous data such as the hematological or hematobiochemical test results, the descriptive statistics (mean, standard deviation, minimum value, maximum value, etc.) shall be presented, whereas in the case of categorical data such as the urine test and 12-lead ECG results, the frequency and ratio for each category shall be presented.

For continuous variables, a paired t-test or Wilcoxon signed-rank test shall be performed on the differences between before and after the administration of the investigational drug to examine the differences between the visits. McNemar's test shall be carried out for categorical variables. In addition, clinically significant non-normalized rates compared to before treatment shall be presented.

10.2.3.3 Vital signs and physical examination

The descriptive statistics for vital signs (blood pressure, pulse, and body temperature) shall be presented, and a shift table indicating the changes between before and after the administration of the investigational drug with respect to the normal and abnormal values measured in the physical examination shall be presented. The before and after differences shall be analyzed by a paired t-test or Wilcoxon signed-rank test for continuous variables and the McNemar's test for categorical variables.

10.2.4 Effectiveness data

Progression-free survival (PFS)

PFS shall be estimated by the Kaplan-Meier method, and the median survival time shall be calculated.

Response rate

The degree and percentage of the subjects who showed a response to treatment shall be presented, with the 95% confidence interval indicated.

Overall survival (OS)

OS shall be estimated by the Kaplan-Meier method, and the median survival time shall be calculated. OS shall

be the period between the date on which the individual was registered as a subject of this clinical trial and assigned a number and the date on which he/she died, irrespective of the cause.

QLQ-C30 (Ver 3.0) Korean
The measurements for each item shall be compared in order to determine the level, and the changes according to the progress of treatment shall be analyzed. The descriptive statistics (mean, standard deviation, median, minimum value, and maximum value) shall be presented with respect to the QLQ-C30 (Ver 3.0) Korean evaluation scores and be tested using an independent t-test or Wilcoxon's rank sum test depending on the presence of normality. As for making a comparison between before and after the administration of the investigational drug, a paired t-test or Wilcoxon's signed-rank test shall be performed.

Karnofsky performance status (KPS)
The measurements for each item shall be compared in order to determine the level, and the changes according to the progress of treatment shall be analyzed. The descriptive statistics (mean, standard deviation, median, minimum value, and maximum value) shall be presented with respect to the KPS evaluation scores and be tested using an independent t-test or Wilcoxon's rank sum test depending on the presence of normality. As for making a comparison between before and after the administration of the investigational drug, a paired t-test or Wilcoxon's signed-rank test shall be performed.

11. Safety assessment method and criteria and reporting method incl. adverse events

11.1 Terms related to safety and definitions thereof

1) Adverse Event (AE)
Any unfavorable and unintended sign (e.g. an abnormal laboratory finding), symptom, or disease temporally associated with the use of a medicinal product, whether or not considered related to the medicinal product

2) Adverse Drug Reaction (ADR)
All noxious and unintended responses to a medicinal product related to any dose, with a reasonable possibility that there is a causal relationship between a medicinal product and an adverse event

3) Serious and Unexpected Adverse Drug Reaction
An unexpected adverse drug reaction is one where the nature or severity of which is not consistent with the applicable product information (e.g. Investigator's Brochure or attached documents for the medicinal product) and expedited reporting is required in the event of a serious and unexpected adverse drug reaction.

4) Serious AE/ADR
A serious adverse event or adverse drug reaction occurring at any arbitrary dose of the medicinal product used in the clinical trial that falls under any of the following:

① results in death during the clinical trial period;
② is life-threatening (the patient was at risk of death at the time of the event/reaction);
③ results in persistent or significant disability or incapacity;
④ requires inpatient hospitalization or prolongation of existing hospitalization
⑤ leads to a congenital anomaly/birth defect;
⑥ any other medically important situations.

Upon occurrence of a situation that is deemed to cause a serious effect on the patient's health condition and well-being, even if it does not fall under any the above-listed situations, a decision shall be made on whether to consider it as a serious AE/ADR, based on the medical judgment of the doctor in charge and relevant experts, and an appropriate measure shall be taken accordingly.

11.2 Assessment method

1) An AE is a symptom that has not been observed prior to participation in the clinical trial but newly arises during administration of the investigational drug. Such includes unintended signs (incl. an abnormal laboratory finding), which does not necessarily have to have a causal relationship with

this treatment, as well as symptoms, or temporary phenomena related to the use of the investigational drug.

2) Any untoward clinical signs and symptoms that occur prior to the administration of the investigational drug after the screening process shall be recorded in the current medical history, but not recorded as AE.
3) If a subject becomes pregnant during the clinical trial period, it will not be regarded as a serious AE/ADR, but she shall drop out from the clinical trial. The information on the subject and her fetus shall be tracked and reported until delivery.
4) Any and all phenomena (signs, symptoms, onset, duration, etc.) expected as an AE from the investigational drug must be recorded in the AE report. Those that are not recorded shall be classified as subjective symptoms.
5) The degree of the adverse reaction shall be assessed by the sub-investigator in reference to the assessment criteria [CTCAE] according to severity of the symptoms.
6) The causal relationship with the investigational drug shall be assessed by the doctor in charge by classifying it into one of the six stages according to the assessment criteria (see Section 11.4).

11.3 Assessment criteria

11.3.1 Fever

For fevers, the body temperature shall be measured and it shall be classified according to the following assessment criteria:

Grade level	Grade 0	Grade 1	Grade 2	Grade 3	Grade 4
Fever (°C)	none	< 38.7	38.7- 40	> 40.0	Fever with hypotension

11.3.2 Subjective and objective symptoms

The degree of subjective and objective symptoms shall be assessed by classifying them according to the criteria proposed for abnormalities in the examined items (CTCAE; Version 4.0) through a medical examination by the sub-investigator. If the criteria are not appropriate, the three-step classification method such as Spilker and the following general standard classification method will be applied. In order to prevent confusion in recording, classification of degree of adverse event shall be unified as follows:

General classification method	None (0)	Mild (1)	Moderate (2)	Severe (3)		
WHO	Grade 0	Grade 1	Grade 2	Grade 3	Grade 4	
Spilker's classification method	None	Mild	Moderate	Severe		
CTCAE 5-step classification method	None	Mild	Moderate	Severe	Life-threatening consequence	Death

- 5 steps of WHO classification method
  - Grade 0: States that can perform normal activities without any constraint
  - Grade 1: Walking and work can be done, but physically severe activity is limited
  - Grade 2: Walking and self-protection are possible, but more than 50% of awake time cannot perform work
  - Grade 3: Only limited self-protection is possible, and more than 50% of the awake time depends on bed or chair
  - Grade 4: State that is unable to self-protect and is totally dependent on bad or chair

- 3 steps of the Spilker's classification method

Mild (1): A state in which treatment is not necessary and the subject's normal life is not significantly disturbed
Moderate (2): The subject's normal life may be significantly impaired, and treatment may be necessary, but the condition is recovered after treatment
Severe (3): Due to severe adverse events, a high degree of treatment is required, and the condition of aftereffects remains

- 3 steps of the general classification method
  Mild (1): Adverse events that can be easily tolerated
  Moderate (2): Adverse events that significantly affect daily life
  Severe (3): Adverse events that cannot be normal daily life

11.4 Causal relationship with the investigational drug

In the event of an AE, the relationship with the investigational drug shall be classified as follows by the investigator, and the comments and opinions of the sub-investigator shall be attached, if necessary.

① Definitely related
- There is evidence that the drug has been administered;
- The AE is most plausibly explained by the administration of the drug in question than any other reasons;
- The AE disappears once the drug administration is discontinued;
- The rechallenge (performed only when possible) result is positive;
- The AE is consistent with the information already known about the drug or a similar line of drugs.

② Probably related
- There is evidence that the drug has been administered;
- The drug administration and the AE have a reasonable time sequence;
- The AE is more plausibly explained by the administration of the drug in question than other reasons;
- The AE disappears once the drug administration is discontinued.

③ Possibly related
- There is evidence that the drug has been administered;
- The drug administration and the AE have a reasonable time sequence;
- The AE is deemed to have possibly caused by the drug in question with the same likelihood as other potential causes;
- The AE disappears once the drug administration is discontinued.

④ Probably not related
- There is evidence that the drug has been administered;
- There is a more plausible cause behind the AE;
- The result of discontinuing the drug administration (if discontinued) is negative or ambiguous.

⑤ Definitely not related
- There is no evidence that the drug has been administered;
- The AE is most plausibly explained by another reason;
- The AE does not disappear as a result of discontinuing the drug administration (if discontinued).

⑥ Unknown

11.5 Reporting method

11.5.1 Reporting adverse events

1) The principal investigator shall educate the sub-investigator, subjects and/or their guardians on all potential AEs that may occur after the administration of the investigational drug and instruct them to report any and all phenomena they experience after the administration.
2) The type, onset, degree, treatment measure, medication, progress, causal relationship with the

investigational drug, etc. of any systemic or clinicopathologic symptoms occurring after the use of the investigational drug shall be recorded and retained in accordance with the clinical trial management standards by documenting the information in the case records.

3) The principal investigator shall describe and assess all the symptoms that occurred during the clinical trial period when reporting the clinical trial results. In the event of a serious adverse drug reaction (ADR) during the trial, it shall be reported to the IRB so that the decision on whether to continue or discontinue the trial may be made. Where expedited reporting is necessary, the Minister of Food and Drug Safety shall be reported within the period set forth as follows:

4) A serious ADR refers to a case in which the causal relationship with the investigational drug cannot be ruled out and it may be any one of the following cases:

① results in death during the clinical trial period;

② is life-threatening (the patient was at risk of death at the time of the event/reaction);

③ results in persistent or significant disability or incapacity;

④ requires inpatient hospitalization or prolongation of existing hospitalization

⑤ leads to a congenital anomaly/birth defect;

⑥ any other medically important situations.

5) Additional safety information shall be reported periodically until the ADR ceases to occur (e.g. the ADR disappears or it is impossible to conduct a follow-up examination).

6) The principal investigator shall carry out the clinical trial in conformance with the Declaration of Helsinki and KGCP.

11.5.1.1 Expedited reporting

1) Purpose

The purpose of expedited reporting is to ensure that the Ministry of Food and Drug Safety (MFDS), investigators, and other related parties are aware of the new and important information about serious adverse drug reactions.

2) Matters requiring expedited reporting

① Single cases of serious and unexpected ADR

② Where prompt communication with the MFDS is necessary

Appropriate medical and scientific judgment should be applied for each situation. In general, information that might materially influence the benefit-risk assessment of a medicinal product or that would be sufficient to consider changes in medicinal product administration or in the overall conduct of a clinical investigation represents such situations. Examples include:

a). For an "expected," serious ADR, an increase in the rate of occurrence which is judged to be clinically important.

b). A significant hazard to the patient population, such as lack of efficacy with a medicinal product used in treating life-threatening disease.

c). A major safety finding from a newly completed animal study (such as carcinogenicity).

3) Reporting time frames

In the event of a serious and unexpected ADR during the clinical trial, the principal investigator shall report to the Clinical Trials Review Committee to decide whether to continue or discontinue the trial, and report it promptly to the Food and Drug Administration within the period specified in each of the following items:

① Unexpected ADRs that are fatal or life-threatening shall be reported promptly. The applicant shall report to the Food and Drug Administration as soon as possible through telephone, fax and documentation within 7 days of the initial report and submit the complete report within 15 days from the first reported date.

② All other serious and unexpected ADRs shall be reported by the principal investigator within 15 days after receiving a report thereon or becoming aware of it.

4) Minimum criteria for initial reporting

Information for the final description and evaluation of an initial report may not be available within the

aforementioned time frames for reporting. However, an initial report must be submitted within the prescribed time above and must include the following categories of information:

① Subject in whom the ADR occurred;
② Suspected drug;
③ Information provider;
④ Serious and unexpected ADR.

Detailed follow-up information shall be obtained and submitted for use.

5) Reporting method
For expedited reporting of ADRs, the Serious Adverse Drug Reaction Report Form of the Ministry of Food and Drug Safety shall be used. The utmost effort shall be made to obtain as much information as possible, even if all the information is not available at the time of expedited reporting.

11.6 Measures to be taken for serious ADRs

During the clinical trial period, the principal investigator and the sub-investigator must make every effort to ensure the safety of the subjects. In the event of a serious ADR, the trial shall be suspended to take prompt and appropriate measures so as to minimize the ADR.
The duties of each person in charge in the event of a serious ADR during the clinical trial are as follows:

1) Duties of the principal investigator
The principal investigator shall, in the event of a serious ADR during the clinical trial, immediately report to IRB, and entirely or partially suspend the clinical trial involving the investigational drug in question until further instructions are given.

2) Duties of the sub-investigator
The sub-investigator shall, in the event of a serious ADR during the clinical trial, report it immediately to the principal investigator.

3) Duties of the IRB
The IRB shall, in the event of a serious ADR, have the principal investigator take necessary measures such as commanding that the clinical trial be suspended entirely or partially.

4) Duties of the sponsor
If the sponsor receives a severe and unexpected adverse event report from investigator or sub-investigator, a copy of report should be immediately submitted to the Commissioner of the Food and Drug Administration. In addition, if a clinical trial has been conducted in many of institutions, it should be notified immediately.

12. Written consent, compensation agreement, and post-trial medical examination and treatment of the subjects

12.1 Consent form and written explanations

The principal investigator or sub-investigator shall provide a sufficient explanation of the details of the study, effects of the investigated drug, and possible adverse events to the subjects (or their legal representatives) before initiating the study and obtain written consent from the subjects. The investigators shall keep the original copy of the signed consent form as part of the clinical trial records and provide a photocopy of the signed consent form and written explanations to the subjects (or their legal representatives).
See Appendix 2 Consent Form and Explanations for the Subjects

12.2 Agreement on compensation

See Appendix 3 Agreement on Victim Compensation

12.3 Post- trial medical examination and treatment of the subjects

Subjects dropping out of the clinical trial or unresponsive to the investigational drug shall be instructed to seek other types of appropriate treatment. Subjects who have complete the clinical trial shall be instructed to receive medical examination anytime, according to the instructions of the doctor in charge, in preparation for

unexpected and delayed adverse events.

13. Measures for ensuring the safety of the subjects

13.1 Clinical trial institution

The head of the clinical trial institution shall have the clinical laboratory, equipment, and specialized manpower necessary for carrying out each phase of the clinical trial in question and strive for perfection when making the preparations to ensure the proper execution of the clinical trial.

13.2 Approval of and amendments to the clinical trial protocol

In case of planning to obtain approval of the clinical trial protocol or amend the approved clinical trial protocol, IRB's approval for each phase of the clinical trial or the amended protocol shall be obtained, and the approval of the Minister of Food and Drug Safety shall be obtained, if necessary. Subjects cannot be enrolled in the clinical trial for participation prior to approval.

13.3 Familiarization of the clinical trial protocol

This clinical trial has been prepared with the rights and welfare of the subjects in mind, based on the Declaration of Helsinki and KGCP. As such, the principal investigator and sub-investigators shall accurately analyze and familiarize themselves with the clinical trial protocol and actively respond to the problems concerning the subjects in the clinical trial.

13.4 Consent to participate in the clinical trial

Before initiating the clinical trial, the subjects shall be explained of all matters in relation to the details of the clinical trial, effects of the investigational drug, adverse events, and safety, and their voluntary consent to participate in the study shall be obtained.

13.5 Accurate selection of subjects

Prior to the clinical trial, the suitability of the subjects shall be thoroughly assessed through a sufficient interview and tests/examinations.

13.6 Checking the progress of the clinical trial

The principal investigator shall periodically report to the sponsor the adverse events, progress of the trial, situation, results, etc., and the clinical trial sponsor shall periodically review the progress of the clinical trial.

13.7 Monitoring of the clinical trial institution

Monitoring shall be performed to check whether the rights and welfare of the subjects are protected, the data reported in relation to the clinical trial are accurate, complete and verifiable in comparison with the evidentiary documents, and whether the clinical trial is carried out in accordance with the approved protocol, Clinical Trial Management Standards for Medicinal Products and Subparagraph 8 Clause e) of the Enforcement Rule.
Monitoring of the clinical trial shall be performed by a monitor requested to perform the monitoring work through regular visits to the clinical trial institution and by phone. During a visit, the monitor shall check the original copies of the records on the subjects, drug management records, and records retention (research file). Also, the monitor shall review the progress of the clinical trial and speak to the investigators in case of a problem. The appropriate time frame for these visits shall be scheduled in a distributed manner through consultation between the investigators and the monitor. The investigators shall also make source documents (hospital or patient charts, records of the laboratory test results, appointment records, etc.) of the subjects available to the monitor so that the data and information entered into the case records can be checked, as defined in the Clinical Trial Management Standards for Medicinal Products.

13.8 Confidentiality

Any records with which the subjects can be identified shall be kept confidential, even when publishing the results of the clinical trial. The specific details in relation to confidentiality are as follows:
The monitors and inspectors concerned in this clinical trial may view the records of the subjects
for the purpose of monitoring and checking the clinical trial and managing the progress thereof. The investigators shall be aware that, with the conclusion of the agreement on this trial, the sponsor, monitors and

inspectors from the institution commissioning this clinical trial may review or photocopy the subjects' charts and case records in order to verify their content. Such information must be kept confidential and the necessary facility and management standards must be in place to ensure confidentiality.
On the other hand, all documents related to the clinical trial such as case records shall be recorded and identified with the subject identification codes (generally the subjects' initials), not their names.

13.9 Measures taken in the event of an adverse event

In the event of an adverse event, the subject in question shall receive the necessary examination and treatment from the doctor in charge. In case of a serious adverse drug reaction, the clinical trial shall be suspended and prompt and appropriate measures shall be taken according to the prescribed matters.

14. Matters necessary for conducting the clinical trial in a safe and scientific way

There shall be a separate location for storing various materials and records related to the execution of the clinical trial, and the security thereof shall be well-maintained. After completing the results report, a person responsible for retaining the related materials and records shall be designated so that they can be retained for three years from the end date of the clinical trial.

15. References

1) Standards of care for treatment of recurrent glioblastoma--are we there yet? Neuro Oncol. 2012 Nov 7. [Epub ahead of print]

2) Radiotherapy plus concomitant and adjuvant temozolomide for glioblastoma. N Engl J Med. 2005 Mar 10; 352(10): 987-96.

3) Human interferon beta, nimustine hydrochloride, and radiation therapy in the treatment of newly diagnosed malignant astrocytomas. Journal of Neuro-Oncology. 2005;72: 57-62.

4) Randomized phase II study of temozolomide and radiotherapy compared with radiotherapy alone in newly diagnosed glioblastoma multiforme. J Clin Oncol. 2005 Apr 1; 23(10): 2372-7.

4) Central nervous system cancers: Clinical practice guidelines Vol. 2, 2005. National Comprehensive Cancer Network.

5) Health-related quality of life in patients with glioblastoma: a randomized controlled trial. Lancet Oncology 2005; 6: 937-44.

6) Designs for group sequential tests. Fleming, T.R., Harrington, D.P., & O'Brien, P.C. Controlled Clinical Trials 1984;. 5:348-361.

7) A multiple testing procedure for clinical trials. O'Brien, P.C., & Fleming, T.R. Biometrics. 1979; 35:549-556.

8) Autologous Natural Killer Cell Therapy for Human Recurrent Malignant Glioma. Anticancer Res.2004; 24:1861-1872.

9) Infusion of the allogeneic cell line NK-92 in patients with advanced renal cell cancer or melanoma: a phase 1 trial. 2008;10, No 6: 625-632

10) Survival of Patients with Recurrent Malignant Glioma Treated with Temozolomide. Drug R&D 2003;4(5) :285-291

11) The clinical efficacy of combination of docetaxel and temozolomide in previously treated patients with stage IV melanoma research.2010 ;Vol 20 :43-47

16. Appendix

Appendix 1. Clinical Trial Sign Lock
Appendix 2. Subject consent manual and written consent
Appendix 3. Covenant on Victims Compensation
Appendix 4. RECIST (version 1.1)
Appendix 5. KPS (Karnofsky Performance Status)
Appendix 6. QLQ-C30 (version 3.0) Korean

4. Final protocol (version 8.0)

Clinical Trial Protocol

Investigator Clinical Phase 1/2 Study of Immunotherapy Using
Autologous Killer Cell in Patients with Recurrent Glioblastoma

Protocol No.: The Autologous Killer Cell
Version 8.0_2018.07.12

Confidentiality Statement
The information contained in this clinical trial protocol is solely intended as a guidance for the head of the testing agency, principal investigator, sub-investigators, managing pharmacist, related administrative agencies, and Institutional Review Board (IRB) of the clinical trial institution.<br>This protocol may not be disclosed to any party unrelated to the clinical trial without obtaining written consent, except in cases where it is necessary to obtain written consent from the subjects.

Contents

Investigator’s Signature....48
1. Summary....50
2. Schedule....56
3. Background of the clinical trial....61
4. Purpose of the clinical trial....62
5. Clinical trial period....62
6. Medicinal products used in the clinical trial....62
6.1 Investigational drug....62
6.2 Investigational drug management and records....63
6.3 Production, packaging and labeling of the investigational drug....63
6.4 Investigational drug manager....63
7. Subject selection criteria, exclusion criteria, target number of subjects, and calculation basis....63
7.1 Selection criteria....63
7.2 Exclusion criteria....64
7.3 Target number of subjects....64
7.4 Basis for calculating the target number of subjects....64
7.5 Random assignment....64
7.6 Clinical trial design....64
7.7 Basis for setting the dose....65
7.8 Method and duration of administration....65
7.9 Conservative treatment....65
7.10 Drugs that can be used in combination....66
7.11 Drugs that cannot be used in combination....66
7.12 Matters for observation and observation method....66
7.12.1.1 Written consent and demographic survey....66
7.12.1.2 Investigation of medical and medication history....66
7.12.1.3 Measurement of vital signs....66
7.12.1.4 Physical examination....66
7.12.1.5 Pregnancy test and clinical laboratory tests....67
7.12.1.6 Tumor evaluation....67
7.12.1.7 KPS, QLQ-C30 (ver 3.0) Korean....67
7.12.1.8 Screening based on selection/exclusion criteria (subject suitability assessment)....67
7.12.1.9 Investigational drug....67
7.12.1.10 Administration of anticancer agent....68
7.12.1.11 Investigation into adverse events....68
7.12.2 Clinical trial method by visit....68
8. User precautions for the medicinal products used in the clinical trial....70
9. Withdrawal and termination criteria and criteria for exclusion from analysis....70
9.1 Subject withdrawal (drop-out) and trial termination criteria....70
9.2 Compliance in the clinical trial and handling a protocol violation....72
10. Effectiveness assessment criteria, assessment method and analysis method (statistical analysis method)....72
10.1 Assessment criteria....72
10.1.1 Safety assessment variables....72
10.1.2 Effectiveness assessment variables....72
10.2 Statistical analysis method....72
10.2.1 General principles for results analysis....72
10.2.3 Safety data....74
10.2.4 Effectiveness data....75
11. Safety assessment method and criteria and reporting method incl. adverse events....75
11.1 Terms related to safety and definitions thereof....75

11.2 Assessment method 76
11.3 Assessment criteria 76
11.3.1 Fever 76
11.3.2 Subjective and objective symptoms 77
11.4 Causal relationship with the investigational drug 77
11.5 Reporting method 77
11.5.1 Reporting adverse events 77
11.6 Measures to be taken for serious ADRs 79
12. Written consent, compensation agreement, and post-trial medical examination and treatment of the subjects 79
12.1 Consent form and written explanations 80
12.2 Agreement on compensation 80
12.3 Post- trial medical examination and treatment of the subjects 80
13. Measures for ensuring the safety of the subjects 80
13.1 Clinical trial institution 80
13.2 Approval of and amendments to the clinical trial protocol 80
13.3 Familiarization of the clinical trial protocol 80
13.4 Consent to participate in the clinical trial 80
13.5 Accurate selection of subjects 80
13.6 Checking the progress of the clinical trial 80
13.7 Monitoring of the clinical trial institution 81
13.8 Confidentiality 81
13.9 Measures taken in the event of an adverse event 81
13.10 Data and Safety Monitoring Board (DSMB) 81
14. Matters necessary for conducting the clinical trial in a safe and scientific way 81
15. References 81

Investigator's Signature

I have
I have read and understood all the content of this protocol.
I also agree to carry out the clinical trial in accordance with the regulatory procedures and other matters specified in this clinical trial protocol as well as other relevant regulations and clinical trial management standards.

Principal Investigator	CHO, Kyung Gi
	(NAME/SIGNITURE) (YYYY/MM/DD)

Institution/Department	CHA Bundang Medical Center (CBMC) of CHA University / Department of Neurosurgery

Acronyms

Acronym	Definition
ADE	Adverse Device Effect
ADR	Adverse Drug Reaction
AE	Adverse Event
BUN	Blood Urea Nitrogen
CRF	Case Report Form
CRO	Contract Research Organization
CTCAE	Common Terminology Criteria of Adverse Event
DLT	Dose Limiting Toxicity
FAS	Full Analysis Set
IRB	Institutional Review Board
ITT	Intent To Treat
KGCP	Korea Good Clinical Practice
KPS	Karnofsky Performance Status
OS	Overall Survival
PFS	Progression-free survival
PP	Per-Protocol
PT	Preferred Term
QOL	Quality Of Life
RBC	Red Blood Cell
SAE	Serious Adverse Event
WBC	White Blood Cell

1. Summary

Clinical trial title	Investigator Clinical Phase 1/2 Study of Immunotherapy Using Autologous Killer Cell in Patients with Recurrent Glioblastoma
Implementing agency	CHA Bundang Medical Center (CBMC) of CHA University, 59, Yatap-ro, Bundang-gu, Seongnam-si, Gyeonggi-do, Republic of Korea
Principal investigator	CHO Kyung Gi, Professor of Neurosurgery, CHA Bundang Medical Center (CBMC) of CHA University / 031-780-5263
Co-investigators	KIM Joo Pyung, Professor of Neurosurgery, CHA Bundang Medical Center (CBMC) of CHA University / 031-780-5262<br>LIM Jae Joon, Assistant Professor of Neurosurgery, CHA Bundang Medical Center (CBMC) of CHA University / 010-8625-1829
Purpose	To administer the autologous killer cell, the investigational drug, to patients with recurrent glioblastoma and monitor the safety and effectiveness of the immunotherapy
Background of research	Glioma accounts for approx. 35% of all primary brain tumors, while glioblastoma accounts for around 40% of all glioma cases. Glioblastoma, which occurs commonly in men aged between 45 and 65, is the most aggressive malignant glioma and is very difficult to treat. Although glioblastomas form a mass in some cases, most exhibit invasive growth into the surrounding tissue, rendering treatment by a localized surgical procedure impossible.<br>With the introduction of nitrosoureas in the early 1970s, numerous clinical trials have been carried out with patients suffering from primary or recurrent malignant brain tumors in order to examine the therapeutic effects of anticancer agents. According to a meta-analysis, chemotherapy is recommended in combination with radiation therapy following surgery, but the 5-year survival rate is very low at under 2%. Thus, the purpose of treatment for glioblastoma, which has a grim prognosis as such, is to extend the survival period as much as possible and to manage the disease so that the patient's quality of life does not deteriorate while he/she is still alive.<br>Weller M. et al. (Neuro Oncol. 2012 Nov 7. [Epub ahead of print]) reviewed the abstracts on PubMed and American Society of Clinical Oncology from Jan. 2006 until Jan. 2012, and examined the clinical trials that used anticancer drugs such as nitrosoureas, which are the most common type of drug prescribed for recurrent glioblastoma patients, as well as temozolomide and bevacizumab, which have recently been developed and are now used regularly.<br>It has been reported that reoperation or reirradiation is attempted for some patients and there is increased toxicity from the anticancer drugs used afterwards, but the progression-free survival rate at 6 months is only 20~30%. The aim of this study is, therefore, to try a new type of immunotherapy in such cases where further treatment cannot be anticipated in the event of recurrence.<br>With respect to the immune function of the human body, basic research has been conducted on not only pathogens that enter the body from the outside, but also malignant tumors that arise from inside the body, and the findings of some of the studies are applied clinically. Based on the fact that many studies have shown that immune cells attack tumors, there have been a number of studies on prophylactic treatment using agents that specifically or nonspecifically enhance immunity with the involvement of lymphocytes and phagocytes. In addition, one of the most active fields of research today is to proliferate specific immune cells by cytokines in peripheral blood and to apply them to tumor immunity.<br>The cellular immunotherapy agent used in this study is the autologous killer cell (AKC), a type of autologous activated lymphocyte obtained by isolating lymphocytes from the peripheral blood and inducing selective proliferation of the lymphocytes, NK, NKT and T cells. Of these, the natural killer (NK) cells are the immune cells playing the primary function of AKC. AKC was cultured after adding a specific antigen and IL-2 to induce proliferation of the immune cells.<br>Although the safety of domestic and foreign autologous immune cell therapy agents has been reported by a number of clinical trials, the safety of AKC specifically produced by CHA Biotech's GMP facility has yet to be directly established and thus, securing safety is most important in this clinical trial. In addition, tumor is typically treated by

	surgery, radiotherapy, or chemotherapy, but most malignant tumors are difficult to treat with the above therapies alone due to their anatomical location, which makes it essential to perform adjuvant therapy to improve the prognosis. Among the available methods for adjuvant therapy, adoptive immunotherapy by autologous activated immune cells is very effective. This method of therapy is easy to administer and associated with fewer adverse effects than conventional chemotherapy and thus is expected to help improve the prognosis of cancer.
Clinical trial period	48 months from the date of approval by the Ministry of Food and Drug Safety (MFDS) and the IRB (the duration of the clinical trial or the registration period may vary)
Investigational drug	The investigational drug in this study is the AKC, a self-activating lymphocyte for cellular immunotherapy extracted from the patient's own blood
Research method (administration method, dose, duration of administration, and concomitant therapy)	1. There are two ways to collect blood for the administration of the investigational drug. The first method involves collecting 60ml of blood two weeks before the administration of the investigational drug. Then, following a culturing process for *in vitro* proliferation for 2 weeks, 100mL of the AKC (2~6 x 10^9 cells) for cellular immunotherapy, which is the finished drug product, is administered by intravenous (IV) injection for 30 min. to an hour. The second method involves collecting up to 400ml (for 6 injections) of the patient's blood in an EDTA bag for blood donation and isolating PBMC. The PBMC is cultured in a fresh or freeze-dried state, and the prescribed dose (2~6 x 10^9 cells) is administered by IV injection. The investigator may choose one of these two blood collection methods depending on the patient's condition or use a combination of these two methods, as necessary. It is possible to administer the drug product once every two weeks for a maximum of 24 doses. An anticancer drug may be administered in combination, according to the decision of the investigator, but the anticancer drug shall not be administered on the day of AKC injection. Each subject shall receive no more than 24 injections of the immunotherapy agent injections.<br>2. Safety shall be assessed in accordance with the Charter of the Data and Safety Monitoring Board (DSMB) (Attachment 7).<br>3. Tumor assessment shall be performed once every three months using MRI.
Target disease/population	Patients with recurrent glioblastoma
Number of subjects	23
Selection criteria	1) Individuals aged 20 years or older but under 70<br>2) Individuals for whom the standard therapy failed after being diagnosed with recurrent glioblastoma based on an MRI scan<br>3) Individuals whose survival period is at least 3 months<br>4) Individuals with an appropriate bone marrow function<br>- Hemoglobin count > 10 g/dL<br>- Leukocyte count > 3,000/mm^3<br>- Absolute neutrophil count > 1,500/ML<br>- Platelet count > 75.000/mm3<br>5) Individuals with appropriate liver and kidney functions<br>- Total bilirubin < 1.5 times the normal upper limit specified by the testing agency<br>- AST, ALT < 2.5 times the normal upper limit specified by the testing agency<br>- Alkaline phosphatase < 1.5 times the normal upper limit specified by the testing agency<br>- Normal kidney function (serum creatinine < normal upper limit specified by the testing agency)<br>6) Individuals who have obtained a sufficient explanation of the purpose and details of the clinical trial and the characteristics of the investigational drug from an investigator and who signed the consent form or had a legal guardian or representative sign the consent form prior to the beginning of the clinical trial<br>7) In the case of women, they must have been tested negative in a urine or blood test for pregnancy seven days before the beginning of the clinical trial and agreed to use an appropriate method of contraception during the clinical trial<br>8) Individuals who have not had any blood infection within the last 6 months<br>9) Individuals who have not received any other forms of immunotherapy

Exclusion criteria	1) An individual who is deemed to have a severe cardiopulmonary dysfunction by an investigator<br>2) An individual with immune deficiency or an autoimmune disease that may be exacerbated by immunotherapy (e.g. rheumatoid arthritis, systemic lupus erythematosus, vasculitis, multiple sclerosis, juvenile-onset insulin-dependent diabetes mellitus, etc.)<br>3) A carrier of chronic hepatitis B and/or C or who is HIV antibody positive<br>4) An individual with history of severe allergic reactions<br>5) An individual with uncontrolled hypertension or diabetes<br>6) A pregnant or lactating woman<br>7) An individual who is considered to be unfit for the clinical trial due to a serious medical or psychiatric condition<br>8) An individual with a major hemorrhagic disease not associated with cancer<br>9) An individual who has participated in another clinical trial within 4 weeks before the start of this clinical trial
Matters for observation, clinical examination items and observation method	1. Vital signs and physical examination<br>The vital signs and physical examination shall be performed at the screening visit, at every administration of the investigational drug, and at every F/U visit.<br>(1) Vital signs: Blood pressure, pulse, respiration, and body temperature<br>(2) Physical examination: Cardiovascular, respiratory, gastro-intestinal, central & peripheral nervous system, genitourinary, ENT, skin, etc.<br>1) Radiographic examination (performed only at screening)<br>- General X ray - chest PA, abdomen erect, KUB<br>- PRN - Chest CT with contrast enhancement, PET/CT, bone scan, CT, sonogram, etc. at the discretion of the investigator<br>2) Blood test<br>- Hematological tests: Hb, Hct, WBC, RBC, PLT, ESR, differential count<br>- Hematobiochemical tests: Glucose, AST, ALT, ALP, albumin, total bilirubin (BUN), creatinine, Na, K, Cl, Ca, total cholesterol, triglyceride, HDL, LDL, uric acid<br>3) Urine test: Color, specific gravity, pH, glucose, protein, β-hCG<br>4) Blood coagulation test: PT/aPTT<br>5) Autoimmune reaction test (at screening, Visit 13, Visit 27, Visit 37):<br>RF (rheumatoid factor), ANA (antinuclear antibody), CRP<br>6) Virus test (only at screening): HBs Ag, HBs Ab, anti-HCV, anti-HIV<br>(3) Electrocardiogram (ECG)<br>ECG is performed to evaluate the patient's cardiac function during screening. If an abnormality that has been found is not clinically significant, the patient shall be deemed to meet the selection criteria. If necessary, an additional test/examination shall be performed to assess cardiac function.<br>(4) Pregnancy test<br>During screening, women of childbearing age shall be subject to a blood or urine test (P-hCG) to check for pregnancy.<br>(5) Routine urinalysis<br>(6) Check whether the patient is taking any medications<br>The medications that each patient is currently taking shall be checked during the administration period of the investigational drug and on each day of administration of the investigational drug, and the details shall be recorded in the case records. Obtain details of the dose, reason for intake, start date of treatment, etc. in relation to the medications the patient is taking and make records thereof in the case records.<br>2. Assessment of the chemotherapy response<br>The assessment shall be made using MRI based on TNF-a, IL-1, and IFN-y in the patient's blood.
Withdrawal (drop-out) and termination criteria	1) A subject who is disqualified based on the selection/exclusion criteria<br>2) A serious adverse event has occurred to the subject or the subject has requested to discontinue due to an adverse event<br>3) A subject who is found to have a systemic disease that was not discovered in the

	screening test<br>4) A subject (or his/her guardian) who withdraws his/her consent to participate in the clinical trial<br>5) A serious violation of the protocol is committed by the principal investigator, a sub-investigator, or the subject in question<br>6) Recovery is not made following an adverse event<br>7) A subject who cannot be tracked<br>8) There is a problem in administering the investigational drug to the subject in question<br>9) A subject who has taken a drug, etc. that may affect the results of the study without any directions from the doctor in charge during the administration period or follow-up observation period<br>10) It is deemed by the principal investigator or sub-investigator that the study is not proceeding in an appropriate manner<br>11) The administration of the investigational drug has been postponed or cancelled three consecutive times or more during the clinical trial period or postponed or cancelled four times or more in total
Safety and effectiveness assessment criteria	The anticancer therapy response is assessed by CT or MRI, while the immune reaction is assessed based on TNF-α, IL-1, and IFN-y expression in patient's blood.<br>1. Safety assessment<br>Adverse reactions: Fever, subjective and/or objective symptoms, causal relationship with the investigational drug, etc.<br>Clinical laboratory tests<br>Vital signs and physical examination<br>2. Effectiveness assessment<br>Progression-free survival<br>Overall survival<br>Treatment response rate according to the tumor evaluation<br>QLQ-C30 (Ver 3.0) Korean<br>Karnofsky performance status (KPS)<br>Evaluation of immune reaction
Safety and effectiveness assessment and analysis methods (methods of statistical analysis)	General principles<br>The data obtained from the subjects through this clinical trial are largely divided into three groups: Safety, Full Analysis (FA) and Per-Protocol (PP). The main population of analysis in this clinical trial is the FA group, while additional analysis is performed on the PP group and safety is analyzed based on the Safety group. However, in the case of the FA group for the primary effectiveness assessment, the analysis group shall be redefined by setting forth the exclusion criteria for the subjects taking into consideration of the characteristics of the assessment variables. The results of all three groups shall be compared, and when the results differ among the groups, the results of each analysis method shall be presented with the reasons for the differences described in detail. All of the analyses shall be performed at a significance level of 5%.<br>Safety assessment method<br>1) Adverse events: The percentage of subjects who experienced an adverse event and the 95% confidence interval in relation thereto shall be presented. All of the adverse events that have occurred shall be documented according to the level of severity. The adverse events and serious adverse events associated with the investigational drug shall also be documented.<br>2) Clinical laboratory tests: In the case of continuous data, the descriptive statistics (mean, standard deviation, minimum value, maximum value, etc.) shall be presented according to the time of visit, while in the case of categorical data, the frequency and ratio for each category shall be presented. For continuous variables, a paired t-test or Wilcoxon signed-rank test shall be performed on the differences between before and after the administration of the investigational drug to examine the differences between the visits. McNemar's test shall be carried out for categorical variables. In addition, clinically significant non-normalized rates compared to before treatment shall be presented.

	3) Vital signs and physical examination: The descriptive statistics for vital signs (blood pressure, pulse, and body temperature) shall be presented, and a shift table indicating the changes between before and after the administration of the investigational drug with respect to the normal and abnormal values measured in the physical examination shall be presented. The before and after differences shall be analyzed by a paired t-test or Wilcoxon signed-rank test for continuous variables and the McNemar's test for categorical variables.<br><br><u>Effectiveness assessment method</u><br>1) Overall survival (OS): OS shall be the period from the date on which the individual was registered as a subject of this clinical trial and assigned a number to the date on which he/she died, irrespective of the cause. OS shall be estimated by the Kaplan-Meier method, and the median survival time shall be calculated.<br>2) Progression-free survival (PFS): PFS shall be the period from the date on which the individual was registered as a subject of this clinical trial and assigned a number to the date on which a lesion arose or on which he/she died. The progress of the glioblastoma shall be assessed before and after the administration of the investigational drug at Week 12, Week 24, Week 36, Week 48, Week 60, and Week 68 based on a CT or MRI scan. The PFS of patients without any progress or recurrence of tumor at final analysis shall be determined as the period from the date on which he/she was registered as a subject of this clinical trial and assigned a number to the date of the final tumor assessment. PFS, without any progression of the disease, shall be estimated by the Kaplan-Meier method, and the median survival time shall be calculated.<br>3) Response rate: A tumor response assessment shall be carried out, in accordance with mRECIST (Appendix 3), before and after the administration of the investigational drug at Week 12, Week 24, Week 36, Week 48, Week 60, and Week 68 based on a CT or MRI scan. The disease control rate (DCR), which is defined as the ratio of the subjects displaying an overall response that is classified as CD, PR or SD to the total number of subjects, shall be presented, with the 95% confidence interval indicated.<br>4) QLQ-C30 (Ver 3.0) Korean: The measurements for each item shall be compared in order to determine the level, and the changes according to the progress of treatment shall be analyzed. The descriptive statistics (mean, standard deviation, median, minimum value, and maximum value) shall be presented with respect to the QLQ-C30 (Ver 3.0) Korean evaluation scores and depending on the presence of normality, a paired t-test or Wilcoxon's signed-rank test shall be performed for a comparison between before and after the administration of the investigational drug.<br>5) Karnofsky performance status (KPS): The measurements for each item shall be compared in order to determine the level, and the changes according to the progress of treatment shall be analyzed. The descriptive statistics (mean, standard deviation, median, minimum value, and maximum value) shall be presented with respect to the KPS evaluation scores and depending on the presence of normality, a paired t-test or Wilcoxon's signed-rank test shall be performed for a comparison between before and after the administration of the investigational drug.<br>6) Evaluation of immune reaction<br>Statistical analysis of the immune reaction evaluation: The analysis can be carried out by a repeated measure of ANOVA in relation to the change from baseline or measured value, depending on the nature of the variable, or the Chi square text(X^2) or Fisher's exact test may be applied to compare the frequency of certain levels between the groups or immune reactions.
Measures for ensuring safety of subjects	In case any of the subjects in this clinical trial suffers from an adverse event with a causal relationship with the investigational drug used in this clinical trial, notwithstanding the fact that the investigators have complied with the relevant laws and regulations and performed the clinical trial in strict adherence to the protocol, the principal investigator shall follow the Victim Compensation Regulations submitted to the IRB and provide reasonable compensation for treatment within the scope of compensations that are not provided by health insurance, the government or a third party.

Research-based clinical literature (references)	1. N Engl J Med (2005)352(10);987-96<br>2. J Neuro-Oncology (2005)72;57-62<br>3. J Clin Oncol (2005)23(10);2372-7<br>4. Rosenberg SA. Progress in human tumour immunology and immunotherapy. Nature 2001; 411:380-4.

2. Schedule

(Whether to administer the investigational drug and an anticancer agent to a subject shall be determined based on the CBC results. If the subject is shown to have WBC count of 4,000 or lower based on a blood test, the anticancer therapy and AKC administration schedule may be skipped at the discretion of the principal investigator (PI) due to concerns of a drop in the WBC count or that the subject may not be in a suitable condition for anticancer therapy.)
In the following cases, the anticancer therapy and AKC administration schedule may be skipped at the discretion of the PI: 1) If the WBC count is 4,000 or lower
If the administration of the investigational drug is postponed or cancel three consecutive times or more or four times in total, the subject in question shall be removed from the clinical trial, in accordance with the withdrawal (drop-out) and termination criteria.

	Screening		Treatment period[1]																		Termination[2]	Survival[3]
Visit	1	2	3	4	5	6	7	8	9	10	11	12	13	14	15	16	17	18	19	20		
Week	Within -3	Within -2	0	2	4	6	8	10	12	14	16	18	20	22	24	26	28	30	32	34		Every 4 weeks
Written consent	✓																					
Assignment of screening number	✓																					
Demographic survey[4]	✓																					
Investigation of medical history[5]	✓																					
Investigation of medication history[6]	✓		✓	✓	✓	✓	✓	✓	✓	✓	✓	✓	✓	✓	✓	✓	✓	✓	✓	✓	✓	
Vital signs[7]	✓		✓	✓	✓	✓	✓	✓	✓	✓	✓	✓	✓	✓	✓	✓	✓	✓	✓	✓	✓	
ECG	✓																					
Physical examination[8]	✓		✓	✓	✓	✓	✓	✓	✓	✓	✓	✓	✓	✓	✓	✓	✓	✓	✓	✓	✓	
Pregnancy test[9]	✓																					
Clinical laboratory tests[10]	✓		✓		✓		✓		✓		✓		✓		✓		✓		✓		✓	
QLQ-C30 (Ver 3.0)	✓		✓		✓		✓		✓		✓		✓		✓		✓		✓		✓	
KPS	✓		✓		✓		✓		✓		✓		✓		✓		✓		✓		✓	
Selection/exclusion criteria		✓																				
Assignment of registration number		✓																				
Blood collection[11]		✓	✓	✓		✓	✓		✓	✓		✓	✓		✓	✓		✓	✓			
Administration of investigational drug[12]			✓	✓	✓		✓	✓		✓	✓		✓	✓		✓	✓		✓	✓		
Administration of anticancer drug[13]	✓	✓				✓			✓			✓			✓			✓			✓	
Tumor evaluation[14]	✓								✓						✓						✓	
Immune reaction evaluation[15]		✓																			✓	

Check for adverse events			✓	✓	✓	✓	✓	✓	✓	✓	✓	✓	✓	✓	✓	✓	✓	✓	✓	✓	✓	✓
Radiography	✓																					

	Treatment period[1)]																	Additional visit[16)]	Termination[2)]	Survival[3)]
Visit	21	22	23	24	25	26	27	28	29	30	31	32	33	34	35	36	37	Unscheduled Visit		
Week	36	38	40	42	44	46	48	50	52	54	56	58	60	62	64	66	68			Every 4 weeks
Written consent																				
Assignment of screening number																				
Demographic survey[4)]																				
Investigation of medical history[5)]																				
Investigation of medication history[6)]	✓	✓	✓	✓	✓	✓	✓	✓	✓	✓	✓	✓	✓	✓	✓	✓	✓	✓	✓	
Vital signs[7)]	✓	✓	✓	✓	✓	✓	✓	✓	✓	✓	✓	✓	✓	✓	✓	✓	✓	✓	✓	
ECG																				
Physical examination[8)]	✓	✓	✓	✓	✓	✓	✓	✓	✓	✓	✓	✓	✓	✓	✓	✓	✓	✓	✓	
Pregnancy test[9)]																				
Clinical laboratory tests[10)]	✓		✓		✓		✓		✓		✓		✓		✓		✓	✓	✓	
QLQ-C30 (Ver 3.0)	✓		✓		✓		✓		✓		✓		✓		✓		✓	✓	✓	
KPS	✓		✓		✓		✓		✓		✓		✓		✓		✓			
Selection/exclusion criteria																				
Assignment of registration number																				
Blood collection[11)]	✓	✓		✓	✓		✓	✓		✓	✓		✓	✓	✓					
Administration of investigational drug[12)]		✓	✓		✓	✓		✓	✓		✓	✓		✓	✓	✓				
Administration of anticancer drug[13)]	✓			✓			✓			✓			✓				✓	✓	✓	
Tumor evaluation[14)]	✓						✓						✓				✓	✓	✓	
Immune reaction evaluation[15)]	✓																✓	✓	✓	
Check for adverse events	✓	✓	✓	✓	✓	✓	✓	✓	✓	✓	✓	✓	✓	✓	✓	✓	✓	✓	✓	✓
Radiography																				

1) Treatment period:
All visits shall be made within ± 3 days of the scheduled date. However, in the case of Visit 2 and Visit 3, it shall be made within 2 to 8 weeks of the scheduled date. Visits may be delayed for up to two weeks in the event of a problem with the supply of the investigational drug.

2) Termination:
A final visit for termination or discontinuation of the clinical trial shall be scheduled in the event that any of the withdrawal (drop-out) and termination criteria applies.
3) Survival:
After the clinical trial is terminated for a reason other than withdrawal of consent, the survival information on the subjects shall be collected once every 4 weeks in person or by phone after the termination visit.
4) Demographic Survey:
The subjects' initials, sex, age, height and weight shall be determined, and the body surface area (BSA) shall be calculated for the administration of the anticancer drug.
5) Investigation of medical history:
The onset of prior medical conditions within the past 5 years and current medical conditions shall be examined.
6) Investigation of medication history:
The subjects' anticancer therapy history shall be investigated through an inquiry (verbal or questionnaire) during the screening process, and as for the use of other types of pharmaceutical drugs, the history of taking drugs in the past 3 months and the current administration status (dosage and directions, duration of administration, etc.) shall be investigated. After registration, the subjects' medical history shall be examined at every visit and all of the drugs taken before the visit (administration of anticancer agent) shall be documented.
7) Vital signs:
The body temperature, pulse and blood pressure shall be checked.
8) Physical examination:
The subjects' external appearance, skin, head and neck, chest and lungs, heart, abdomen, urinary and reproductive systems, limbs, musculoskeletal system, nervous system, lymph nodes and other body organs shall be examined.
9) Pregnancy test:
For women of childbearing age, an HCG response test shall be performed using a urine sample.
10) Clinical laboratory tests

-Hematological test:	Hb, Hct, WBC, RBC, PLT, ESR, differential count
-Virus test:	HBs Ag, HBs Ab, anti-HCV, anti-HIV (only at screening)
-Hematobiochemical test:	Glucose, AST, ALT, ALP, albumin, total bilirubin , BUN, creatinine, Na, K, Cl, Ca, total cholesterol, triglyceride, HDL, LDL, Uric acid
- Blood coagulation test:	PT/aPTT (only at screening)
- Autoimmune reaction test:	RF (Rheumatoid Factor), ANA (Antinuclear Antibody), CRP (only at screening, Visit 13, Visit 27, Visit 37)
-Urine test:	Color, pH, specific gravity, protein, glucose

If there are test results obtained within four weeks prior to the date on which the written consent was obtained, the said results may be used instead.
11) Blood collection
60ml or a maximum of 400ml (determined by the PI based on the subject's condition) of blood shall be collected to produce the cellular therapy agent (investigational drug).
The blood collection schedule may be changed depending on the anticancer drug administration schedule. If the subject weighs less than 50kg, the amount of blood that can be collected shall be limited to 320ml maximum per collection session. In this case, additional blood of 400ml in total may be collected depending on the administration frequency and schedule.
12) Administration of investigational drug
The investigational drug shall be administered once every two weeks for a maximum of 24 doses. The anticancer drug shall not be administered on the day of

AKC injection
An anticancer drug may be administered in combination at the discretion of the investigator.
Whether to administer the investigational drug and an anticancer agent to a subject shall be determined based on the CBC results. If the subject is shown to have WBC count of 4,000 or lower based on a blood test, the anticancer therapy and AKC administration schedule may be skipped at the discretion of the principal investigator (PI) due to concerns of a drop in the WBC count or that the subject may not be in a suitable condition for anticancer therapy
13) Administration of anticancer drug
The administration of an anticancer drug shall be started depending on the method of administration of the anticancer drug. Depending on the subject, use BCNU (bis-chloroethylnitrosourea), ACNU (Nimustine) PCV therapy, or Nolvadex Tab. However, the administration of the anticancer drug may be rescheduled or canceled at the discretion of the investigator, depending on the subject's condition.
14) Tumor evaluation
15) Immune reaction evaluation for evaluating the response to the anticancer therapy
The immune reaction is determined based on an MRI scan, and the measurement method shall be based on RECIST. The MRI scan shall be performed at 3-month intervals. The tumor evaluation must be carried out within 7 days.
Measurements shall be taken at Visit 2 (within -2 weeks), Visit 21 (36 weeks), and Visit 37 (final visit; 68 weeks) for a total of three times. However, the immune reaction evaluation shall be performed before the administration of the anticancer agent or the cellular therapy agent. Also, if the cellular therapy agent is injected without the administration of an anticancer agent, the second exam shall be performed during a visit after the 13th dose. – Immune reaction evaluation: TNF-α, IL-1, IFN-γ
16) Additional visit:
During an additional visit, only the necessary tests/examinations shall be performed.

3. Background of the clinical trial

Glioma accounts for approx. 35% of all primary brain tumors, while glioblastoma accounts for around 40% of all glioma cases. Glioblastoma, which occurs commonly in men aged between 45 and 65, is the most aggressive malignant glioma and is very difficult to treat. Although glioblastomas form a mass in some cases, most exhibit invasive growth into the surrounding tissue, rendering treatment by a localized surgical procedure impossible.

With the introduction of nitrosoureas in the early 1970s, numerous clinical trials have been carried out with patients suffering from primary or recurrent malignant brain tumors in order to examine the therapeutic effects of anticancer agents. According to a meta-analysis, chemotherapy is recommended in combination with radiation therapy following surgery, but the 5-year survival rate is very low at under 2%. Thus, the purpose of treatment for glioblastoma, which has a grim prognosis as such, is to extend the survival period as much as possible and to manage the disease so that the patient's quality of life does not deteriorate while he/she is still alive.

Weller M. et al. (Neuro Oncol. 2012 Nov 7. [Epub ahead of print]) reviewed the abstracts on PubMed and American Society of Clinical Oncology from Jan. 2006 until Jan. 2012, and examined the clinical trials that used anticancer drugs such as nitrosoureas, which are the most common type of drug prescribed for recurrent glioblastoma patients, as well as temozolomide and bevacizumab, which have recently been developed and are now used regularly.

It has been reported that reoperation or reirradiation is attempted for some patients and there is increased toxicity from the anticancer drugs used afterwards, but the progression-free survival rate at 6 months is only 20~30%. The aim of this study is, therefore, to try a new type of immunotherapy in such cases where further treatment cannot be anticipated in the event of recurrence.

With respect to the immune function of the human body, basic research has been conducted on not only pathogens that enter the body from the outside, but also malignant tumors that arise from inside the body, and the findings of some of the studies are applied clinically. Based on the fact that many studies have shown that immune cells attack tumors, there have been a number of studies on prophylactic treatment using agents that specifically or nonspecifically enhance immunity with the involvement of lymphocytes and phagocytes. In addition, one of the most active fields of research today is to proliferate specific immune cells by cytokines in peripheral blood and to apply them to tumor immunity.

According to the study published in Anticancer Research 24: 1861-1872 (2004) by Ishikawa et al., the autologous natural killer (NK) cells are safe to use with partial effectiveness. As shown in the following table, autologous NK cells have no neurological toxicity and induce a partial response.

Table IV. *Responses and neurological toxicities of NK therapy.*

Case No.	Course of therapy	Total (x10^9)	dose (-fold)	Injected route	(*i.c.* %)	Response to the therapy	Neuro-logical toxicity	Posttreatment K.P.S. (from initial NK cell injection to outcome)
1	1	3.1	43.7	*i.v.*	(0)	NC	Grade 0	Dead(18 months)
2	1	3.3	43.6	*i.v.+i.c.*	(50)	PD	Grade 0	Dead (6 months)
3	1	0.6	11.3 #	*i.v.*	(0)	PR	Grade 0	50%
	2	3.5	52.5	*i.v.*	(0)	PD	Grade 0	40%
	3	6.5	90.1	*i.v.*	(0)	PD	Grade 0	Dead(11 months)
4	1	0.7	12.1 #	*i.v. +i.c.*	(51)	NC	Grade 0	10%
	2	2.5	42.6	*i.v.*	(0)	MR	Grade 0	10% (15 months)
5	1	5.1	52.7	*i.v.+i.c.*	(43)	PR	Grade I*	70%
	2	4.6	64.1	*i.v.+i.c.*	(50)	PR	Grade I*	70%
	3	4.0	54.9	*i.v.+i.c.*	(50)	PD	Grade 0	70% (12 months)
6	1	0.6	13.9	*i.v.+i.c.*	(65)	PD	Grade 0	Dead**
7	1	4.3	59.6	*i.v.+i.c.*	(13)	NC	Grade 0	Dead (4 months)
8	1	1.8	63.6	*i.v.+i.c.*	(50)	MR	Grade 0	40%
	2	1.7	23.8 #	*i.v.+i.c.*	(50)	PD	Grade 0	40% (5 months)
9	1	0.8	11.5	*i.v.*	(0)	NC	Grade 0	80%
	2	3.2	44.7	*i.v.+i.c.*	(50)	PD	Grade 0	80% (5 months)

# Data regarding cell expansion that proceeded with difficulty are included in the calculation.
* Headache, nausea and irritability.
** Another therapy was performed one month after the period of NK therapy.
i.v.: intravenous; *i.c.*: intracranial.

The cellular immunotherapy agent used in this study is the autologous killer cell (AKC), a type of autologous activated lymphocyte obtained by isolating lymphocytes from the peripheral blood and inducing selective proliferation of the lymphocytes, NK, NKT and T cells. Of these, the natural killer (NK) cells are the immune cells playing the primary function of AKC.

AKC is a cellular immunotherapy agent manufactured at CHA Biotech's GMP facility and it will be applied to the human body for the first time as a medicinal product. AKC used in this study was cultured for around two weeks after adding a specific antigen and IL-2 to induce proliferation of the immune cells.

Although the safety of domestic and foreign autologous immune cell therapy agents has been reported by a number of clinical trials, the safety of AKC specifically produced by CHA Biotech's GMP facility has yet to be directly established and thus, securing safety is most important in this clinical trial. In addition, tumor is typically treated by surgery, radiotherapy, or chemotherapy, but most malignant tumors are difficult to treat with the above therapies alone due to their anatomical location, which makes it essential to perform adjuvant therapy to improve the prognosis. Among the available methods for adjuvant therapy, adaptive immunotherapy by autologous activated immune cells is very effective. This method of therapy is easy to administer and associated with fewer adverse effects than conventional chemotherapy and thus is expected to help improve the prognosis of cancer.

4. Purpose of the clinical trial

The purpose of this clinical trial is to administer the autologous killer cell, the investigational drug, to patients with recurrent glioblastoma and monitor the safety and effectiveness of the immunotherapy.

5. Clinical trial period

48 months from the date of approval by the Ministry of Food and Drug Safety (MFDS) and the IRB (the duration of the clinical trial or the registration period may vary)

6. Medicinal products used in the clinical trial

6.1 Investigational drug

1) Name of the formulation: AKC

2) Properties and dosage form: Autologous activated lymphocytes in light yellow color filled inside an opaque polyethylene container
3) Amount of active ingredient
Main ingredient: Autologous activated lymphocytes (2~6 x 10^9 cells)

6.2 Investigational drug management and records

The investigational drug in this clinical trial must be manufactured individually for each subject at CHA Biotech's GMP facility to be supplied to the sub-investigator and be administered to the subjects within 24 hours after manufacture.
1) After manufacturing the investigational drug, the sub-investigator shall be contacted to inform them of the delivery schedule and the investigators shall check the administration schedule in order to administer the investigational drug.
2) CHA Biotech's GMP facility shall deliver the investigational drug to the sub-investigator, who shall then check the investigational drug and sign and record the date on the hand-over sheet.
3) The investigator shall check the investigational drug and administer it to the subjects.
4) After completing the administration process, dispose of the transfer bag according to the hospital regulations.

6.3 Production, packaging and labeling of the investigational drug

A single dose of the investigational drug shall be packaged individually in a transfer bag. It shall be labeled and delivered to the clinical trial institution with the related records.
The sub-investigator designated by the principal investigator shall be responsible for the acquisition, storage, management and return of the investigational drug. The packaging container for the investigational drug shall be have a label with the following information in Korean, and the investigational drug shall be supplied to the clinical trial institution after the packaging process. The sub-investigator designated by the principal investigator shall handle and store the delivered investigational drug in a safe and appropriate manner. The investigational drug shall be stored in a safe location that is accessible only to the investigators and designated personnel.

• Labeled as "Investigational Drug"
• The product's code name or the general name of the active ingredient
• Lot number and shelf life or re-examination date
• Storage method
• Name and address of the party that acquired approval for the clinical trial protocol
• Labeled "Cannot be used for purposes other than the clinical trial"
• Registration number

6.4 Investigational drug manager

Name	Affiliation	Contact number
CHO, Kyung Gi	CHA Bundang Medical Center Dept. of Neurosurgery	031-780-5263

The investigational drug manager shall be responsible for the acquisition, storage, management and return of the drugs used in the clinical trial at the clinical trial institution.

7. Subject selection criteria, exclusion criteria, target number of subjects, and calculation basis

7.1 Selection criteria

1) Individuals aged 20 years or older but under 70
2) Individuals for whom the standard therapy failed after being diagnosed with recurrent glioblastoma based on an MRI scan
3) Individuals whose survival period is at least 3 months
4) Individuals with an appropriate bone marrow function

- Hemoglobin count > 10 g/dL
- Leukocyte count > 3,000/mm^3
- Absolute neutrophil count > 11,500/μL
- Platelet count > 75.000/mm^3

5) Individuals with appropriate liver and kidney functions
- Total bilirubin ≤ 1.5 times the normal upper limit specified by the testing agency
- AST, ALT ≤ 2.5 times the normal upper limit specified by the testing agency
- Alkaline phosphatase ≤ 1.5 times the normal upper limit specified by the testing agency
- Normal kidney function (serum creatinine ≤ normal upper limit specified by the testing agency)

6) Individuals who have obtained a sufficient explanation of the purpose and details of the clinical trial and the characteristics of the investigational drug from an investigator and who signed the consent form or had a legal guardian or representative sign the consent form prior to the beginning of the clinical trial
7) In the case of women, they must have been tested negative in a urine or blood test for pregnancy seven days before the beginning of the clinical trial and agreed to use an appropriate method of contraception during the clinical trial
8) Individuals who have not had any blood infection within the last 6 months
9) Individuals who have not received any other forms of immunotherapy

7.2 Exclusion criteria

1) An individual who is deemed to have a severe cardiopulmonary dysfunction by an investigator
2) An individual with immune deficiency or an autoimmune disease that may be exacerbated by immunotherapy (e.g. rheumatoid arthritis, systemic lupus erythematosus, vasculitis, multiple sclerosis, juvenile-onset insulin-dependent diabetes mellitus, etc.)
3) A carrier of chronic hepatitis B and/or C or who is HIV antibody positive
4) An individual with history of severe allergic reactions
5) An individual with uncontrolled hypertension or diabetes
6) A pregnant or lactating woman
7) An individual who is considered to be unfit for the clinical trial due to a serious medical or psychiatric condition
8) An individual with a major hemorrhagic disease not associated with cancer
9) An individual who has participated in another clinical trial within 4 weeks before the start of this clinical trial

7.3 Target number of subjects

The clinical trial shall be carried out on a total of 23 subjects with recurrent glioblastoma among the applicants who meet the selection criteria and do not fall under any of the exclusion criteria.

7.4 Basis for calculating the target number of subjects

As this clinical trial is an exploratory study, the number of subjects was not calculated based on statistical significance.

7.5 Random assignment

This is a single-arm trial, so randomization is not applicable.

7.6 Clinical trial design

This is a prospective, open, single-arm, investigator-initiated trial carried out for the purpose of observing the safety and effectiveness of AKC, a cellular immunotherapy agent, in treating patients with recurrent glioblastoma.
A relatively safe dose of 2-6 x 10^9 cells was set as a single dose based on the clinical data reported in the existing literature. In addition, it is safer and more efficient to administer a safe dose reported in literature and carefully review the response before conducting additional research, rather than carrying out a toxicity test using a dose that has already been verified, in case significant overall survival (OS) or progression-free survival (PFS) cannot be expected from a standard therapy for patients with recurrent glioblastoma. Thus, a single dose of 2-6 x 10^9 cells shall be administered to the 23 subjects and the safety and effectiveness thereof shall be assessed thereafter. The safety assessment shall be

performed by the DSMB.

7.7 Basis for setting the dose

Ishikawa et al. carried out 16 courses of cellular immunotherapy to patients with malignant glioma, with a dose of 0.6 x 10^9~6.5 x 10^9 cells. One course consisted three injections in a week, with 1 x 10^9 cells administered per injection, and the assessment was performed four to five weeks after treatment. The clinical results showed little to no neurological toxicity or adverse events after 9 courses and excellent cancer-killing ability. Clinical effectiveness was achieved by administering large amounts of a high-purity cellular immunotherapy agent.

Sally et al., on the other hand, administered 1 x 10^8 ~ 3 x 10^9 cells by increasing the dose incrementally to patients with kidney cancer or melanoma, and assessed the safety over the course of a 4-week treatment period after one course (3 injections). The group administered 1 x 10^8, 3 x 10^8 cells experienced no adverse events, while only one out of the three subjects administered 1 x 10^9 cells experienced a Grade 1 fever, whereas the other two subjects exhibited a stable disease condition. Meanwhile, three subjects administered 3 x 10^9 cells reported adverse events ranging from Grade 1 to 4, but they all exhibited either a stable disease condition or minor response, without death.

The objective of this study is to assess the therapeutic safety and effectiveness of the cellular immunotherapy agent in question in patients with recurrent glioblastoma, and 2~6 x 10^9 cells were set as a single dose, as it was deemed to be a safe dose in reference to the aforementioned clinical trials.

7.8 Method and duration of administration

There are two ways to collect blood for the administration of the investigational drug. The first method involves collecting 60ml of blood two weeks before the administration of the investigational drug. Then, following a culturing process for *in vitro* proliferation for 2 weeks, 100mL of the AKC (2~6 x 10^9 cells) for cellular immunotherapy, which is the finished drug product, is administered by intravenous (IV) injection for 30 min. to an hour. The second method involves collecting up to 400ml (for 6 injections) of the patient's blood in an EDTA bag for blood donation and isolating PBMC. The PBMC is cultured in a fresh or freeze-dried state, and the prescribed dose (2~6 x 10^9 cells) is administered by IV injection. The investigator may choose one of these two blood collection methods depending on the patient's condition or use a combination of these two methods, as necessary. It is possible to administer the drug product once every two weeks for a maximum of 24 doses. An anticancer drug may be administered in combination, according to the decision of the investigator, but the anticancer drug shall not be administered on the day of AKC injection. Tumor evaluation shall be performed using MRI every three months.

Visits may be postponed for up to two weeks in case it is difficult to supply the investigational drug due to an issue with production (e.g. culturing failure, infection, etc.) or due to the subject's condition. However, if it is deemed that postponing all other schedules including anticancer drug administration for 2 weeks will disrupt the treatment process, the visit in question shall be skipped and the investigational drug shall be administered during the next scheduled visit. If the investigational drug administration is postponed or canceled two times or more within a cycle, the subject in question shall be excluded from the PP group. If it is postponed or cancelled three consecutive times or more or four times or more in total, the subject in question shall be removed from the clinical trial will be canceled. If a subject drops out or is removed, a new subject may be registered in his/her place. In addition, if the sterility test carried out as part of GMP quality control for AKC yield a positive result, the following steps shall be taken:

- If the microbial test result of the AKC administered to a subject is positive:

① Request the GMP facility to identify the microorganism.

② Collect the subject's blood and perform a microbial culture test.

③ Administer an appropriate antibiotic treatment according to the identified microorganism.

④ Perform a microbial culture test again after the antibiotic treatment and confirm the absence of microorganisms.

7.9 Conservative treatment

If blood transfusion is necessary (e.g. platelet transfusion to keep the platelet count above 20,000/μL), it may be carried out based on the judgment of the investigator. In case of deep vein thrombosis or

pulmonary embolism, heparin, warfarin, etc. may be used for treatment. Other conservative treatments shall be carried out at the discretion of the investigator and be documented in detail in the case records.

7.10 Drugs that can be used in combination

In this clinical trial, all kinds of drugs may be administered in combination. However, in the case of anticancer agents that can be administered, they shall be restricted to BCNU, ACNU, PCV therapy, and Nolvadex tab.

7.11 Drugs that cannot be used in combination

There are no drugs prohibited in this clinical trial except for cell therapy agents.
However, if it is deemed by the investigator or the doctor responsible for treating other medical conditions of the subjects that it is necessary to use drugs in combination to treat the subjects during the clinical trial period, the related information must be documented in the case records in detail.

7.12 Matters for observation and observation method

7.12.1.1 Written consent and demographic survey

Before initiating the clinical trial, explain the purpose and details of the trial to the subjects in detail and obtain their written consent. Assign a screening number to each of the subjects in the order that the consent was provided and obtain their demographic information.
Check the subjects' demographic information such as initials, sex, and age, measure their height and weight, and calculate the body surface area (BSA) when administering an anticancer drug.

7.12.1.2 Investigation of medical and medication history

Obtain detailed information on the subjects' medical and medication history through an inquiry (verbal or questionnaire) and by checking their prior medical records, and record the information. The information that must be obtained is as follows:
Document the onset (year of occurrence or year and month of occurrence) of prior medical conditions in the last 5 years and current medical conditions including surgery, allergies, etc. and write down the investigator's comments and opinions. With respect to glioblastoma, the medical history including the duration of the disease shall be examined in detail through the inquiry process.
As for the medication history examined during screening, all prior anticancer therapies must be examined through an inquiry. As for other types of drugs used, the history of taking drugs in the past 3 months and the current administration status (dosage and directions, duration of administration, etc.) shall be investigated.
After registration, the subjects' medical history shall be examined at every visit and all of the drugs taken before the visit (administration of anticancer agent) shall be documented. However, information on general infusions and infusion preparations for mixing needs not be documented.

7.12.1.3 Measurement of vital signs

The vital signs such as blood pressure, heart rate, and body temperature shall be measured at each visit before the test/examination scheduled for the subject.

7.12.1.4 Physical examination

The physical examination shall be carried out at every visit. This examination includes an examination of external appearance, skin, head and neck, chest and lungs, heart, abdomen, urinary and reproductive systems, limbs, musculoskeletal system, nervous system, lymph nodes and other body organs shall be examined.
Any significant findings discovered during the screening test shall be recorded in the section on physical examination in the case records. In case of discovering any significant matters in the physical findings that fall under the definition of "adverse event" after initiating the administration of the investigational drug, the information shall be recorded in the section on adverse events in the case records. However, if an undesirable physical symptom develops prior to initiating the administration of the investigational drug, it shall be recorded in addition to the section on current medical conditions.

7.12.1.5 Pregnancy test and clinical laboratory tests

At the screening, women of childbearing age shall be subject to an HCG response test using a urine sample to determine whether they are pregnant, except for women who have undergone a sterilization operation or who are in menopause (incl. menopause caused by surgery).

Clinical laboratory tests shall be carried out at Visit 3 during the screening process and at 1-month intervals thereafter until the final visit. On days when drug administration is scheduled, these tests shall be performed before the administration of the investigational drug. However, if there are test results obtained within four weeks prior to the date on which the written consent was obtained, the said results may be used instead.

7.12.1.6 Tumor evaluation

Tumor evaluation shall be performed once at the screening and every three months thereafter by using a brain MRI scan. Brain MRI scans are carried out to acquire axial (T1-pre & post, T2, proton), coronal (T1-post), and sagittal (T1-post) images. However, tumor evaluation results obtained within four weeks prior to the date on which the written consent was obtained, the said results may be used instead for the screening process.

The tumor evaluation criteria are as follows, and the tumor measurement method will involve the use of RECIST (Version 1.1).

"Treatment response" refers to a case in which a "complete response" or a "partial response" among the following five cases.

The terms used in the tumor evaluation and the definitions thereof are as follows:

- Complete Response (CR): Disappearance of all target lesions. Any pathological lymph nodes (whether target or non-target) must have reduction in short axis to < 10mm
- Partial Response (PR): At least a 30% decrease in the sum of diameters of target lesions, taking as reference the baseline sum diameters
- Progressive Disease (PD): At least a 20% increase in the sum of diameters of target lesions, taking as reference the smallest sum on study. In addition to the relative increase of 20%, the sum must also demonstrate an absolute increase of at least 5 mm.
- Stable Disease (SD): Neither a partial response (PR) nor progressive disease (PD)
- Not evaluated (NE): Applicable only if none of the target lesions have been evaluated or are evaluable, or only a procedure was carried out on the lesions. Note: If the sum of the diameters meets the criteria for disease progression, disease progression in the target lesion response takes precedence over NE.

As for the evaluation of the immune reactions of the subjects, other than the tumor evaluation, the expression of TNF-α, IL-1, and IFN-γ shall be examined using the subjects' blood samples.

7.12.1.7 KPS, QLQ-C30 (ver 3.0) Korean

Implement the Karnofsky performance status (KPS) examination for neurological examination and QLQ-C30 (Ver 3.0) Korean to examine the subjects' health status. These tests shall be carried out at screening and once every month until the final visit.

7.12.1.8 Screening based on selection/exclusion criteria (subject suitability assessment)

Select patients who meet the selection criteria based on the medical history and basic survey data and conduct a screening test after obtaining their written consent. Carry out a final evaluation at Visit 2 to check whether the subjects conforming to the selection criteria and exclusion criteria have been chosen, based on a comprehensive review of the screening tests. If a subject fails the screening test for any other reasons, a re-screening test may be performed once.

7.12.1.9 Investigational drug

The administration of the investigational drug shall be initiated at Visit 3 and terminated at Visit 36. The investigational drug shall be administered once every two weeks for up to 24 times and an anticancer agent may be administered in combination at the discretion of the investigator. Proceed with the next stage only if there are no problems with safety examined every three months and the tumor

evaluation result is SD or higher. The visit following the 24 injections shall be the final visit.

7.12.1.10 Administration of anticancer agent

The administration of an anticancer drug shall be started depending on the method of administration of the anticancer drug. Depending on the subject, use BCNU (bis-chloroethylnitrosourea), ACNU (Nimustine) PCV therapy, or Nolvadex Tab. However, the administration of the anticancer drug may be rescheduled or canceled at the discretion of the investigator, depending on the subject's condition.

7.12.1.11 Investigation into adverse events

Once the administration of the investigational drug is initiated, the subjects shall be instructed to voluntarily report any adverse events and the occurrence of adverse events shall be checked at every visit through an interview or inquiry (verbal or questionnaire). The adverse events shall be documented in the case records in detail in relation to the date of occurrence and disappearance, the severity and consequences of the adverse event, the causal relationship between the investigational drug and the measures taken in relation to the investigational drug, the name of the drug other than the investigational drug suspected to have caused the adverse event, the treatment of adverse events and other details. The adverse events shall be categorized according to the Common Terminology Criteria for Adverse Events (CTCAE) attached hereto, in principle. If applying these standards is not appropriate, however, another general standard classification method shall be used for the evaluation.

7.12.2 Clinical trial method by visit

The window period for every visit shall be designated as ±3 days.

7.12.2.1 Visit 1 (within -3 weeks; screening)

The subjects who have been selected to participate in this study shall receive an explanation of the clinical trial and assessed in the following order:

⑭ Acquisition of written consent
⑮ Assignment of screening number
16 Demographic survey: Initials, sex, age, height, weight, BSA
17 Investigation of medical history: Prior medical conditions within the last 5 years and current medical conditions incl. surgical operations
18 Investigation of medication history: All types of anticancer treatments and other drugs administered within the last 3 months
19 Vital signs: Blood pressure, pulse, body temperature
20 ECG
21 Physical examination: External appearance, skin, head and neck, chest and lungs, heart, abdomen, urinary and reproductive systems, limbs, musculoskeletal system, nervous system, lymph nodes and other body organs
22 Pregnancy test (female subjects of childbearing age)
23 Clinical laboratory tests: Hematological test, virus test, hematobiochemical test, blood coagulation test, autoimmune reaction test, urine test
(may be substituted by test results obtained within 4 weeks prior to the acquisition of the written consent)
24 KPS, QLQ-C30 (Ver 3.0) Korean examinations
25 Tumor evaluation (may be substituted by test results obtained within 4 weeks prior to the acquisition of the written consent)
26 Scheduling of next visit
27 Radiography
28 Administration of an anticancer agent (not on days when AKC is injected)

7.12.2.2 Visit 2 (within -2 weeks)

The screening test results are checked to see whether the candidate conforms to the selection/exclusion

criteria for registration as a subject in the clinical trial.

⑤ Final assessment based on the selection/exclusion criteria
⑥ Assignment of registration number
⑦ Blood collection for investigational drug production (cell preparation)
⑧ Immune reaction examination
⑨ Scheduling of next visit
⑩ Administration of an anticancer agent (not on days when AKC is injected)

7.12.2.3 Visit 3 (0 week)

The investigational drug shall be administered for the first time during this visit, and the following types of assessment shall be carried out:

⑩ Investigation of medication history
⑪ Vital signs: Blood pressure, pulse, body temperature
⑫ Physical examination: External appearance, skin, head and neck, chest and lungs, heart, abdomen, urinary and reproductive systems, limbs, musculoskeletal system, nervous system, lymph nodes and other body organs
⑬ Clinical laboratory tests: Hematological test, hematobiochemical test, and urine test
⑭ KPS, QLQ-C30 (Ver 3.0) Korean examinations
⑮ Blood collection for investigational drug production (cell preparation)
16 Administration of the investigational drug
17 Check for adverse events (at every visit)
18 Scheduling of next visit

7.12.2.4 Visit 4 ~ Visit 37 (1 ~ 6 weeks, 8 ~ 68 weeks)

The investigational drug shall be administered during these visits, and the following types of assessment shall be carried out:

⑪ Investigation of medication history (at every visit)
⑫ Vital signs: Blood pressure, pulse, body temperature (at every visit)
⑬ Physical examination: External appearance, skin, head and neck, chest and lungs, heart, abdomen, urinary and reproductive systems, limbs, musculoskeletal system, nervous system, lymph nodes and other body organs (at every visit)
⑭ Clinical laboratory tests: Hematological test, hematobiochemical test, and urine test (at every odd-numbered visits from Visit 3)
Autoimmune reaction examination (Visit13, Visit 27, Visit 37)
⑮ KPS, QLQ-C30 (Ver 3.0) Korean examinations (at every odd-numbered visits from Visit 3)
16 Blood collection for investigational drug production (excl. visits 5, 8, 11, 14, 17, 20, 23, 26, 29, and 32)
17 Administration of the investigational drug (excl. visits 6, 9, 12, 15, 18, 21, 24, 27, 30, 33)
18 Administration of an anticancer agent (not on days when AKC is injected)
19 Tumor evaluation (at visits 9, 15, 21, 27, 33, 37)
20 Immune reaction examination (at visits 21 and 37): Immune reaction examination to assess the response to the anticancer therapy
21 Check for adverse events (at every visit)

7.12.2.5 Termination visit

Where 9.1 Subject withdrawal (drop-out) and trial termination criteria apply, a final visit for termination or discontinuation of the clinical trial shall be scheduled, and the following types of assessment shall be carried out during the visit:

⑧ Investigation of medication history

⑨ Vital signs
⑩ Physical examination
⑪ Clinical laboratory tests: Hematological test, hematobiochemical test, and urine test
⑫ KPS, QLQ-C30 (Ver 3.0) Korean examinations
⑬ Tumor evaluation (only if it is at F/U that occurs every three months)
⑭ Immune reaction examination
⑮ Check for adverse events
16 Administration of an anticancer agent (not on days when AKC is injected)

7.12.2.6 Visit for checking survival
After the clinical trial is terminated for a reason other than withdrawal of consent, the survival information on the subjects shall be collected once every 4 weeks in person or by phone after the termination visit.
④ Survival information
⑤ Tumor evaluation (only if it is at F/U that occurs every three months)
⑥ Check for adverse events

7.12.2.7 Additional visit
Additional visits may be scheduled at any time when it is deemed necessary by the investigator or when it is requested by the subject or his/her legal representative.

8. User precautions for the medicinal products used in the clinical trial
The investigational drug in this clinical trial will be applied to the human body for the first time, and the potential adverse events that are expected are follows:
5. Cold-like symptoms such as fever, chills, and headache
6. Anaphylaxis: Cold sweat, skin flare, urticaria, dyspnea, etc.
7. General caution: Use of the investigational drug by those other than the subject is strictly prohibited.
8. Administration to pregnant and lactating women, newborns, infants and young children: A safety and effectiveness study has not been conducted on pregnant and lactating women, newborns, infants and young children
9. Bone marrow hypofunction (exacerbated blood test abnormality) is most severe (may vary depending on the type of anticancer agent administered) three to four weeks after initiating chemotherapy. In such cases, the administration of the anticancer agent may be postponed or the dose may be decreased at the discretion of the investigator. Other adverse reactions include nausea, vomiting and hair loss.

9. Withdrawal and termination criteria and criteria for exclusion from analysis

9.1 Subject withdrawal (drop-out) and trial termination criteria
For all of the subjects participating in the clinical trial, whether they have completed the trial or not shall be recorded. If the drug administration or observation has been discontinued, the reason shall be recorded. A subject may withdraw or be removed from the clinical trial for any of the following reasons:
⑫ The subject disqualified based on the selection/exclusion criteria
⑬ A serious adverse event has occurred to the subject or the subject has requested to discontinue due to an adverse event
⑭ The subject who is found to have a systemic disease that was not discovered in the screening test

⑮ The subject (or his/her guardian) withdrew his/her consent to participate in the clinical trial
16 A serious violation of the protocol is committed by the principal investigator, a sub-investigator, or the subject in question
17 The subject does not recover from an adverse event
18 The subject cannot be tracked
19 There is a problem in administering the investigational drug to the subject
20 The subject has taken a drug, etc. that may affect the results of the study without any directions from the doctor in charge during the administration period or follow-up observation period
21 It is deemed by the principal investigator or sub-investigator that the study is not proceeding in an appropriate manner
22 The administration of the investigational drug has been postponed or cancelled three consecutive times or more during the clinical trial period or postponed or cancelled four times or more in total

9.2 Compliance in the clinical trial and handling a protocol violation

The principal investigator and sub-investigator of this clinical trial shall fully familiarize themselves with and observe this clinical trial protocol to prevent any violations thereof. For the purpose of complying with the investigational drug administration and test/examination schedules, the sub-investigator shall take the appropriate measures for the subjects to make an outpatient visit as scheduled and such measures may include notifying them in writing or by phone, for example. If an unavoidable violation of the protocol has occurred, it shall be dealt with as follows:

In the event of a critical violation of the protocol, the subject in question shall be excluded from analysis (excl. PP), in principle.

③ Failure to obtain written consent

④ Does not the selection criteria or falls under the exclusion criteria

As for other minor violations of the clinical trial protocol that are considered to have no effect on the analysis of the study results, the severity of the violation or extent of delay and the reason behind the violation/delay shall be accurately described. The investigator, monitor, and statistician shall examine its impact on the study and include the information in the PP analysis.

10. Effectiveness assessment criteria, assessment method and analysis method (statistical analysis method)

10.1 Assessment criteria

The variables for the safety and effectiveness assessment for assessing the results of the clinical trial are as follows:

10.1.1 Safety assessment variables

④ Adverse reactions: Fever, subjective and/or objective symptoms, causal relationship with the investigational drug, etc.

⑤ Clinical laboratory tests

⑥ Vital signs and physical examination

10.1.2 Effectiveness assessment variables

⑥ Progression-free survival

⑦ Overall survival

⑧ Treatment response rate according to the tumor evaluation

⑨ QLQ-C30 (Ver 3.0) Korean

⑩ Karnofsky performance status (KPS)

10.2 Statistical analysis method

10.2.1 General principles for results analysis

The data obtained from the subjects through this clinical trial are largely divided into three groups: Safety, Full Analysis (FA) and Per-Protocol (PP). The main population of analysis in this clinical trial is the FA group, while additional analysis is performed on the PP group and safety is analyzed based on the Safety group. However, in the case of the FA group for the primary effectiveness assessment, the analysis group shall be redefined by setting forth the exclusion criteria for the subjects taking into consideration of the characteristics of the assessment variables. The results of all three groups shall be compared, and when the results differ among the groups, the results of each analysis method shall be presented with the reasons for the differences described in detail. All of the analyses shall be performed at a significance level of 5%.

Assignment to the FA Group:

Those who have been administered the investigational drug at least once after being registered as a subject in this clinical trial and had the variables for the effectiveness assessment measured at least once after the administration shall be included in the analysis. For the FA group, the intention-to-treat (ITT) shall be a fundamental principle.

However, the FA group for the primary effectiveness assessment, the following subjects shall additionally be excluded.

⑮ A subject who no longer meets the selection criteria or meets the exclusion criteria

16 A subject who has never been administered the drug used in the clinical trial after registration in the study

17 A subject without any measurement values for effectiveness assessment after registration in the study

<u>Assignment to the PP Group:</u>
The PP group is comprised of subjects included in the FA group that have completed the clinical trial in accordance with the clinical trial protocol. However, subjects falling under any of the following shall be excluded:

⑫ A subject whose investigational drug administration has been postponed or cancelled two or more times in each cycle during the clinical trial period

⑬ A subject whose investigational drug administration has been postponed or cancelled three consecutive times or more or four or more times in total during the clinical trial period

⑭ A subject who did not recover from an adverse event even after 4 weeks

⑮ A subject who dropped out or was removed from the clinical trial before fulfilling the duration specified in the protocol

18 A subject who no longer meets the selection criteria or meets the exclusion criteria

16 Any other cases that could be considered a serious violation of the protocol

<u>Assignment to the Safety Group:</u>
Those who have been administered the investigational drug at least once shall be included in the analysis.

<u>Handling of missing values</u>
If there is a missing value at any point or a missing value occurs due to a subject dropping out or being removed before the completion of the clinical trial, the missing data shall be substituted using the Last Observation Carried Forward (LOCF) method.

Interim Analysis
1) Purpose of the Interim Analysis
The research team has planned an interim analysis for the purpose of checking for any correlation between the administration of the investigational drug and the death cases and extended survival period by performing an analysis of the 14 subjects after completing an assessment of 14 of the 23 subjects. The aim is to assess the futility of using the investigational drug and to decide on whether to continue with the clinical trial, based on the results of the interim analysis. If the results indicate that it is futile to use the investigational drug, the clinical trial shall be terminated after completing the program for the current subjects, without registering any additional patients. In contrast, if it is not deemed to be futile, then the clinical trial shall be continued until completion and an additional clinical trial plan will be set forth for glioblastoma patients.

2) Method of the Interim Analysis
The interim analysis shall be carried out after completing the necessary assessment for 14 of the 23 subjects. The interim analysis and the final analysis shall be performed on the variables in the safety assessment, which are adverse reactions, clinical trial lab test results, measurements of vital signs, and physical examination results, as well as the variables in the efficacy assessment such as OS, PFS, response rate, QLQ-C30 (ver 3.0) Korean, KPS (Karnofsky performance status), and immune reaction evaluation. The assessment items and statistical analysis method applied to the interim analysis and the final analysis shall be identical. The final analysis shall be conducted after the completion of F/U for all subjects.

3) Multiplicity Test

Adjustment for multiplicity is not needed for this study, as it is a Phase 1/2 exploratory clinical trial.

4) Assessment Criteria and Follow-up Measures
The final decision on whether to continue on with the study, based on the results of the interim analysis, shall be made by the investigator, based on the direction of the clinical trial presented after an analysis of the safety and therapeutic effects of the investigational drug.
As for the futility assessment, it will be deemed that the use of the investigational drug is futile in the event of a serious adverse event in relation to the investigational drug or the mOS is lower than the weighted average (8.3 months) of the therapeutic agents reported in the literature. If the results of the interim analysis indicate the futility of the use of the investigational drug, the clinical trial shall be terminated after completing the program for the current subjects, without registering any additional patients. If not, the clinical trial shall be continued according to the protocol.

10.2.2 Demographic data
In the case of continuous data, the descriptive statistics (mean, standard deviation, minimum value, maximum value, etc.) shall be presented according to the time of visit, while in the case of categorical data, the frequency and ratio for each category shall be presented.

10.2.3 Safety data

10.2.3.1 Adverse events
In this clinical trial, adverse events shall be classified into 1) adverse events prior to administration of the investigational drug (i.e. underlying symptoms and signs) and 2) treatment emergent adverse events (TEAE). TEAE shall be defined as ① an adverse reaction that occurred for the first time after the administration of the investigational drug and ② an adverse event in which the severity of underlying symptoms and signs that have occurred before the administration of the investigational drug has worsened after the administration. In the analysis of the adverse events, TEAEs shall be analyzed and all of the adverse events shall be presented as a list.
In case of multiple occurrences of the same adverse event to a subject, the event shall be regarded as a single adverse event. If the severity of the same adverse event differs, the worst severity shall be noted. If the causality of the same TEAE in a subject is different, the causality that is most relevant to the investigational drug shall be noted.
The percentage of subjects who experienced an adverse event and the 95% confidence interval in relation thereto shall be presented. All of the adverse events that have occurred shall be documented according to the level of severity. The adverse events and serious adverse events associated with the investigational drug shall also be documented.

10.2.3.2 Clinical laboratory tests
In the case of continuous data such as the hematological or hematobiochemical test results, the descriptive statistics (mean, standard deviation, minimum value, maximum value, etc.) shall be presented, whereas in the case of categorical data such as the urine test and 12-lead ECG results, the frequency and ratio for each category shall be presented.
For continuous variables, a paired t-test or Wilcoxon signed-rank test shall be performed on the differences between before and after the administration of the investigational drug to examine the differences between the visits. McNemar's test shall be carried out for categorical variables. In addition, clinically significant non-normalized rates compared to before treatment shall be presented.

10.2.3.3 Vital signs and physical examination
The descriptive statistics for vital signs (blood pressure, pulse, and body temperature) shall be presented, and a shift table indicating the changes between before and after the administration of the investigational drug with respect to the normal and abnormal values measured in the physical examination shall be presented. The before and after differences shall be analyzed by a paired t-test or Wilcoxon signed-rank test for continuous variables and the McNemar's test for categorical variables.

10.2.4 Effectiveness data

Progression-free survival (PFS)
PFS shall be estimated by the Kaplan-Meier method, and the median survival time shall be calculated.

Overall survival (OS)
OS shall be estimated by the Kaplan-Meier method, and the median survival time shall be calculated. OS shall be the period between the date on which the individual was registered as a subject of this clinical trial and assigned a number and the date on which he/she died, irrespective of the cause.

Response rate
The degree and percentage of the subjects who showed a response to treatment shall be presented, with the 95% confidence interval indicated.

QLQ-C30 (Ver 3.0) Korean
The measurements for each item shall be compared in order to determine the level, and the changes according to the progress of treatment shall be analyzed. The descriptive statistics (mean, standard deviation, median, minimum value, and maximum value) shall be presented with respect to the QLQ-C30 (Ver 3.0) Korean evaluation scores and be tested using an independent t-test or Wilcoxon's rank sum test depending on the presence of normality. As for making a comparison between before and after the administration of the investigational drug, a paired t-test or Wilcoxon's signed-rank test shall be performed.

Karnofsky performance status (KPS)
The measurements for each item shall be compared in order to determine the level, and the changes according to the progress of treatment shall be analyzed. The descriptive statistics (mean, standard deviation, median, minimum value, and maximum value) shall be presented with respect to the KPS evaluation scores and be tested using an independent t-test or Wilcoxon's rank sum test depending on the presence of normality. As for making a comparison between before and after the administration of the investigational drug, a paired t-test or Wilcoxon's signed-rank test shall be performed.

Evaluation of immune reaction
Statistical analysis of the immune reaction evaluation: The analysis can be carried out by a repeated measure of ANOVA in relation to the change from baseline or measured value, depending on the nature of the variable, or the X2 test or Fisher's exact test may be applied to compare the frequency of certain levels between the groups or immune reactions.

11. Safety assessment method and criteria and reporting method incl. adverse events

11.1 Terms related to safety and definitions thereof

1) Adverse Event (AE)
Any unfavorable and unintended sign (e.g. an abnormal laboratory finding), symptom, or disease temporally associated with the use of a medicinal product, whether or not considered related to the medicinal product

2) Adverse Drug Reaction (ADR)
All noxious and unintended responses to a medicinal product related to any dose, with a reasonable possibility that there is a causal relationship between a medicinal product and an adverse event

3) Serious and Unexpected Adverse Drug Reaction
An unexpected adverse drug reaction is one where the nature or severity of which is not consistent with the applicable product information (e.g. Investigator's Brochure or attached documents for the medicinal product) and expedited reporting is required in the event of a serious and unexpected adverse drug reaction.

4) Serious AE/ADR

A serious adverse event or adverse drug reaction occurring at any arbitrary dose of the medicinal product used in the clinical trial that falls under any of the following:

⑦ results in death during the clinical trial period;

⑧ is life-threatening (the patient was at risk of death at the time of the event/reaction);

⑨ results in persistent or significant disability or incapacity;

⑩ requires inpatient hospitalization or prolongation of existing hospitalization (however, hospitalization scheduled for administration of an anticancer agent or radiotherapy or hospitalization for less than 24 hours is not considered a serious AE in this clinical trial);

⑪ leads to a congenital anomaly/birth defect;

⑫ any other medically important situations.

Upon occurrence of a situation that is deemed to cause a serious effect on the patient's health condition and well-being, even if it does not fall under any the above-listed situations, a decision shall be made on whether to consider it as a serious AE/ADR, based on the medical judgment of the doctor in charge and relevant experts, and an appropriate measure shall be taken accordingly.

11.2 Assessment method

1) An AE is a symptom that has not been observed prior to participation in the clinical trial but newly arises during administration of the investigational drug. Such includes unintended signs (incl. an abnormal laboratory finding), which does not necessarily have to have a causal relationship with this treatment, as well as symptoms, or temporary phenomena related to the use of the investigational drug.
2) Any untoward clinical signs and symptoms that occur prior to the administration of the investigational drug after the screening process shall be recorded in the current medical history, but not recorded as AE.
3) If a subject becomes pregnant during the clinical trial period, it will not be regarded as a serious AE/ADR, but she shall drop out from the clinical trial. The information on the subject and her fetus shall be tracked and reported until delivery.
4) Any and all phenomena (signs, symptoms, onset, duration, etc.) expected as an AE from the investigational drug must be recorded in the AE report. Those that are not recorded shall be classified as subjective symptoms.
5) The degree of the adverse reaction shall be assessed by the sub-investigator in reference to the assessment criteria [CTCAE] according to severity of the symptoms.
6) The causal relationship with the investigational drug shall be assessed by the doctor in charge by classifying it into one of the six stages according to the assessment criteria (see Section 11.4).

11.3 Assessment criteria

11.3.1 Fever

For fevers, the body temperature shall be measured and it shall be classified according to the following assessment criteria:

Grade level	Grade 0	Grade 1	Grade 2	Grade 3	Grade 4
Fever (°C)	none	< 38.7	38.7- 40	> 40.0	Fever with hypotension

11.3.2 Subjective and objective symptoms

The degree of subjective and objective symptoms shall be assessed by classifying them according to the criteria proposed for abnormalities in the examined items (CTCAE; Version 4.0) through a medical examination by the sub-investigator.

CTCAE 5-step classification method	None	Mild	Moderate	Severe	Life-threatening consequence	Death

11.4 Causal relationship with the investigational drug

In the event of an AE, the relationship with the investigational drug shall be classified as follows by the investigator, and the comments and opinions of the sub-investigator shall be attached, if necessary.

① Definitely related
- There is evidence that the drug has been administered;
- The AE is most plausibly explained by the administration of the drug in question than any other reasons;
- The AE disappears once the drug administration is discontinued;
- The rechallenge (performed only when possible) result is positive;
- The AE is consistent with the information already known about the drug or a similar line of drugs.

② Probably related
- There is evidence that the drug has been administered;
- The drug administration and the AE have a reasonable time sequence;
- The AE is more plausibly explained by the administration of the drug in question than other reasons;
- The AE disappears once the drug administration is discontinued.

③ Possibly related
- There is evidence that the drug has been administered;
- The drug administration and the AE have a reasonable time sequence;
- The AE is deemed to have possibly caused by the drug in question with the same likelihood as other potential causes;
- The AE disappears once the drug administration is discontinued.

④ Probably not related
- There is evidence that the drug has been administered;
- There is a more plausible cause behind the AE;
- The result of discontinuing the drug administration (if discontinued) is negative or ambiguous.

⑤ Definitely not related
- There is no evidence that the drug has been administered;
- The AE is most plausibly explained by another reason;
- The AE does not disappear as a result of discontinuing the drug administration (if discontinued).

⑥ Unknown

11.5 Reporting method

11.5.1 Reporting adverse events

7) The principal investigator shall educate the sub-investigator, subjects and/or their guardians on all potential AEs that may occur after the administration of the investigational drug and instruct them to report any and all phenomena they experience after the administration.
8) The type, onset, degree, treatment measure, medication, progress, causal relationship with the investigational drug, etc. of any systemic or clinicopathologic symptoms occurring after the

use of the investigational drug shall be recorded and retained in accordance with the clinical trial management standards by documenting the information in the case records.

9) The principal investigator shall describe and assess all the symptoms that occurred during the clinical trial period when reporting the clinical trial results. In the event of a serious adverse drug reaction (ADR) during the trial, it shall be reported to the IRB so that the decision on whether to continue or discontinue the trial may be made. Where expedited reporting is necessary, the Minister of Food and Drug Safety shall be reported within the period set forth as follows:

10) A serious ADR refers to a case in which the causal relationship with the investigational drug cannot be ruled out and it may be any one of the following cases:
   ① results in death during the clinical trial period;
   ② is life-threatening (the patient was at risk of death at the time of the event/reaction);
   ③ results in persistent or significant disability or incapacity;
   ④ requires inpatient hospitalization or prolongation of existing hospitalization (however, hospitalization scheduled for administration of an anticancer agent or radiotherapy or hospitalization for less than 24 hours is not considered a serious AE in this clinical trial);
   ⑤ leads to a congenital anomaly/birth defect;
   ⑥ any other medically important situations.

11) Additional safety information shall be reported periodically until the ADR ceases to occur (e.g. the ADR disappears or it is impossible to conduct a follow-up examination).

12) The principal investigator shall carry out the clinical trial in conformance with the Declaration of Helsinki and KGCP.

11.5.1.1 Expedited reporting

1) Purpose

The purpose of expedited reporting is to ensure that the Ministry of Food and Drug Safety (MFDS), investigators, and other related parties are aware of the new and important information about serious adverse drug reactions.

2) Matters requiring expedited reporting

① Single cases of serious and unexpected ADR

② Where prompt communication with the MFDS is necessary

Appropriate medical and scientific judgment should be applied for each situation. In general, information that might materially influence the benefit-risk assessment of a medicinal product or that would be sufficient to consider changes in medicinal product administration or in the overall conduct of a clinical investigation represents such situations. Examples include:

a. For an "expected," serious ADR, an increase in the rate of occurrence which is judged to be clinically important.

b. A significant hazard to the patient population, such as lack of efficacy with a medicinal product used in treating life-threatening disease.

c.) A major safety finding from a newly completed animal study (such as carcinogenicity).

3) Reporting time frames

In the event of a serious and unexpected ADR during the clinical trial, the principal investigator shall:

• report to the IRB in accordance with the standard operating procedure (SOB) of the IRB, and report any additional safety information until the ADR ceases to occur (e.g. the ADR disappears or it is impossible to conduct a follow-up examination);

• report to the person-in-charge in the Clinical Research Team of CHA Biotech, which is the supplier of the investigational drug, within 24 hours of becoming aware of the ADR, and report any additional safety information until the ADR ceases to occur;

• promptly report to the Minister of Food and Drug Safety within the period prescribed hereunder:

③ Unexpected ADRs that are fatal or life-threatening shall be reported promptly. The principal investigator shall report to or have the sub-investigator report to the Minister of Food and Drug Safety as soon as possible but no later than 7 calendar days after the case was first brought to his/her attention by phone or fax or in writing, and submit a complete report within 8 days of the initial report.

④ All other serious and unexpected ADRs shall be reported by the principal investigator within 15 days after receiving a report thereon or becoming aware of it.

4) Minimum criteria for initial reporting

Information for the final description and evaluation of an initial report may not be available within the aforementioned time frames for reporting. However, an initial report must be submitted within the prescribed time above and must include the following categories of information:

⑤ Subject in whom the ADR occurred;

⑥ Suspected drug;

⑦ Information provider;

⑧ Serious and unexpected ADR.

Detailed follow-up information shall be obtained and submitted for use.

5) Reporting method

For expedited reporting of ADRs, the Serious Adverse Drug Reaction Report Form of the Ministry of Food and Drug Safety shall be used. The utmost effort shall be made to obtain as much information as possible, even if all the information is not available at the time of expedited reporting.

11.6 Measures to be taken for serious ADRs

During the clinical trial period, the principal investigator and the sub-investigator must make every effort to ensure the safety of the subjects. In the event of a serious ADR, the trial shall be suspended to take prompt and appropriate measures so as to minimize the ADR.

The duties of each person in charge in the event of a serious ADR during the clinical trial are as follows:

1) Duties of the principal investigator

The principal investigator shall, in the event of a serious ADR during the clinical trial, report it immediately in accordance with the SOP of the IRB, and entirely or partially suspend the clinical trial involving the investigational drug in question until further instructions are given.

① Unexpected ADRs that are fatal or life-threatening shall be reported promptly. The principal investigator shall report to or have the sub-investigator report to the Minister of Food and Drug Safety as soon as possible but no later than 7 calendar days after the case was first brought to his/her attention by phone or fax or in writing, and submit a complete report within 8 days of the initial report.

② All other serious and unexpected ADRs shall be reported by the principal investigator within 15 days after receiving a report thereon or becoming aware of it.

2) Duties of the sub-investigator

The sub-investigator shall, in the event of a serious ADR during the clinical trial, report it immediately to the principal investigator.

3) Duties of the IRB

The IRB shall, in the event of a serious ADR, have the principal investigator take necessary measures such as commanding that the clinical trial be suspended entirely or partially.

12. Written consent, compensation agreement, and post-trial medical examination and

treatment of the subjects

12.1 Consent form and written explanations

The principal investigator or sub-investigator shall provide a sufficient explanation of the details of the study, effects of the investigated drug, and possible adverse events to the subjects (or their legal representatives) before initiating the study and obtain written consent from the subjects. The investigators shall keep the original copy of the signed consent form as part of the clinical trial records and provide a photocopy of the signed consent form and written explanations to the subjects (or their legal representatives).
See Attachment 2 Consent Form and Explanations for the Subjects

12.2 Agreement on compensation

See Attachment 3 Agreement on Victim Compensation

12.3 Post- trial medical examination and treatment of the subjects

Subjects dropping out of the clinical trial or unresponsive to the investigational drug shall be instructed to seek other types of appropriate treatment. Subjects who have complete the clinical trial shall be instructed to receive medical examination anytime, according to the instructions of the doctor in charge, in preparation for unexpected and delayed adverse events.

13. Measures for ensuring the safety of the subjects

13.1 Clinical trial institution

The head of the clinical trial institution shall have the clinical laboratory, equipment, and specialized manpower necessary for carrying out each phase of the clinical trial in question, and strive for perfection when making the preparations to ensure the proper execution of the clinical trial.

13.2 Approval of and amendments to the clinical trial protocol

In case of planning to obtain approval of the clinical trial protocol or amend the approved clinical trial protocol, IRB's approval for each phase of the clinical trial or the amended protocol shall be obtained, and the approval of the Minister of Food and Drug Safety shall be obtained, if necessary. Subjects cannot be enrolled in the clinical trial for participation prior to approval.

13.3 Familiarization of the clinical trial protocol

This clinical trial has been prepared with the rights and welfare of the subjects in mind, based on the Declaration of Helsinki and KGCP. As such, the principal investigator and sub-investigators shall accurately analyze and familiarize themselves with the clinical trial protocol and actively respond to the problems concerning the subjects in the clinical trial.

13.4 Consent to participate in the clinical trial

Before initiating the clinical trial, the subjects shall be explained of all matters in relation to the details of the clinical trial, effects of the investigational drug, adverse events, and safety, and their voluntary consent to participate in the study shall be obtained.

13.5 Accurate selection of subjects

Prior to the clinical trial, the suitability of the subjects shall be thoroughly assessed through a sufficient interview and tests/examinations.

13.6 Checking the progress of the clinical trial

The principal investigator shall periodically report to the sponsor the adverse events, progress of the trial, situation, results, etc., and the clinical trial sponsor shall periodically review the progress of the clinical trial.

13.7 Monitoring of the clinical trial institution

Monitoring shall be performed to check whether the rights and welfare of the subjects are protected, the data reported in relation to the clinical trial are accurate, complete and verifiable in comparison with the evidentiary documents, and whether the clinical trial is carried out in accordance with the approved protocol, Clinical Trial Management Standards for Medicinal Products and Subparagraph 8 Clause e) of the Enforcement Rule.

Monitoring of the clinical trial shall be performed by a monitor requested to perform the monitoring work through regular visits to the clinical trial institution and by phone. During a visit, the monitor shall check the original copies of the records on the subjects, drug management records, and records retention (research file). Also, the monitor shall review the progress of the clinical trial and speak to the investigators in case of a problem.

The appropriate time frame for these visits shall be scheduled in a distributed manner through consultation between the investigators and the monitor. The investigators shall also make source documents (hospital or patient charts, records of the laboratory test results, appointment records, etc.) of the subjects available to the monitor so that the data and information entered into the case records can be checked, as defined in the Clinical Trial Management Standards for Medicinal Products.

13.8 Confidentiality

Any records with which the subjects can be identified shall be kept confidential, even when publishing the results of the clinical trial. The specific details in relation to confidentiality are as follows:

The monitors and inspectors concerned in this clinical trial may view the records of the subjects for the purpose of monitoring and checking the clinical trial and managing the progress thereof. The investigators shall be aware that, with the conclusion of the agreement on this trial, the monitors and inspectors from the institution commissioning this clinical trial may review or photocopy the subjects' charts and case records in order to verify their content. Such information must be kept confidential and the necessary facility and management standards must be in place to ensure confidentiality.

On the other hand, all documents related to the clinical trial such as case records shall be recorded and identified with the subject identification codes (generally the subjects' initials), not their names.

13.9 Measures taken in the event of an adverse event

In the event of an adverse event, the subject in question shall receive the necessary examination and treatment from the doctor in charge. In case of a serious adverse drug reaction, the clinical trial shall be suspended and prompt and appropriate measures shall be taken according to the prescribed matters.

13.10 Data and Safety Monitoring Board (DSMB)

14. Matters necessary for conducting the clinical trial in a safe and scientific way

There shall be a separate location for storing various materials and records related to the execution of the clinical trial, and the security thereof shall be well-maintained. After completing the results report, a person responsible for retaining the related materials and records shall be designated so that they can be retained for three years from the end date of the clinical trial.

15. References

1) Standards of care for treatment of recurrent glioblastoma--are we there yet? Neuro Oncol. 2012 Nov 7. [Epub ahead of print]

2) Radiotherapy plus concomitant and adjuvant temozolomide for glioblastoma. N Engl J Med. 2005 Mar 10; 352(10): 987-96.

3) Human interferon beta, nimustine hydrochloride, and radiation therapy in the treatment of newly diagnosed malignant astrocytomas. Journal of Neuro-Oncology. 2005;72: 57-62.

4) Randomized phase II study of temozolomide and radiotherapy compared with radiotherapy alone in newly diagnosed glioblastoma multiforme. J Clin Oncol. 2005 Apr 1; 23(10): 2372-7.

4) Central nervous system cancers: Clinical practice guidelines Vol. 2, 2005. National Comprehensive Cancer Network.

5) Health-related quality of life in patients with glioblastoma: a randomized controlled trial. Lancet Oncology 2005; 6: 937-44.

6) Designs for group sequential tests. Fleming, T.R., Harrington, D.P., & O'Brien, P.C. Controlled Clinical Trials 1984;. 5:348-361.
7) A multiple testing procedure for clinical trials. O'Brien, P.C., & Fleming, T.R. Biometrics. 1979; 35:549-556.
8) Autologous Natural Killer Cell Therapy for Human Recurrent Malignant Glioma. Anticancer Res.2004; 24:1861-1872.
9) Infusion of the allogeneic cell line NK-92 in patients with advanced renal cell cancer or melanoma: a phase 1 trial. 2008;10, No 6: 625-632
10) Survival of Patients with Recurrent Malignant Glioma Treated with Temozolomide. Drug R&D 2003;4(5) :285-291
11) The clinical efficacy of combination of docetaxel and temozolomide in previously treated patients with stage IV melanoma research.2010 ;Vol 20 :43-47

5. Summary of Protocol Amendments

No.	Version	Date	Approval date	Summary of changes
**1**	Ver.1.0	11.30.2012	Approval after correction	Application for initial review
**2**	Ver.1.1	01.04.2013	01.23.2013	6.2 Investigational drug management and records<br>- Added the matters regarding the manufacture and supply procedures and the records pertaining to the investigational drug for adequate management of the investigational drug<br><br>7.8 Method and duration of administration<br>- Changed the maximum dose of the cellular immunotherapy agent from "24" to "12"<br>- Included a related paper, Weller M. Neuro Oncol. 2012 Nov 7 [can be checked in 15. References 1)]<br>- Reflected the above changes in 7.12.1 Clinical trial schedule and 7.12.3 Clinical trial method by visit
**3**	Ver.1.2	06.04.2013	07.24.2013	[minor change]<br>- Changed the term, "tester," to "investigator" to ensure the use of uniform terminology<br>- Changed the term, "self-activated lymphocyte," to "autologous activated lymphocyte" to ensure the use of standard terminology<br><br>6.1 Investigational drug<br>- Changed the dose of the cell therapy agent from "1 x 109 cells or higher" to "2~6 x 109 cells"<br><br>7.6 Clinical trial design<br>- Changed the matters regarding the dose limiting toxicity test planned to assess subject safety by deciding to proceed with the safety assessments through the Safety Data Monitoring Committee [See Attachment 7. AKC-DSMB Model Charter] and accordingly, 7.12.2.10 Dose limiting toxicity was deleted<br><br>7.8 Method and duration of administration<br>- The new dose for the cell therapy agent was reflected in this section<br>- When collecting blood to manufacture the cell therapy agent, there may be difficult in the manufacturing process as the blood may not be in an appropriate state for manufacture or an anticancer therapy may be administered in combination. Accordingly, the criteria for the administration of the cell therapy agent and termination (disqualification) were added.<br><br>7.10 Drugs that can be used in combination<br>- Although all kinds of drugs may be administered in combination in this clinical trial, anticancer agents that may be administered are restricted to those used for BCNU and PCV therapy

No.	Version	Date	Approval date	Summary of changes
**4**	Ver.1.3	08.19.2013	Approval after correction	7.12 Matters for observation and observation method<br>- Clearly described matters concerning the testing of vital signs, physical examination and tests carried out to assess the response to the anticancer therapy (timepoint, judgment criteria, etc.)<br><br>9. Withdrawal and termination criteria and criteria for exclusion from analysis<br>- Clearly described the withdrawal and termination criteria
**5**	Ver.1.3	11.08.2013	11.29.2013	7.12 Matters for observation and observation method<br>- Clearly distinguished the autoimmune response test and the tests carried out to assess the response to the anticancer therapy in the description
**6**	Ver.1.4	01.27.2014	02.12.2014	[minor change]<br>- Of the anticancer therapy response assessment items, "C4" and "C8" were corrected to "CD4" and "CD8," respectively
**7**	Ver.1.5	02.10.2014	02.26.2014	[minor revision]<br>- Added the information on the co-investigators
**8**	Ver.1.51	04.17.2014	05.14.2014	[minor revision]<br>- Added the information on the investigational drug manager<br><br>[minor change]<br>- Changed the Korean term used in reference to the "subject" in conformance with the revised terminology of the Korean Good Clinical Practice (KGCP) (not a change in the meaning of the word)
**9**	Ver.1.6	08.27.2014	09.11.2014	[minor change]<br>- In order to ensure uniform terminology, "physical test" was changed to "physical examination" and "anticancer therapy" was changed to "administration of anticancer agent"<br><br>[minor change]<br>- Corrected the erroneous entries in the tests/examinations<br>- Eliminated the inconsistencies between the clinical trial schedule and some of the tests/examinations described in the clinical trial method
**10**	Ver.1.7	09.18.2014	10.15.2014	7.8 Method and duration of administration<br>- It was prescribed that in case of a problem in the manufacture of the investigational drug (due to failure in culturing or infection, etc.), the investigational drug be administered at the next scheduled visit, instead of the current visit<br>- This is a measure implemented to prevent delays in the administration of the anticancer agent to ensure the safety of the subjects
**11**	Ver.1.8	11.04.2014	11.28.2014	[minor change]<br>- In order to ensure the use of uniform terminology, "patient" was changed to "subject"

No.	Version	Date	Approval date	Summary of changes
				7.8 Method and duration of administration<br>- Added a description of the standards concerning the postponement and cancellation of the administration of the investigational drug to ensure appropriate treatment and facilitate
**12**	Ver.2.0	04.15.2015	04.15.2015	7.12 Matters for observation and observation method<br>- Deleted CD4 and CD8 among the immune response examination items
**13**	Ver.3.0	05.27.2015	05.27.2015	7.8 Method and duration of administration<br>- Added the method of increasing the amount of blood collected to be frozen for preservation, due to the possibility of reduced immune cell activity caused by the anticancer therapy carried out during the subjects' participation in the clinical trial that may in turn result in failing to meet the expected level of effectiveness. Also, it was to prevent the subjects from experiencing discomfort from blood collection at every visit.<br><br>7.10 Drugs that can be used in combination<br>- Added "Nolvadex Tab" as an anticancer drug that can be used in combination<br>- Changed the anticancer therapy schedule based on the added method of administering the anticancer agent<br>- The newly added adverse effects of the anticancer agent were reflected in 8. User precautions for the medicinal products used in the clinical trial<br><br>11. Safety assessment method and criteria and reporting method incl. adverse events<br>- Clarified the procedure for reporting adverse events to the IRB and the relevant authorities in accordance with the related laws and regulations, as this clinical trial is led by the investigators (not a sponsor) (removed the "duty to report to the sponsor")
**14**	Ver.4.0	08.06.2015	08.26.2015	[minor change]<br>- Corrected the name of the GMP (Bundang CHA Hospital GMP → CHA Biotech GMP)<br>- Made changes to the names of the main components of the raw materials (autologous activated lymphocyte → autologous blood-derived activated lymphocyte)<br><br>7.12 Matters for observation and observation method<br>- The expression of TNF-α, IL-1 and IFN-γ is not checked by flow cytometry during the immune response assessment and thus the related text was deleted
**15**	Ver.4.1	12.07.2015	01.13.2016	- Changed the autoimmune response examination period to carry out additional examinations at the 8th, 18th and 24th visits after screening to ensure the safety of the subjects, based on a recommendation of the Ministry of Food and Drug Safety<br>- Clarified the matters concerning the management of the investigational drug (responsibility for acquisition, storage, management and return of the investigational drug)

No.	Version	Date	Approval date	Summary of changes
	Ver.4.2	12.30.2015	01.13.2016	7.12 Matters for observation and observation method<br>- Provided a clear description of the clinical trial method for each visit to ensure accuracy and described the detailed items of the tests/examinations
**16**	Ver.5.0	03.08.2016	03.21.2016	7.10 Drugs that can be used in combination<br>- Added ACNU as an anticancer agent that can be administered in combination<br>- Changed the anticancer therapy schedule based on the newly added methods of administering the anticancer drug
**17**	Ver.6.0	01.12.2017	02.27.2017	[minor revision]<br>- Deleted the information on the co-investigators<br><br>10.2.4.1 Variable in the efficacy assessment: Progression-free survival<br>- Added the matters pertaining to the immune response assessment and method thereof to the section on the variables in the efficacy assessment, based on what has been noted by the Ministry of Food and Drug Safety<br><br>11.1 Terms related to safety and definitions thereof<br>- If any of the subjects is admitted to a hospital as scheduled for treatment, it will not be considered a serious adverse event or adverse drug reaction, considering the condition of the subjects in this study
**18**	Ver.7.0	05.29.2017	06.30.2017	- Added that it is possible for the principal investigator to determine whether to administer the investigational drug and anticancer agent and the schedule thereof, according to the condition of each subject, in order to ensure the safety of the subjects, considering the condition of the subjects in this study
**19**	Ver.8.0	07.11.2018	07.23.2018	10.2.1 General principles for results analysis<br>- Clarified the standards concerning the analysis period and added the description of the matters concerning the interim analysis
