# Supplementary figures and images for: Autologous adoptive immune-cell therapy elicited a durable response with enhanced immune reaction signatures in patients with recurrent glioblastoma: An open label, phase I/IIa trial
Source: PLoS One. 2021 Mar 10;16(3):e0247293. doi: 10.1371/journal.pone.0247293 (PMC7946298; doi:10.1371/journal.pone.0247293)

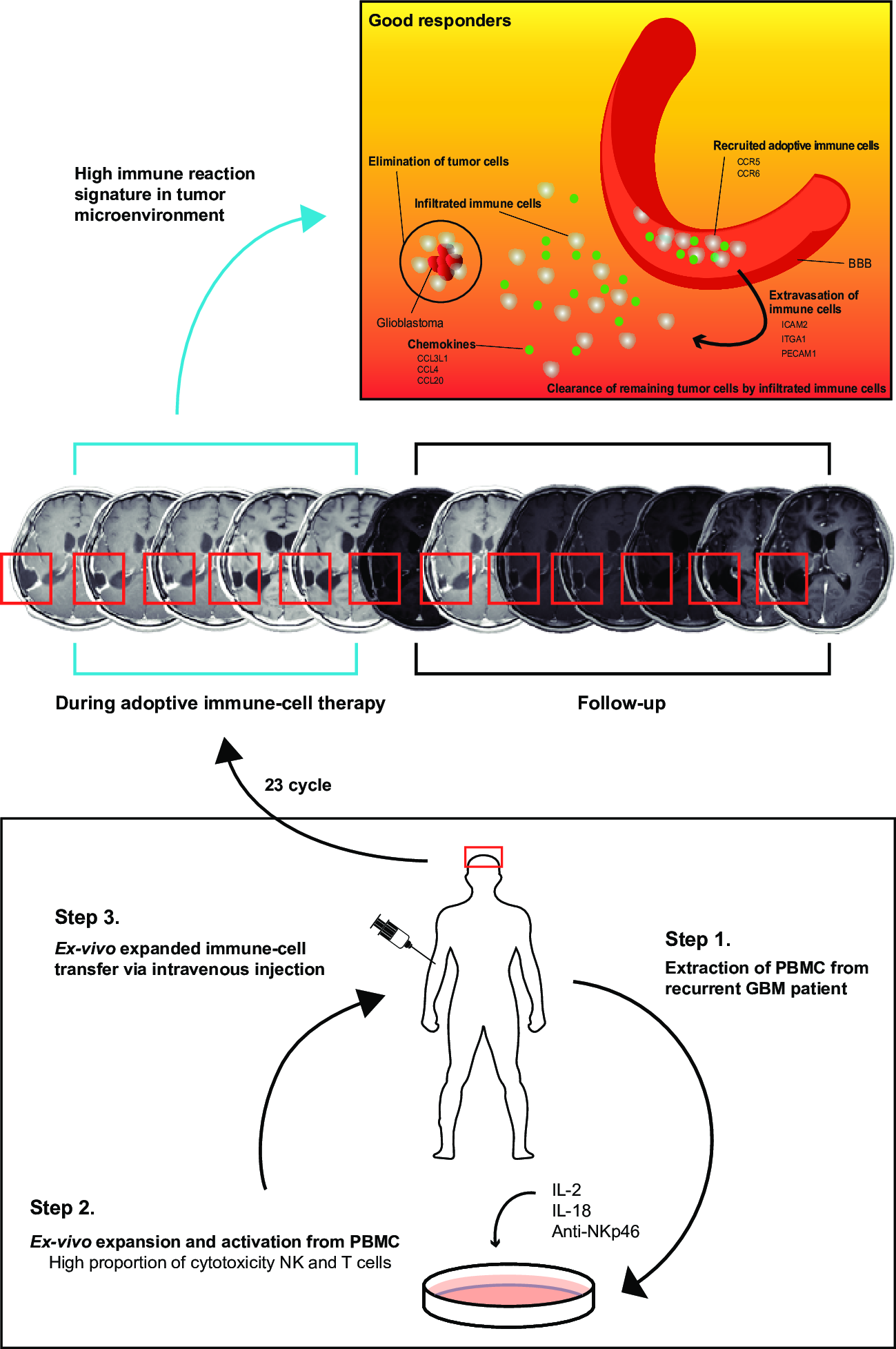

Supplement: S1 Fig — (TIF) [file pone.0247293.s004.tif]
